# Genes regulating gland development in the cotton plant

**DOI:** 10.1111/pbi.13044

**Published:** 2018-12-21

**Authors:** Madhusudhana R. Janga, Devendra Pandeya, LeAnne M. Campbell, Kranti Konganti, Stephany Toinga Villafuerte, Lorraine Puckhaber, Alan Pepper, Robert D. Stipanovic, Jodi A. Scheffler, Keerti S. Rathore

**Affiliations:** ^1^ Institute for Plant Genomics and Biotechnology Texas A&M University College Station TX USA; ^2^ Texas A&M Institute for Genome Sciences and Society Texas A&M University College Station TX USA; ^3^ Southern Plains Agricultural Research Center USDA‐ARS College Station TX USA; ^4^ Department of Biology Texas A&M University College Station TX USA; ^5^ Crop Genetics Research Unit USDA‐ARS Stoneville MS USA; ^6^ Department of Soil and Crop Sciences Texas A&M University College Station TX USA

**Keywords:** cotton (*Gossypium hirsutum*), glanding, RNA‐seq, comparative transcriptomics, VIGS, CRISPR/Cas9, transcription factors, cottonseed, gossypol, protein source, nutrition security

## Abstract

In seeds and other parts of cultivated, tetraploid cotton (*Gossypium hirsutum* L.), multicellular groups of cells lysigenously form dark glands containing toxic terpenoids such as gossypol that defend the plant against pests and pathogens. Using RNA‐seq analysis of embryos from near‐isogenic glanded (*Gl*_*2*_ *Gl*_*2*_ *Gl*_*3*_ *Gl*_*3*_) versus glandless (*gl*_*2*_ *gl*_*2*_ *gl*_*3*_ *gl*_*3*_) plants, we identified 33 genes that expressed exclusively or at higher levels in embryos just prior to gland formation in glanded plants. Virus‐induced gene silencing against three gene pairs led to significant reductions in the number of glands in the leaves, and significantly lower levels of gossypol and related terpenoids. These genes encode transcription factors and have been designated the ‘Cotton Gland Formation’ (*CGF*) genes. No sequence differences were found between glanded and glandless cotton for *CGF1* and *CGF2* gene pairs. The glandless cotton has a transposon insertion within the coding sequence of the *GoPGF* (synonym *CGF3*) gene of the A subgenome and extensive mutations in the promoter of D subgenome homeolog. Overexpression of *GoPGF* (synonym *CGF3*) led to a dramatic increase in gossypol and related terpenoids in cultured cells, whereas CRISPR/Cas9 knockout of *GoPGF* (synonym *CGF3*) genes resulted in glandless phenotype. Taken collectively, the results show that the *GoPGF* (synonym *CGF3*) gene plays a critical role in the formation of glands in the cotton plant. Seed‐specific silencing of *CGF* genes, either individually or in combination, could eliminate glands, thus gossypol, from the cottonseed to render it safe as food or feed for monogastrics.

## Introduction

Pigmented glands are one of the major characteristics of the tribe *Gossypieae,* belonging to family Malvaceae, that includes *Gossypium* L. and seven other genera (Fryxell, 1968). Most of the commercially grown cotton plants have dark glands in the subepidermal tissues of the aerial parts and in the cortex of roots that produce and store terpenoids such as gossypol (Stanford and Viehoever, 1918; Tian *et al*., 2018). While glands in the seed kernel and flower petals predominantly contain gossypol, those present in other parts of the plant contain additional terpenoids derived from the same biosynthetic pathway. Presence of these terpenoids serves a protective function against various insect pests and some pathogens (Hedin *et al*., 1992a; Lukefahr and Martin, 1966; Maxwell *et al*., 1965; Stipanovic *et al*., 1978a,b, 1999).

Research on the basis of gland formation in the cotton plant began following the discovery of ‘Hopi Moencopi’, a genotype cultivated well into the early 20^th^ century by the native Hopi peoples of Central Arizona (Fulton, 1938; McMichael, 1954, 1959, 1960). The bolls of this plant were reported to have variable number of pigment glands (Fulton, 1938). Since then, research conducted by several investigators implicated the roles of six genes in gland formation, however, only two major genes (*Gl*_*2*_ and *Gl*_*3*_) are believed to be involved in gland formation (Endrizzi *et al*., 1985; Gutierrez *et al*., 1972). In the tetraploid *G. hirsutum*, alleles *Gl*_*2*_, *Gl*_*2*_, *Gl*_*3*_ and *Gl*_*3*_ result in the glanded phenotype, whereas *gl*_*2*_, *gl*_*2*_, *gl*_*3*_ and *gl*_*3*_ are responsible for the glandless phenotype. Different combinations of dominant (*Gl*) and recessive (*gl*) alleles produced lesser number of glands with varying distribution in different parts of the plant at different stages of development (Gutierrez *et al*., 1972; Lee, 1965; McCarty *et al*., 1996; McMichael, 1960; Scheffler and Romano, 2008, 2012). Lee (1965) reported that *Gl*_*2*_ has approximately twice the expressivity of *Gl*_*3*_, with *Gl*_*2*_ originating from the ancestor that contributed to the A subgenome and *Gl*_*3*_ belonging to the D subgenome. *Gl*_*2*_ and *Gl*_*3*_ were localized to the A12 and D12 chromosomes of *G. hirsutum* respectively (Lee, 1965; Percy *et al*., 2015; Samora *et al*., 1994). During the course of the current investigation, Ma *et al*. (2016) published a report describing the identification of a gene named *GoPGF* (Gossypium Pigment Gland Formation) through map‐based cloning approach using a glandless (dominant) *G. barbadense* mutant (*Gl*_*2*_^*e*^) that was originally created in Egypt by mutagenizing radiation (Afifi *et al*., 1966). This was followed by sequence analysis of the two homeologs of this gene in a recessive glandless mutant of *G. hirsutum*. The origins of this glandless mutant were not described. The causative mutations in this glandless cotton were presumed to be insertions of a single nucleotide into the coding sequences of each of the two *GoPGF* homeologs resulting in premature translation termination. The authors designated the *GoPGF* gene on chromosome A12 as the *Gl*_*2*_ gene and its homeolog on chromosome D12 as the *Gl*_*3*_ gene.

In this study that began in May, 2015, we utilized a more direct, RNA‐seq based approach to identify the genes that are involved in gland formation in cotton. In the developing cotton embryo, gland formation begins around 15 days post‐anthesis (dpa) (Reeves and Beasley, 1935; Scheffler *et al*., 2014). We performed RNA‐seq analysis to identify differentially expressed genes in 14‐, 16‐ and 32‐dpa embryos from glanded (STV GL; GVS4) and glandless (STV gl; GVS5) cotton (Stoneville 7A; *Gossypium hirsutum* L.) (Scheffler and Romano, 2012). The genes that were expressed at significantly higher levels in the 14‐dpa embryos of the glanded plants, and thus deemed to have possible regulatory functions in gland development, were subjected to virus‐induced gene silencing (VIGS) to ascertain whether these played a role in gland formation. These analyses resulted in the identification of three genes that played critical roles in gland formation. VIGS targeting of any of these three genes not only resulted in inhibition of gland formation, but also in the reduction in gossypol and related terpenoids in the leaves of treated plants. These have been designated Cotton Gland Formation (*CGF*) genes. We have determined the genomic sequence of these genes in the A and D subgenomes of glanded and glandless cotton plants. Only the *GoPGF* (synonym *CGF3*) gene homeologs show differences between the glanded and glandless cotton plants. Furthermore, *GoPGF* (synonym *CGF3*) knockout lines showed complete absence of glands. We discuss the implications of suppressing the expression of *CGF* genes in a seed‐specific manner to obtain cotton plants that produce seeds with significant reduction in the number of glands (and thus gossypol) so that the immense protein resource available in cottonseed can be safely used either as feed for monogastric animals or directly as food for humans to improve their nutrition security.

## Results

### Comparative transcriptome analysis of developing embryos from glanded and glandless cotton plants reveals several genes associated with gland formation

Cotton embryos at 14‐, 16‐ and 32‐dpa were used for transcriptome analysis; these encompassed a stage preceding visible gland formation to one of active gland‐filling. Embryos from near‐isogenic lines of Stoneville 7A referred to as Stoneville 7A Glanded (STV GL, GVS4) and Stoneville 7A glandless (STV gl, GVS5) were compared with the aim of finding differentially expressed genes. As shown in Figure S1a, no glands were observed in 14‐dpa embryos of GVS4, however, at 16‐dpa some glands can be seen in the embryos from this line (shown with arrows in Figure S1b). Gossypol, the major storage terpenoid of seed‐glands, can be detected in the embryos of glanded cotton plants around 24‐dpa and later (Scheffler *et al*., 2014). No glands were detected in line GVS5 embryos at any stage of development (Figures S1c and d). RNA was isolated from three replicate samples of 14‐, 16‐ and 32‐dpa embryos each from GVS4 and GVS5. RNA‐seq was performed on these three different developmental stages from two different glanding types to give six different tissues and a total 377 million quality‐filtered paired‐end reads were obtained. Out of these, 273 million unique reads (72.13%) were mapped to the reference genome (Zhang *et al*., 2015), and 22.82% of them mapped more than one time. Overall, 94.95% of reads were mapped to the reference genome. Only the uniquely mapped reads were used to measure transcript abundance. Tissue‐wise data for the mapped reads are given in Table S1.

**Figure 1:**
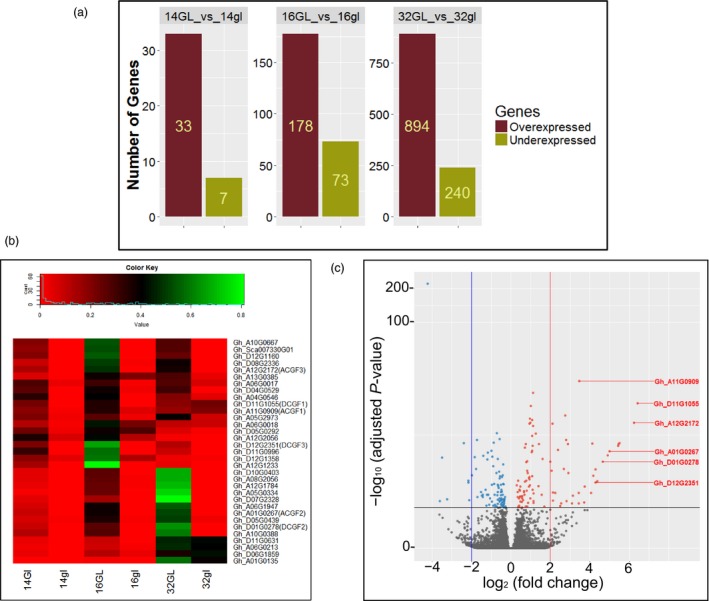
Differentially expressed genes in developing embryos from glanded (GL) and glandless (gl) cotton plants. (a) Differentially expressed genes identified in pairwise comparisons between GL and gl embryos at 14‐, 16‐ and 32‐days post‐anthesis (dpa). (b) Heatmap visualization of expression of the 33 genes that were expressed at higher levels in the 14‐dpa glanded embryos during the course of embryo development. (c) Volcano plot showing gene expression differences between 14‐dpa embryos from glanded and glandless cotton. Genes with absolute fold change ≥2 (red line) and *P* < 0.05 are indicated as red dots. The *CGF* gene homeologs are shown in red coloured letters.

To ascertain transcript abundance, only the uniquely mapped reads were quantified using HTSeq‐count program to obtain read count values for all the annotated 70,478 genes in *G. hirsutum* (Zhang *et al*., 2015). Of these, 57,510 genes were expressed in at least one of the samples analysed, which were further considered for downstream analysis. At least, 30 million unique reads were counted for each tissue (every replicate had 10 million or more read counts). DESeq2 program was used to identify differentially expressed genes (log fold change ≥2 and FDR <0.05). Figure 1 shows the number of genes that are differentially expressed in the glanded vs. glandless embryos at different time points. At 14 dpa, a small number of genes were differentially expressed, with only 33 genes expressed at higher levels in the glanded embryos compared to glandless embryos (Table S2). Seven genes were expressed at lower levels in the glanded embryos. At 16 dpa, 178 genes were expressed at higher levels and 73 genes at lower levels in the glanded embryos (Data S1). At 32 dpa, 894 genes were expressed at higher levels and 240 genes at lower levels in the glanded embryos (Data S1). Table S2 shows the 33 genes that were expressed at significantly higher levels in the 14‐dpa embryos of glanded cotton plant. This stage precedes 1–2 days before the glands become visible, therefore we focused on this stage of development to identify the genes that would presumably be responsible for and involved in gland formation.

### Virus‐induced gene silencing validates the involvement of three genes in gland formation

**Figure 2:**
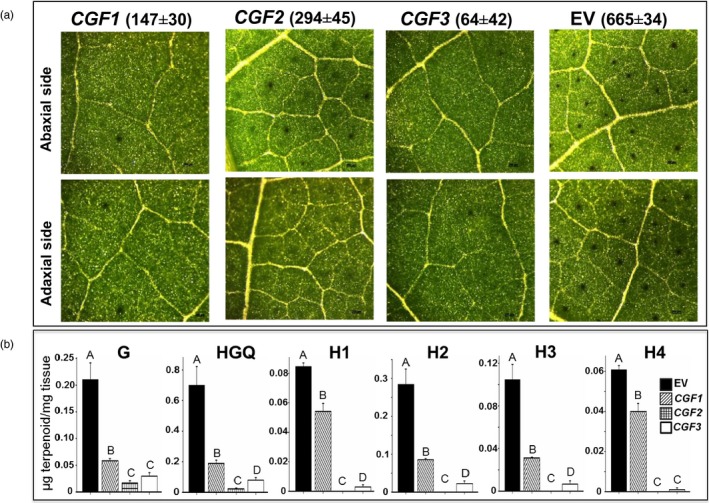
Effect of virus‐induced gene silencing (VIGS) of the *CGF1*,*CGF2* and *GoPGF* (synonym *CGF3*) genes on gland formation. (a) Microscopic images of leaves (magnification: X42). Mean gland number per unit area (6.45 cm^2^) ±SE shown above the respective image. (b) terpenoid levels in the leaves. EV: empty vector control; G: gossypol; HGQ: hemigossypolon; H: heliocides. The values indicated by bars within a group are significantly different at *P* ≤ 0.05 if labelled with different letters.

Virus‐induced gene silencing is a rapid and simple method to transiently silence a target gene in the young emerging leaves of a plant. Therefore, we used this method to silence individual genes in cotton seedlings in order to determine their role in gland development. The subset of genes that were predicted to encode proteins of regulatory function were subjected to VIGS, based on the assumption that one or more of these might play an important role in gland formation. In cases where both of the homeologs were found to be expressed at higher levels in the glanded embryos, a single VIGS construct was used to target both homeologs for silencing – a viable approach given the high degree of sequence similarity (over 95%) between the two (Table S2). In all our VIGS experiments, the gene‐silencing efficacy was confirmed by targeting *GhCLA* gene that results in albino leaf phenotype (Figure S2). Target sequences, ranging in size from 357 to 634 bp, were amplified using a set of primers listed in Table S3. Of the ten genes targeted in this manner, we observed negative effects on gland formation after silencing of three gene pairs, designated *Cotton Gland Formation* (*CGF*) genes. We observed a dramatic reduction in the number of glands in response to silencing of both the Gh_A11G0909/Gh_D11G1055 gene pair (*CGF1*; 78% reduction) and the Gh_A12G2172/Gh_D12G2351 [*GoPGF* (synonym *CGF3*); 90% reduction] (Figures 2a, and S3). The reduction in gland numbers in the newly emerging leaves was observed starting at 2‐weeks post‐infiltration. At 21 days post‐infiltration, the leaves were scanned and the gland number was quantified. Figure 2a shows the representative images of the leaves from the plantlets that had undergone VIGS treatment as compared to an empty vector control. The *CGF1* and *GoPGF* (synonym *CGF3*) gene pairs both encode basic Helix‐Loop‐Helix (bHLH) transcription factors. VIGS silencing of another gene pair, Gh_A01G0267/Gh_D01G0278 (*CGF2*), which encodes NAC‐family transcription factors, did not show such a dramatic reduction in the number of glands compared to the lines that were silenced at the *CGF1* and *GoPGF* (synonym *CGF3*) loci. However, the visual and microscopic appearance of the glands in *CGF2*‐targeted leaves were qualitatively different from glanded cotton in terms of colour intensity and structure, as though their development was adversely affected (Figure 2a). No effects on gland number or appearance were observed with the remaining seven VIGS constructs and therefore those respective genes were not investigated further.

Unlike the glands in cottonseed that contain mainly gossypol, the glands in leaves contain not only gossypol, but also hemigossypolon and heliocides that are derived from the same biosynthesis pathway. Thus, reduced number of functional glands would be expected to result in lower amounts of these terpenoids in the leaves of cotton plants that have undergone VIGS against the *CGF* genes. Therefore, we conducted HPLC analysis to measure the levels of these terpenoids in the leaves. Indeed a significant reduction in the level of gossypol and related terpenoids (hemigossypolon and heliocides) was observed in the leaves of plants that were subjected to VIGS‐mediated silencing of *CGF1*,* CGF2* or *GoPGF* (synonym *CGF3*) genes (Figure 2b). Since the terpenoids are usually produced and stored in the glands, the reduced levels of these compounds likely result from fewer glands or fewer functional glands. Thus, based on the results from RNA‐seq analysis and VIGS experiments, we identified three transcription factors and their homeologs that play an important, positive role in the formation of glands in the cotton plant. The *CGF* gene homeologs of the A subgenome will be referred to as *ACGF* and those of the D subgenome as *DCGF* in the remaining text, figures and tables in this report.

### qRT‐PCR validates the involvement of three *CGF* gene pairs in gland formation

**Figure 3:**
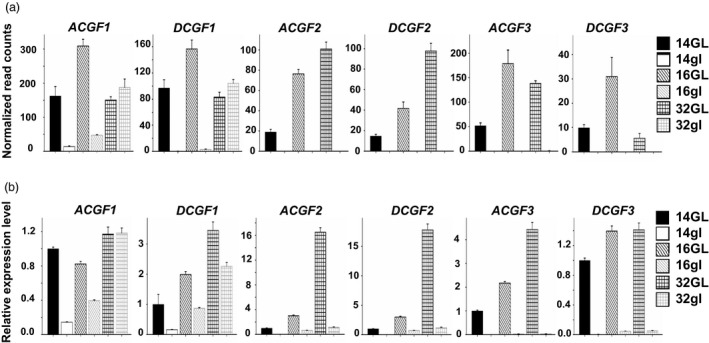
Expression levels for the three *CGF* genes in A and D subgenomes at 14‐, 16‐ and 32‐days post‐anthesis embryos from glanded (GL) and glandless (gl) cotton plants. (a) Mean normalized read counts of three biological replicates, based on RNA‐seq analysis; (b) qRT‐PCR results showing relative expression levels.

Transcript abundance for the *CGF* genes (and the respective homeologs) in glanded (GVS4) and glandless (GVS5) embryos at different developmental stages is depicted as normalized read counts in Figure 3a. To validate the RNA‐seq expression profile of these genes, qRT‐PCR was performed using the same set of RNA samples that was used to perform RNA‐seq analysis. PCR efficiencies of the three *CGF* gene pairs and internal control histone gene were determined following the protocol described by Livak and Schmittgen (2008). The efficiencies for each of the *CGF* homeologs and histone gene were in the range of 2.0 to 2.2 confirming the validity of qPCR results (Figure S4). Results obtained from qRT‐PCR analysis confirmed the expression profiles of the three *CGF* genes that were observed with the RNA‐seq analysis (Figure 3b). Expression of these *CGF* genes in the glandless embryos was indeed substantially lower than that of the glanded embryos at 14‐ and 16‐dpa stages of development.

### Sequencing of *CGF* genes reveals the underlying cause of glandless phenotype

**Figure 4:**
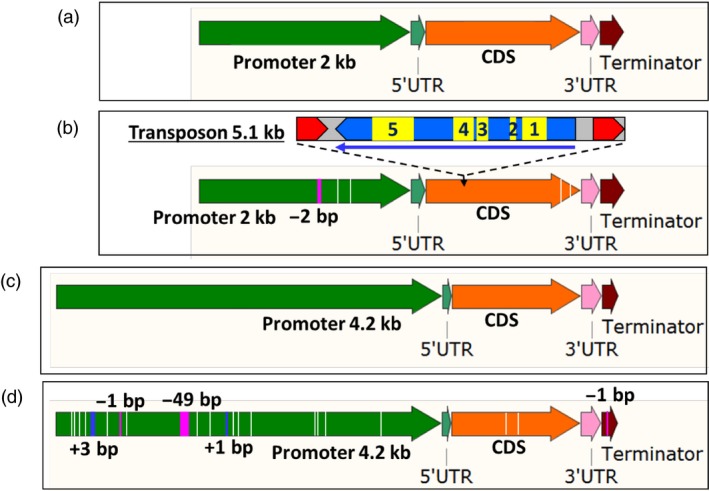
Illustration showing differences between glanded and glandless cotton for *GoPGF* (synonym *CGF3*) gene in A‐ and D‐subgenome. (a) *ACGF3* in glanded (GVS4) cotton. (b) *ACGF3* in glandless (GVS5) cotton showing four SNPs (white lines), one deletion (pink line) and a transposon insertion. The Copia‐like, retrotransposon is 5.1 kb in size. Red arrows represent direct repeats. The long thin arrow indicates direction and size of an open reading frame. Functional domains are: 1: gag‐polypeptide of LTR copia‐type, 2: GAG‐pre‐integrase domain, 3: Integrase core domain, 4: Reverse transcriptase (RNA‐dependent DNA polymerase), 5: Ty1/Copia family of RNase HI in long‐term repeat retro elements. (c) *DCGF3* in glanded (GVS4) cotton. (d) *DCGF3* in glandless (GVS5) cotton showing 17 SNPs (white lines), three deletions (pink lines) and two insertions (blue lines).

The results for the expression profile of *CGF* genes show that these genes have little or no activity in the glandless embryos, especially at 14‐dpa stage. In order to understand the reasons for these differences, we sequenced each of these genes and their homeologs from glanded and glandless cotton plants. Large PCR fragments were amplified from the genomic DNA of glanded and glandless cotton plants using specific primers (Table S4) that can differentiate between the A and D subgenome homeologs of each of the *CGF* genes. These amplicons included approx. 2 kb of promoter region (4.2 kb in case of *DCGF3*), UTRs, introns (if present), exons and terminator. No sequence differences were found between glanded and glandless cotton plants in either *CGF1* or *CGF2* genes or in their respective homeologs (Figures S5–S8). Major sequence differences between the glanded and glandless cotton plants were observed in both *GoPGF* (synonym *CGF3*) gene homeologs (Figure 4). The glandless line (GVS5) showed a 5.1 kb transposon insertion between 362 and 363 bp of the coding sequence of the *ACGF3* gene (Gh_A12G2172; Figures 4a,b and S9). In addition, there were two SNPs and a 2‐bp deletion in the promoter sequence, and two SNPs in the coding sequence of Gh_A12G2172 gene in the glandless GVS5. The coding sequence of the *DCGF3* gene (Gh_D12G2351) of the glandless mutant (GVS5) has two SNPs (one synonymous and one nonsynonymous) compared to the wild‐type glanded cotton (GVS4). In addition, the terminator sequence of the *DCGF3* from the glandless mutant line (GVS5) has one base pair deletion. However, the significant differences in the *DCGF3* gene between glanded and glandless cotton were in the promoter (Figures 4c,d and S10). The ~4.2 kb promoter region of this gene in the glandless mutant (GVS5) had fifteen SNPs, two deletions (1 and 49 bp long) and two insertions (1 and 3 bp) compared to the glanded cotton (GVS4). Interestingly, the *CGF3* gene that we have identified and sequenced from the glanded cotton, GVS4, is the same gene designated as *GoPGF* by Ma *et al*. (2016). However, the underlying cause of mutation responsible for glandless phenotype in each case is different, as discussed later.

### Both the A‐ and D‐subgenome homeologs of *GoPGF* (synonym *CGF3*) gene are expressed in the embryos of glanded cotton

There are some genes in an allotetraploid such as *G. hirsutum* in which one homeolog for a particular gene is expressed, whereas the other remains silent in a given tissue (Adams *et al*., 2003; Grover *et al*., 2012). RNA‐seq results showed that while both the A‐ and D‐subgenome homeologs of the *GoPGF* (synonym *CGF3*) gene were expressed in the developing embryos of the glanded cotton, the *DCGF3* was less active (Figure 3a). In order to further confirm whether both the homeologs of the *GoPGF* (synonym *CGF3*) gene are expressed in the embryos of glanded cotton, a PCR amplicon was generated with a primer set which can amplify both A and D‐subgenome homeologs using the cDNA from 14‐dpa embryos. Direct sequencing of this amplicon clearly showed the expected SNPs (Figure S11) thus confirming the results from RNA‐seq analysis and qRT‐PCR showing that both the *GoPGF* (synonym *CGF3*) homeologs are expressed in the embryos of glanded cotton.

### Promoter sequence analysis and activity evaluation of the D subgenome *GoPGF* (synonym *CGF3*) homeolog reveals the basis for its inactivity in glandless cotton

As described earlier, the *GoPGF* (synonym *CGF3*) homeologs in both the A and D subgenomes show no expression in the embryos of glandless GVS5 (Figure 3). The undetectable level of expression of the *GoPGF* (synonym *CGF3*) gene in the A subgenome is likely due the insertion of the 5.1 kb transposon (Figure 4b). As we had done for the other *CGF* genes and their homeologs, at first, we amplified only the ~2 kb of the promoter (2009 bp), 5’‐UTR (97 bp), the coding sequence, 3’‐UTR and 182 bp of the terminator region of the *DCGF3* gene from glanded and glandless cotton. We observed four SNPs in the promoter region and two SNPs in the coding sequence between glanded and glandless cotton. To investigate whether these SNPs in the promoter region were responsible for the lack of transcripts in the glandless GVS5, we assembled promoter::*gusA* constructs using ~2.1 kb long promoter (including 5’‐UTR) sequences from the *DCGF3* gene of GVS4 and GVS5. *Agrobacterium tumefaciens* cells containing the reporter gene construct were used to transform hypocotyl segments of cotton seedlings. Callus tissue growing on hypocotyl segments following transformation were examined histochemically for GUS activity, 30 days after transformation. The results presented in Figure S12 show clearly that the D subgenome *GoPGF* (synonym *CGF3*) gene promoter sequences (~2.1 kb) from the glanded and glandless cotton were equally active. It is possible that the ~2.1 kb sequence does not fully represent the entire promoter region of this gene and that important regulatory elements reside further upstream. We therefore isolated a longer, ~4.2 kb of the promoter region of the *DCGF3* gene from glanded (GVS4) and glandless (GVS5) cotton. As described earlier, the ~4.2 kb promoter region of this gene in the glandless mutant (GVS5) showed significant mutations, including fifteen SNPs, two deletions (1 and 49 bp long) and two insertions (1 and 3 bp) compared to the glanded cotton (GVS4). In order to examine whether these sequence differences in the glandless cotton were responsible for the lack of expression of the *DCGF3* gene, we assembled reporter gene constructs as described above. Callus tissues growing from the transformed cotyledon, hypocotyl and petiole explants were examined histochemically for GUS activity, 5 weeks after *Agrobacterium*‐mediated transformation with each of the constructs. The results from this analysis are shown in Figure S13. While the tissue transformed with a construct wherein the *gusA* gene was under the control of *DCGF3* promoter from glanded cotton showed strong GUS activity, the callus originating from explants following transformation with glandless *DCGF3* promoter construct showed drastic reduction in reporter gene activity. The results suggest that the lack of *DCGF3* transcripts in the glandless (GVS5) cotton is due to the attenuation of the activity of its heavily mutated promoter.

### Sequencing of *GoPGF* (synonym *CGF3*) gene from four additional glandless lines reveals the nature of mutations

As mentioned above, the underlying cause for the glandless trait in the recessive, *G. hirsutum* mutant as proposed by Ma *et al*. (2016) was the insertion of a single nucleotide in the coding sequence of each of the two *GoPGF* homeologs. However, the *GoPGF* (synonym *CGF3*) gene identified in this study showed a 5.1 kb, copia‐like, retrotransposon in the A subgenome homeolog and several SNPs, insertions and deletions in the promoter of the D subgenome homeolog of the glandless mutant GVS5 (Figures 4, S8 and S9). In order to further explore the genetic basis of the glandless phenotype in cotton germplasm, four additional glandless cotton lines that have been developed by other breeders [Acala GLS, NM‐13P1088, NM‐13P1115 and NM‐13P1117 strains (Bowman *et al*., 2006; Zhang *et al*., 2014)] were examined for allelic variation in the *GoPGF* (synonym *CGF3*) gene pair by PCR amplification and sequencing. The results showed that the glandless Acala GLS and NM‐13P1088 had the same transposon insertion in the *ACGF3* gene that we had discovered in the GVS5 line. These results agree with the pedigree information available for these lines (Bowman *et al*., 2006). The glandless source of GVS5 is STV 7A gl which is believed to have C6‐5 in its pedigree, and one of C6‐5 parents is Hopi Moencopi. Bowman *et al*. (2006) traces Acala GLS back to Hopi Moencopi and NM‐13P1088 has Acala GLS as its glandless parent. As mentioned earlier, Hopi Moencopi is a glandless source discovered and described in the mid‐twentieth century (Fulton, 1938; McMichael, 1954, 1959, 1960). The other two glandless cotton lines (NM‐13P1115 and NM‐13P1117) had a total of three SNPs in the coding region of *ACGF3* gene, including two synonymous and one nonsynonymous, at residue 43, which alters an alanine to valine. Thus, these two lines have the same dominant mutation *Gl* ^*e*^_*2*_ obtained through irradiation to create the Egyptian glandless cotton cultivar ‘Bahtim 110’, as reported previously (Kohel and Lee, 1984; Ma *et al*., 2016). Their pedigrees show that the glandless parent for both NM‐13P1115 and NM‐13P1117 was an experimental line that had Bahtim 110 as one of its parents.

### CRISPR/Cas9‐mediated knockout of *CGF2* genes reduces gland density and terpenoids in the leaves of mutants, and knockout of *GoPGF* (synonym *CGF3*) genes results in glandless phenotype

**Figure 5:**
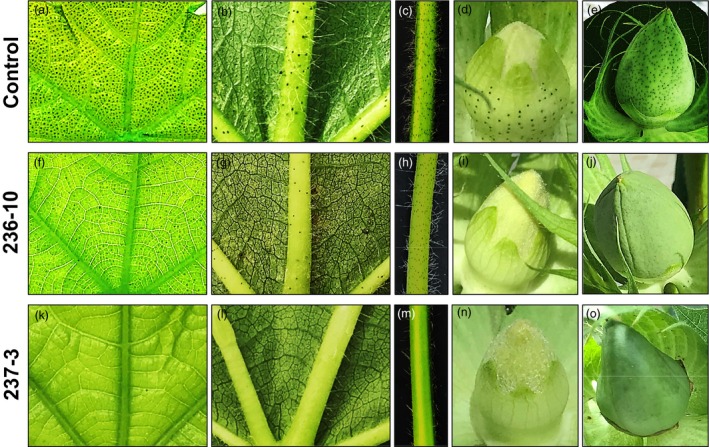
Mutant lines showing the effect of CRISPR/Cas9‐mediated knockout of *CGF2* (236‐10) and *GoPGF* (synonym *CGF3*) (237‐3) genes on gland formation in T0 plants in comparison to glanding pattern seen in a wild‐type, control cotton plant. (a,f,k) leaf image (adaxial), illuminated from underside. (b,g,l) leaf image (abaxial), illuminated from above. (c,h,m) leaf petiole. (d,i,n) unopened floral bud. (e,j,o) developing cotton boll.

We conducted additional experiments using the CRISPR/Cas9 system to knockout *CGF2* and *GoPGF* (synonym *CGF3*) genes in order to validate their role in gland formation. In each case, both A and D homeologs were targeted for knockout. Since *CGF1* homeologs are active in the 32‐dpa embryos of both glanded and glandless plants (Figure 3), and therefore possibly also involved in other activities, so these were not targeted for CRISPR/Cas9‐mediated knockout. Four lines from targeting of the *CGF2* gene (LCT236 construct) and nine lines from targeting of the *GoPGF* (synonym *CGF3*) gene (LCT237 and LCT238 constructs) were recovered. Detailed biochemical and molecular analyses were performed on two lines in each case. The leaves obtained from the regenerated plants (T0 generation) were examined for their terpenoid content. Results presented in Table 1 show significant reduction in terpenoid levels in the leaf tissues of the *CGF2* mutants (236‐8 and 236‐10). In line with our observations in VIGS experiments, the number of glands was substantially reduced in various parts of the mutant lines (Figure 5). The glands that were present were smaller and appeared abnormal as shown in the high magnification images presented in Figure S14. The mutations obtained in these two *CGF2*‐targeted lines are shown in Figure S15. Virtually no gossypol was detected in the leaves of *GoPGF* (synonym *CGF3*) knockout plants (237‐3 and 237‐4; Table 1) and all parts of the plants examined were devoid of glands (Figure 5). The mutations observed in these two *GoPGF* (synonym *CGF3*)‐targeted lines are shown in Figure S16. The trait created by CRISPR‐Cas9‐mediated knockout of *CGF2* and *GoPGF* (synonym *CGF3*) genes is heritable in the T1 generation as illustrated in Figure S17. These results confirm that *CGF2* and *GoPGF* (synonym *CGF3*) genes play important roles in the development of glands in the cotton plant. Furthermore, a completely glandless phenotype observed in the *GoPGF* (synonym *CGF3*) knockout mutants validates the primacy of this gene as a key regulator of gland development.

**Table 1:** Terpenoid values in the leaves of mutant lines generated by CRISPR/Cas9 mediated knockout of *CGF2* (236‐8 and 236‐10) and *GoPGF* (synonym *CGF3*) (237‐3 and 237‐4) genes in comparison to wild‐type control

	Terpenoids (μg terpenoid/mg tissue)					
	HGQ	G	H1	H2	H3	H4
Wild‐type	0.33	0.18	0.07	0.28	0.11	0.04
Line 236‐8	0.01	0.02	0.00	0.00	0.00	0.00
Line 236‐10	0.00	0.01	0.00	0.00	0.00	0.00
Line 237‐3	0.00	0.00	0.00	0.00	0.00	0.00
Line 237‐4	0.00	0.00	0.00	0.00	0.00	0.00

G, gossypol; HGQ, hemigossypolon; H, heliocides.

### Overexpression of A subgenome *GoPGF* (synonym *CGF3*) in cotton callus tissue significantly increases the terpenoid levels

**Figure 6:**
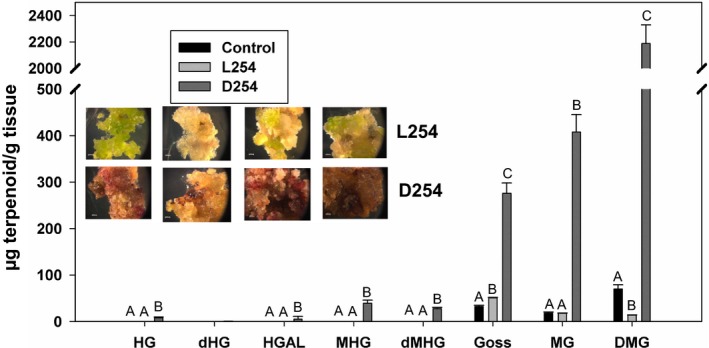
Terpenoid levels in cotton callus cultures, obtained following transformation with A subgenome *GoPGF* (synonym *CGF3*) overexpression construct. L254: light‐coloured callus lines; D254 dark‐coloured callus lines; Control: nontransgenic callus. HG: Hemigossypol,; dHG: Desoxyhemigossypol; HGAL: Hemigossylic acid lactone; MHG: Methoxyhemigossypol; dMHG: Desoxymethoxy‐hemigossypol; Goss: Gossypol; MG: Methoxygossypol; DMG: Dimethoxygossypol. The values indicated by bars within a group are significantly different at *P* ≤ 0.05 if labelled with different letters.

While glands are present in most parts of the cotton plants, these have never been observed in callus cultures. We wanted to examine the impact of overexpressing *GoPGF* (synonym *CGF3*) gene under the control of a constitutive promoter, such as the CaMV 35S promoter. Therefore, we assembled an overexpression vector using the *ACGF3* coding sequence driven by this promoter and transformed cotton seedling explants using the *Agrobacterium* method. Individual transgenic events (in the form of small, kanamycin‐resistant calli) developing on the explants were excised and further cultured as per our laboratory protocol. When observed after 4 months, a majority of these events had turned unusually dark brown, while a few events remained light pale‐green colour similar to what transgenic callus lines, transformed with any other gene, usually appear at this stage. We suspected that the dark‐coloured events were expressing the transgenic *ACGF3* gene, whereas the lighter‐coloured ones were not. In order to examine this possibility, qRT‐PCR was performed on these two types of culture lines. Results presented in Figure S18 show that the dark‐coloured culture lines indeed showed higher level transcription of *GoPGF* (synonym *CGF3*) gene compared to the lighter‐coloured lines that showed activities similar to the nontransgenic control cultures. This molecular analysis was followed by an additional biochemical analysis in which we examined the two types of culture lines for their terpenoid content. Terpenoids that are usually found in glands, such as gossypol, were detected at significantly higher levels in the dark‐coloured cultures compared to the light‐coloured ones and the nontransgenic callus cultures (Figure 6). In addition to gossypol, some other terpenoids were found either exclusively (hemigossypol, desoxyhemigossypol, hemigossylic acid lactone, methoxyhemigossypol and desoxymethoxyhemigossypol) or at significantly higher levels (methoxygossypol and dimethoxygossypol) in the dark‐coloured culture lines.

## Discussion

Studies on cotton embryo development and gland formation by Reeves and Beasley (1935) and Scheffler *et al*. (2014) indicate that gland formation starts around 15 dpa. A similar timing of gland formation was observed in our study. No glands were observed in the embryos at the 14‐dpa stage in the greenhouse‐grown, glanded cotton (STV GL) GVS4 (Figure S1a), however, at 16‐dpa stage, the glands were clearly visible under a microscope in the embryos from this glanded line (Figure S1b). No glands were observed in the glandless (STV gl) GVS5 at any stage of embryo development. On the basis of this information, we conducted transcriptome analyses on embryos at 14‐, 16‐, 32‐dpa stage of development obtained from glanded, GVS4 and glandless, GVS5 near‐isogenic cotton lines. RNA‐seq analysis revealed that 33 genes were expressed at higher levels in the glanded embryos at 14 dpa compared to their counterparts from the glandless plants. Since gossypol biosynthesis does not begin in the embryos before 20 dpa (Scheffler *et al*., 2014), genes involved in terpenoid biosynthesis such as the one encoding δ‐cadinene synthase were not active in the embryos of the glanded plants at 14 dpa. Because we found no visible glands in the embryos at 14 dpa, we hypothesized that comparative transcriptomics at this time‐point would reveal the identity of the genes that play an important role in initiating gland formation. The later stages of embryo development are likely to reveal the genes that are involved in gland maturation and biosynthesis of secondary metabolites, including gossypol (Huchelmann *et al*., 2017).

RNA‐seq proved to be a rather straightforward and useful technique in identifying a number of genes that were either solely expressed or more highly expressed in the embryos (14 dpa) of glanded cotton compared to those in the glandless cotton. VIGS was used against ten different genes that were predicted to encode proteins with regulatory functions to ascertain their involvement in gland formation. VIGS targeting of three different genes and their homeologs (designated *CGF*) significantly reduced the number of glands, and the terpenoids that are stored within, in the young emerging leaves of a cotton plantlet. Further, qRT‐PCR results on each of these genes validated the RNA‐seq analysis in terms of relative expression levels for the homeologs of the three *CGF* genes.

Sequencing of the respective homeologs of *CGF1* and *CGF2* did not show any differences between the glanded and glandless cotton. However, the *ACGF3* gene in the glandless cotton had a 5.1 kb transposon insertion within its coding sequence, thus accounting for its silencing (Figures 4 and S9). The D subgenome homeolog of *GoPGF* (synonym *CGF3*) gene in the glandless cotton showed two SNPs in the coding sequence and one SNP in the terminator between glanded and glandless cotton. However, the ~4.2 kb upstream regulatory sequence showed some major differences in the glandless cotton, including fifteen SNPs, two deletions (1 and 49 bp long) and two insertions (1 and 3 bp), compared to the glanded cotton (Figures 4 and S10). Comparative promoter activity analysis of this region between glanded and glandless cotton showed that the heavily mutated, *DCGF3* gene promoter from the glandless cotton was substantially weakened.

No sequence differences between the glanded and glandless cotton were observed for the *CGF1* and *CGF2* genes and their respective homeologs. However, the fact that VIGS‐mediated downregulation of these genes did have a negative impact on the gland numbers and terpenoid levels, the respective encoded proteins probably do play an important role in gland formation. Particularly, the importance of *CGF2* in gland development is supported by the fact that both VIGS and CRISPR/Cas9‐mediated knockout of this gene not only had a negative effect on gland numbers, the glands that were visible appeared abnormal and the terpenoid content of the leaves was greatly reduced. Of the three *CGF* gene pairs, *GoPGF* (synonym *CGF3*) genes seem to play the most critical role in gland development. Validation for this notion comes from the following results: 1) complete absence of *GoPGF* (synonym *CGF3*) transcripts in the glandless embryos at all stages of development, 2) significant reduction in leaf glands and terpenoids by VIGS treatment, 3) totally glandless phenotype and absence of terpenoids in the knockout lines created by CRISPR/Cas9‐mediated mutations. The two *GoPGF* (synonym *CGF3*) homeologs were localized on A12 and D12 chromosomes of *G. hirsutum*.

As mentioned earlier, a report by Ma *et al*. (2016) describes the identification of a gene, named *GoPGF*, using a glandless (dominant) mutant (*G. barbadense*, Hai‐1) that was derived from a mutant (*Gl*_*2*_^*e*^) originally created in Egypt by mutagenizing radiation (Afifi *et al*., 1966). The authors proposed that one amino acid change from alanine to valine at residue 43 in the protein as a result of substitution of ‘C’ to ‘T’ at base 128 in the coding sequence of the A subgenome *GoPGF* was the underlying cause for this dominant mutation. These authors followed this work by sequence analysis of the two homeologs of this gene in a recessive glandless mutant of *G. hirsutum* of unknown origin. They found that this glandless cotton had a single nucleotide insertion in the coding sequence of each of the two *GoPGF* homeologs resulting in premature translation termination (insertion of a ‘T’ between 735 and 736 bp in A subgenome homeolog and insertion of an ‘A’ between 916 and 917 in the D subgenome homeolog), thus accounting for the glandless trait. In our investigation of *GoPGF* (synonym *CGF3*) gene(s) of GVS5, the cause of mutation is entirely different. The basis for the silencing of the A subgenome *GoPGF* (synonym *CGF3*) is likely due to the insertion of a 5.1 kb transposon, whereas the D subgenome *GoPGF* (synonym *CGF3*) gene promoter of the glandless cotton has undergone extensive mutations, thus silencing the gene activity.

The *ACGF3* is localized on chromosome A12 while its homeolog the *DCGF3* is present on chromosome D12. Here, we have provided substantial evidence that these two homeologs are the main genes controlling the development of glands in cotton plants and, based on chromosomal location, correspond to the *Gl*_*2*_ and *Gl*_*3*_ loci described by several geneticists previously (Lee, 1965; Percy *et al*., 2015). The results from RNA‐seq analysis also show that while both homeologs of the *GoPGF* (synonym *CGF3*) gene are expressed in the developing embryos of glanded cotton, the A subgenome homeolog is more active, thus providing confirmation for the earlier contention that *Gl*_*2*_ is expressed at higher level compared to *Gl*_*3*_ gene in glanded cotton (Lee, 1965). Hovav *et al*. (2015) conducted global transcriptome analysis on developing seeds of *G. hirsutum* (TM1) at 10, 20, 30 and 40 dpa and found that about 20% of the genes showed homeolog expression bias. This group also observed that the *ACGF3* homeolog in TM1 had higher level of expression than *DCGF3* in the seeds at 30‐ and 40‐dpa.

While cotton is grown for its fibre, the plant produces ~1.6 X more seed by weight. In addition to the oil, cottonseed also contains ~23% protein. Thus, global cottonseed production (~45 million metric tons, MMT) containing ~10 MMT of protein can potentially meet the basic protein requirements of ~550 million people (Rathore *et al*., 2017). However, because of the presence of toxic gossypol in the seed glands (Gadelha *et al*., 2014; Risco and Chase, 1997), this abundant resource cannot be used for food or even as feed for monogastric animals. Whole cottonseed and cottonseed meal are used simply as feed for older cattle that are highly inefficient in converting feed protein into meat protein (Rathore *et al*., 2017). Gossypol‐free cottonseed meal can be a new source of protein for the more efficient aquaculture species and poultry, or can even be used as human food. The identification of the three *CGF* genes that play a direct or indirect role in gland formation provides us with the tools to suppress gland formation by silencing any one or more of these genes. Thus, strict tissue‐specific silencing of the *CGF* gene(s) in the seed kernel should eliminate or significantly reduce its gossypol content. Tissue‐specific silencing of a gene represents a powerful approach to examine the effects of silencing a gene in a particular tissue, and the trait created by these methods is stable and heritable (Houmard *et al*., 2007; Liu *et al*., 2002; Palle *et al*., 2013; Rathore *et al*., 2012; Schmidt *et al*., 2011; Sunilkumar *et al*., 2006). Strict tissue specificity of such gene silencing is critical because the terpenoid contents of the glands in the rest of the cotton plant provide protection against various pests and pathogens (Hedin *et al*., 1992b; Stipanovic *et al*., 1999). The expression profile of the three *CGF* genes in the embryos of glanded and glandless cotton at various stages of development suggests that *CGF2* and *GoPGF* (synonym *CGF3*) can be safely targeted for silencing as these two genes are not transcribed in the embryos of glandless cotton and thus not necessary for normal embryo development (Figure 3). To ensure complete elimination of gossypol from the cottonseed, it may be advisable to target one of these two *CGF* genes in combination with δ‐cadinene synthase gene for silencing. Silencing of δ‐cadinene synthase, that catalyses a key step in the biosynthesis of gossypol, has been used successfully to significantly reduce gossypol in the cottonseed by 98% (Sunilkumar *et al*., 2006). The combined targeting of two different types of genes should ensure complete elimination of seed gossypol in case some glands do develop despite silencing of *CGF2* and *GoPGF* (synonym *CGF3*) genes. There are several gene‐silencing technologies available such as RNAi (Hebert *et al*., 2008; Smith *et al*., 2000; Sunilkumar *et al*., 2006), CRISPR interference (CRISPRi) (Larson *et al*., 2013; Zhao *et al*., 2014) and C2c2 (CRISPR‐Cas13a)‐mediated destruction of specific transcripts (Abudayyeh *et al*., 2016). Any such gene silencing technologies in conjunction with a seed‐specific promoter can be used to eliminate the glands and thus gossypol from the cottonseed only. While elimination or significant reduction in gossypol from the cottonseed is a highly desirable goal, its commercial success may require modification of cultivation and especially seed storage practices to address the possibility of increased predation, especially by rodents.

While the CaMV35S promoter is typically considered to be too strong to drive a gene encoding a regulatory protein, our results on the callus cultures overexpressing the *ACGF3* gene point to an intriguing possibility of increasing the number of glands in the foliage and floral tissue by driving the expression of this gene under the control of its own promoter or another suitable promoter. It is also possible to increase the expression of the *GoPGF* (synonym *CGF3*) genes using some form of CRISPR/Cas9 technology to enhance the activity of the respective native promoters. In this regard, it will be important to understand the molecular basis underlying higher gland density in some of the cotton genotypes. Thus, we believe that seed‐specific silencing of *CGF2/CGF3* genes and/or δ‐cadinene synthase genes, whereas overexpressing the *CGF3* gene in other organs, by modification of native promoters or transgenic overexpression, can provide a cotton plant that produces gossypol‐free seeds, while having greater number of glands (and therefore higher levels of gossypol and related terpenoids) in rest of the plant for more robust defence against pests and pathogens, albeit at a slightly higher metabolic cost. There is an increasing need for such a ‘natural’ defence mechanism against pests because more and more insect species are developing resistance to various forms of Bt‐cotton. The cost of refining oil from such gossypol‐free cottonseed will be lower, and the meal can be used as a source of protein for the more efficient monogastric animals (poultry, swine and aquaculture species) and even as food, thus enhancing nutrition security in the cotton‐producing parts of the world.

## Experimental procedures

### Plant materials, RNA isolation, library preparation and RNA‐sequencing

Near‐isogenic lines of tetraploid cotton (*Gossypium hirsutum* L.) cultivar Stoneville 7A, designated Stoneville 7A glanded (STV GL; GVS4; *Gl*_*2*_ *Gl*_*2*_ *Gl*_*3*_ *Gl*_*3*_) and Stoneville 7A glandless (STV gl; GVS5; *gl*_*2*_ *gl*_*2*_ *gl*_*3*_ *gl*_*3*_) (Scheffler and Romano, 2012) were used for comparative RNA‐seq analysis to identify the genes that are involved in gland formation. Fully opened flowers were tagged on the greenhouse‐grown plants of GVS4 and GVS5. Bolls at 14‐, 16‐, 32‐dpa were collected and embryos were carefully dissected from the developing seeds under a stereo‐microscope. A glanded cultivar, Coker 312 was used to conduct VIGS and CRISPR/Cas9 experiments to validate the function of the candidate genes.

Total RNA was extracted from three independent biological replicates of each embryo sample (100–200 mg) using the Spectrum Plant Total RNA Kit (Sigma‐Aldrich, St. Louis, MO) following manufacturer's instructions. After on‐column DNase I treatment to remove the DNA from samples, RNA was eluted with nuclease‐free water. RNA quantity was measured using micro spectrophotometer (Nano‐Drop Technologies, Inc., Thermo Fisher Scientific Inc., Waltham, MA), and its quality was assessed with Agilent 2100 Bioanalyzer (Agilent Technologies, Inc., Santa Clara, CA). Only the samples with RNA integrity number (RIN) above 8.0 were used for the analysis.

Library preparation and RNA‐seq were performed by Texas A&M AgriLife Genomics and Bioinformatics Services. Poly‐A enriched mRNA from each replicate sample was used for the library preparation, 125‐bp paired‐end sequencing was performed using Illumina HiSeq 2500. Sequence cluster identification, quality pre‐filtering, base calling and uncertainty assessment were done in real time using Illumina HCS 2.2.58 and RTA 1.18.64 software (Illumina Inc., San Diego, CA) with default parameter settings.

### Bioinformatics analysis

RNA‐seq data were further processed using Trimmomatic software to filter out the low‐quality reads (Bolger *et al*., 2014) using LEADING:20 TRAILING:20 SLIDINGWINDOW:5:20 MINLEN:100 as parameters. Filtered reads were then mapped to the *G. hirsutum* (Texas Marker‐1) reference genome (Zhang *et al*., 2015) using HISAT2 program (Kim *et al*., 2015) and gene annotation in GFF3 format (NBI_Gossypium_hirsutum_v1.1.gene.gff3) (Yu *et al*., 2014). The allotetraploid cotton *G. hirsutum* L. acc. Texas Marker‐1 (TM‐1) is widely used as a genetic standard and its genome was sequenced in 2015 (Zhang *et al*., 2015). The output from the HISAT2 program was then analysed to quantify the reads per gene using the HTSeq‐count program (Anders *et al*., 2015). The differentially expressed genes were identified using DESeq2 (Love *et al*., 2014). The False Discovery Rate was set to ≤0.05 and the log fold change value to ≥2 to determine differentially expressed genes.

### CRISPR/Cas9‐mediated knockout of *CGF2* and *GoPGF* (synonym *CGF3*) genes

*CGF2* and *GoPGF* (synonym *CGF3*) genes were targeted for knockout using the CRISPR/Cas9 system. The guide sequences used to target *CGF2* and *GoPGF* (synonym *CGF3*) are listed in Table S5. Selected lines showing the mutant phenotype (absence of glands or malformed glands) were analysed by sequencing the amplicon, encompassing the target sites, generated from the genomic DNA isolated from the leaves of T0 plants. Primer sequences are provided in Table S6. A complete description of the CRISPR/Cas9‐mediated knockout and mutation analysis is provided in the Supplementary Methods.

A detailed description of procedures, including VIGS, terpenoid analysis, qRT‐PCR, sequencing, promoter activity assay, etc. is provided in Supplementary Methods (Data S2).

## Supporting information


**Figure S1** Microscopic images of developing embryos of Stoneville 7A glanded (GL; GVS4) and glandless (gl; GVS5), near‐isogenic lines, used for comparative RNA‐seq analysis.
**Figure S2** Virus‐induced gene silencing (VIGS) in cotton.
**Figure S3** Leaves from plants that had undergone virus‐induced gene silencing against *CGF* genes showing the effects on gland formation.
**Figure S4** Figure S4 Real‐time PCR standard curves representing PCR efficiency for each of the CGF homeologs and Histone gene.
**Figure S5** Alignment of A subgenome *CGF1* gene sequences from GVS4 (glanded) and GVS5 (glandless, recessive mutant) near‐isogenic lines.
**Figure S6** Alignment of D subgenome *CGF1* gene sequences from GVS4 (glanded) and GVS5 (glandless, recessive mutant) near‐isogenic lines.
**Figure S7** Alignment of A subgenome *CGF2* gene sequences from GVS4 (glanded) and GVS5 (glandless, recessive mutant) near‐isogenic lines.
**Figure S8** Alignment of D subgenome *CGF2* gene sequences from GVS4 (glanded) and GVS5 (glandless, recessive mutant) near‐isogenic lines.
**Figure S9** Alignment of A subgenome *GoPGF* (synonym *CGF3*) gene sequences from GVS4 (glanded) and GVS5 (glandless, recessive mutant) near‐isogenic lines.
**Figure S10** Alignment of D subgenome *GoPGF* (synonym *CGF3*) gene sequences from GVS4 (glanded) and GVS5 (glandless, recessive mutant) near‐isogenic lines.
**Figure S11** Sequencing results showing four different SNPs that differentiate A and D subgenome *GoPGF* (synonym *CGF3*) genes.
**Figure S12** Promoter (2.05 kb) activity evaluation of the D subgenome *GoPGF* (synonym *CGF3*) gene from glanded and glandless cotton using gusA as the reporter gene.
**Figure S13** Promoter (~4.2 kb) activity evaluation of the D subgenome *GoPGF* (synonym *CGF3*) gene from glanded and glandless cotton using gusA as the reporter gene.
**Figure S14** Effect of CRISPR/Cas9‐mediated knockout of *CGF2* and *GoPGF* (synonym *CGF3*) genes on gland formation in T0 plants in comparison to glanding pattern seen in a wild‐type, control cotton plant.
**Figure S15** Mutations observed in two *CGF2* knockout lines, (a) 236‐8 and (b) 236‐10. The two target sites are highlighted yellow and PAM sequences red.
**Figure S16** Mutations observed in two *GoPGF* (synonym *CGF3*) knockout lines, (a) 237‐3 and (b) 237‐4.
**Figure S17** Effect of CRISPR/Cas9‐mediated knockout of *CGF2* and *GoPGF* (synonym *CGF3*) genes on gland formation observed in cottonseed kernels.
**Figure S18** qRT‐PCR analysis of *GoPGF* (synonym *CGF3*) transcripts in cotton callus cultures obtained following transformation with *ACGF3* overexpression construct. L254: light‐coloured callus lines; D254 dark‐coloured callus lines; Control: nontransgenic callus.
**Table S1** RNA‐seq reads for glanded (GL; GVS4) and glandless (gl; GVS5) embryos at 14‐, 16‐ and 32‐days post‐anthesis and their mapping to the reference genome.
**Table S2** Genes that expressed at higher levels in the glanded embryos (STV GL; GVS4; Gl2Gl2Gl3Gl3) in comparison to those in the glandless (STV gl; GVS5; gl2gl2gl3gl3) embryos at 14‐days post‐anthesis stage of development based on RNA‐seq analysis. Genes encoding putative transcription factors were tested for their role in gland formation using virus‐induced gene silencing (VIGS).
**Table S3** Primers used to amplify segments of the coding sequence of the target gene for cloning into TRV2 binary vector to conduct VIGS experiments.
**Table S4** Primers used to amplify and isolate *CGF* genes from A and D subgenomes of glanded (GVS4) and glandless (GVS5) cotton plants.
**Table S5** Guide sequences used to target *CGF2* and *GoPGF* (synonym *CGF3*) genes.
**Table S6** Primers used for amplicon sequencing of regenerated plants targeted with LCT236, LCT237 and LCT238 constructs.

**Data S1** Differentially expressed genes between glanded and glandless embryos at 16‐ and 32 days post anthesis.

**Data S2** Supplementary methods.

## Data Availability

RNA‐seq data from developing embryos at different stages of development (14‐, 16‐ and 32‐dpa) from both glanded (GVS4), and glandless (GVS5) cotton are available in the NCBI SRA archive (accession # PRJNA448612).
