# Supplementary material for: Genes regulating gland development in the cotton plant
Source: Plant Biotechnol J. 2018 Dec 21;17(6):1142–53. doi: 10.1111/pbi.13044 (PMC6523598; doi:10.1111/pbi.13044)
Supplement: Supplementary file 1 — Figure S1 Microscopic images of developing embryos of Stoneville 7A glanded (GL; GVS4) and glandless (gl; GVS5), near‐isogenic lines, used for comparative RNA‐seq analysis. Figure S2 Virus‐induced gene silencing (VIGS) in cotton. Figure S3 Leaves from plants that had undergone virus‐induced gene silencing against CGF genes showing the effects on gland formation. Figure S4 Figure S4 Real‐time PCR standard curves representing PCR efficiency for each of the CGF homeologs and Histone gene. Figure S5 Alignment of A subgenome CGF1 gene sequences from GVS4 (glanded) and GVS5 (glandless, recessive mutant) near‐isogenic lines. Figure S6 Alignment of D subgenome CGF1 gene sequences from GVS4 (glanded) and GVS5 (glandless, recessive mutant) near‐isogenic lines. Figure S7 Alignment of A subgenome CGF2 gene sequences from GVS4 (glanded) and GVS5 (glandless, recessive mutant) near‐isogenic lines. Figure S8 Alignment of D subgenome CGF2 gene sequences from GVS4 (glanded) and GVS5 (glandless, recessive mutant) near‐isogenic lines. Figure S9 Alignment of A subgenome GoPGF (synonym CGF3) gene sequences from GVS4 (glanded) and GVS5 (glandless, recessive mutant) near‐isogenic lines. Figure S10 Alignment of D subgenome GoPGF (synonym CGF3) gene sequences from GVS4 (glanded) and GVS5 (glandless, recessive mutant) near‐isogenic lines. Figure S11 Sequencing results showing four different SNPs that differentiate A and D subgenome GoPGF (synonym CGF3) genes. Figure S12 Promoter (2.05 kb) activity evaluation of the D subgenome GoPGF (synonym CGF3) gene from glanded and glandless cotton using gusA as the reporter gene. Figure S13 Promoter (~4.2 kb) activity evaluation of the D subgenome GoPGF (synonym CGF3) gene from glanded and glandless cotton using gusA as the reporter gene. Figure S14 Effect of CRISPR/Cas9‐mediated knockout of CGF2 and GoPGF (synonym CGF3) genes on gland formation in T0 plants in comparison to glanding pattern seen in a wild‐type, control cotton plant. Figure S15 Mutations [file PBI-17-1142-s002.pdf]

Genes regulating gland development in the cotton plant

Madhusudhana R. Janga[1], Devendra Pandeya[1], LeAnne M. Campbell[1], Kranti Konganti[2], Stephany Toinga Villafuerte[1], Lorraine Puckhaber[3], Alan Pepper[4], Robert D. Stipanovic[3], Jodi A. Scheffler[5], Keerti S. Rathore[1,6*]

[1]Institute for Plant Genomics & Biotechnology, Texas A&M University, College Station, TX, 77843-2123, USA; [2]Texas A&M Institute for Genome Sciences and Society, Texas A&M University, College Station, TX, 77843; [3]Southern Plains Agricultural Research Center, USDA-ARS, College Station, TX, USA; [4]Dept. of Biology, Texas A&M University, College Station, TX, 77843; [5]Crop Genetics Research Unit, USDA-ARS, Stoneville, MS, USA; [6]Dept. of Soil & Crop Sciences, Texas A&M University, College Station, TX, 77843-2474, USA.

[*]Corresponding author: Keerti S. Rathore
Institute for Plant Genomics & Biotechnology
Dept. of Soil & Crop Sciences
Texas A&M University
College Station, TX 77843-2123, USA
Email: rathore@tamu.edu
Phone: (979) 862 4795
Fax: (979) 862 3414

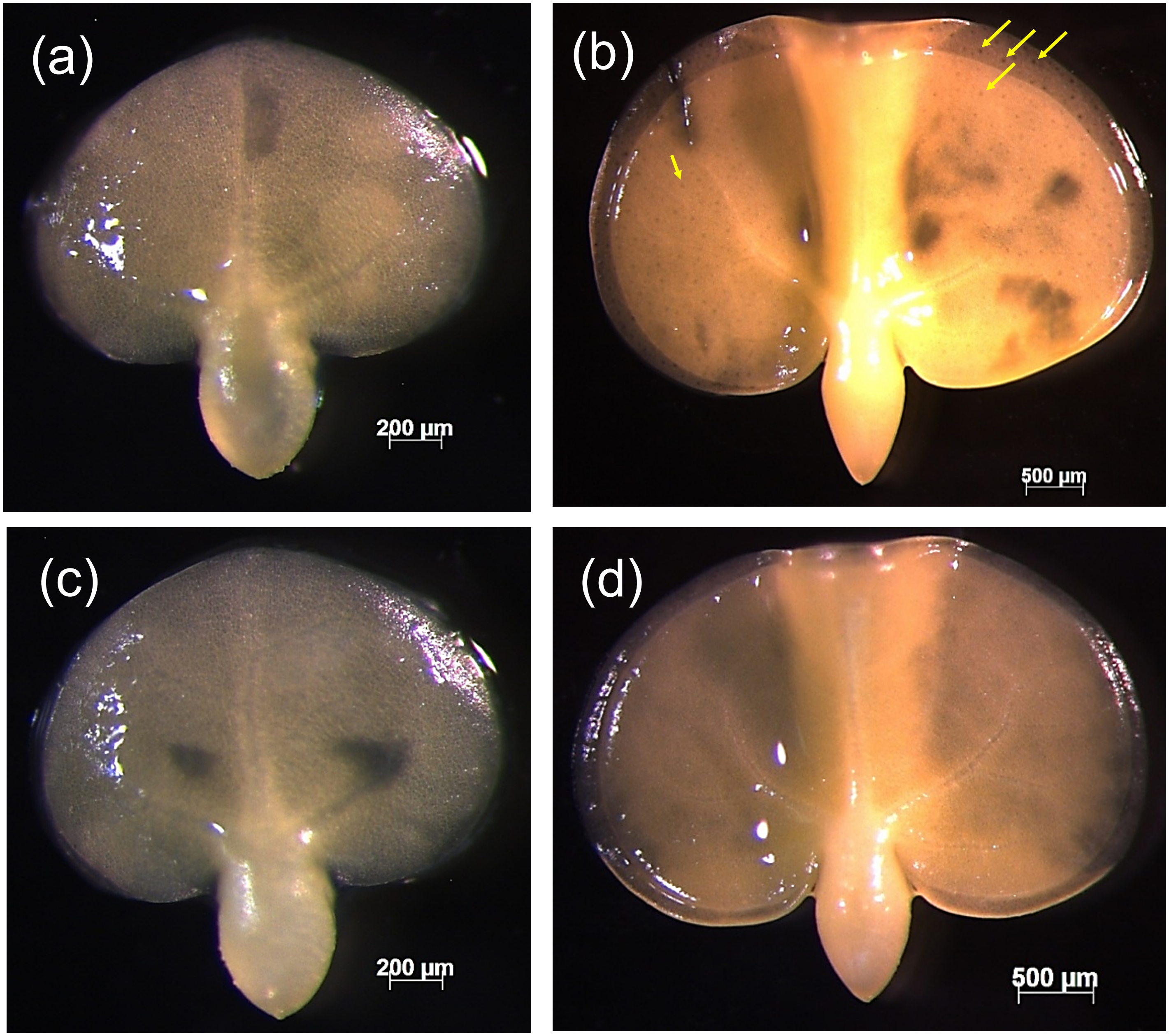

Figure S1 Microscopic images of developing embryos of Stoneville 7A glanded (GL; GVS4) and glandless (gl; GVS5), near-isogenic lines, used for comparative RNA-seq analysis. (**a**) 14-days post-anthesis (dpa) glanded embryo; (**b**) 16-dpa glanded embryo, the arrows indicate glands that are visible microscopically; (**c**) 14-dpa glandless embryo; (**d**) 16-dpa glandless embryo.

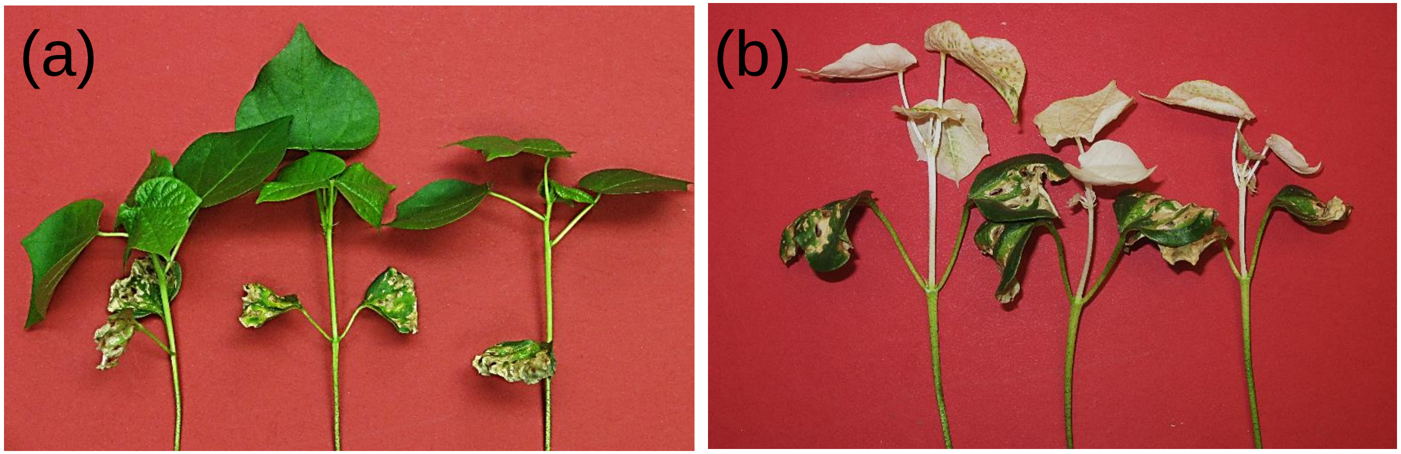

Figure S2 Virus Induced Gene Silencing (VIGS) in cotton. (**a**) empty vector (RNA1 and RNA2) infiltrated plants. (**b**) plants subjected to VIGS-mediated silencing of *GhCLA* gene. The leaves showing albino phenotype indicate the efficacy of the VIGS treatment. The photographs were taken four weeks after infiltration.

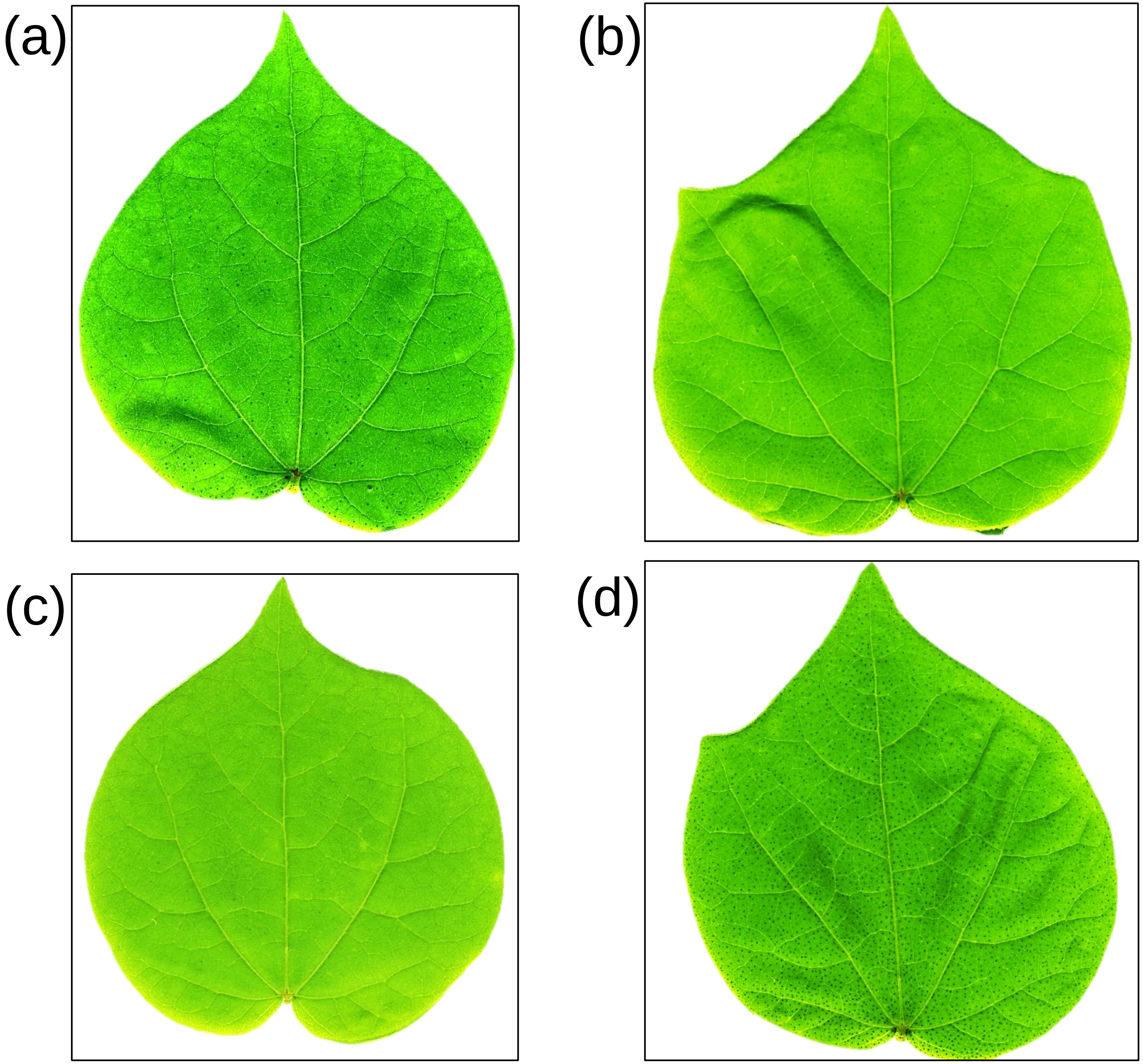

Figure S3 Leaves from plants that had undergone Virus-induced Gene Silencing against *CGF* genes showing the effects on gland formation. (**a**) silencing of *CGF1* gene, (**b**) silencing of *CGF2* gene, (**c**) silencing of *GoPGF* (synonym *CGF3*) gene, (**d**) empty vector control.

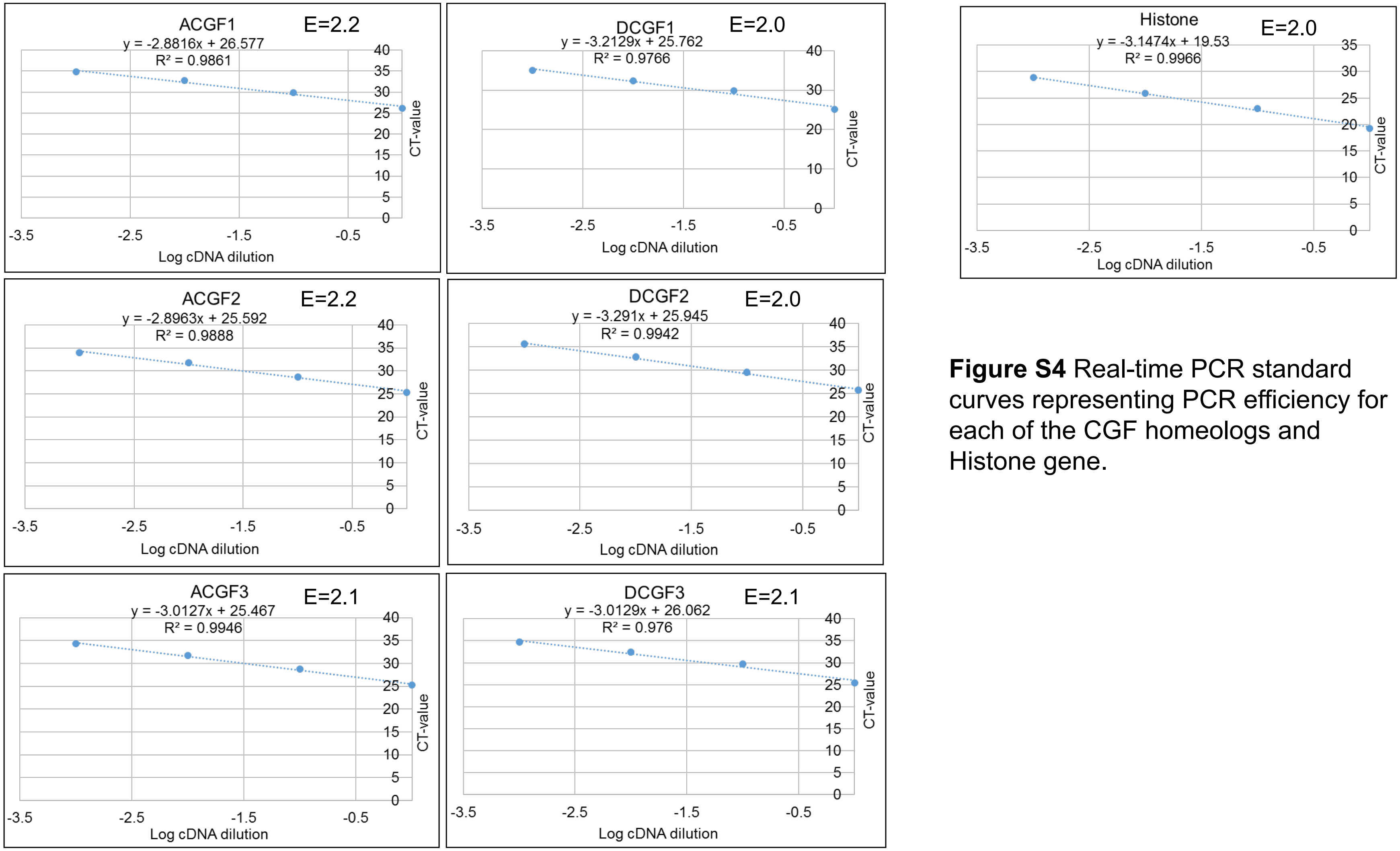

Figure S4 Real-time PCR standard curves representing PCR efficiency for each of the CGF homeologs and Histone gene.

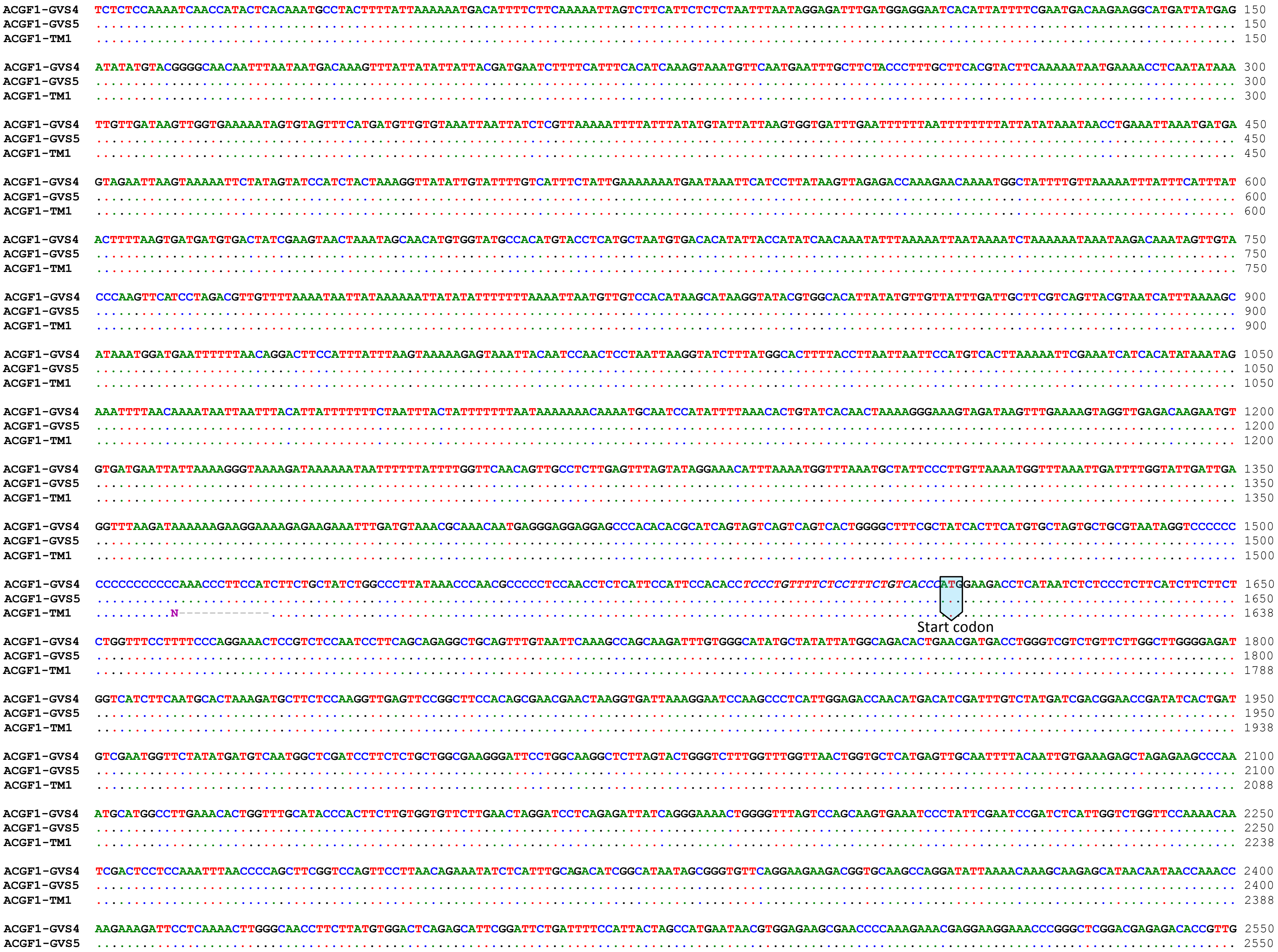
ACGF1-GVS4
ACGF1-GVS5
ACGF1-TM1
Start codon

```
ACGF1-TM1    ...................................................................................................................................................... 2538

ACGF1-GVS4   AATCATGTGGAAGCGGAGAGGCAACGCCGTGAGAAGCTGAACCACAGATTCTACGCTCTCCGAGCAGTAGTCCCGAACGTGTCGCGCATGGACAAAGCATCGCTCTTATCTGACGCTGTTTCCTACATCAATGATCTAAAGGCGAAAATT 2700
ACGF1-GVS5   ...................................................................................................................................................... 2700
ACGF1-TM1    ...................................................................................................................................................... 2688

ACGF1-GVS4   GATGAATTAGAGTCGCAGCTTCAGAGAGAGTGTAAGAAAGTGAAGGTAGAAATGGTTGATACAATGGATAACCAAAGCACCACTACTACTACCACATCTGAGGAAGAGCAACAGGCAGCCAGGCCCAGCTATTCATCTCCTGGAACTGGG 2850
ACGF1-GVS5   ...................................................................................................................................................... 2850
ACGF1-TM1    ...................................................................................................................................................... 2838

ACGF1-GVS4   AGTGGCATTGAACTGGAGGTCAAGATTATGGTAAATGATGCAATGATAAGGGTTCACTCAGAGAATGTGAACTATCCAGCAGCTAGGCTAATGGGTGCCCTTCGTGACTTGGAATTCCAGGTCCGTCATGCAAGCATGTCGTCTGTTAAC 3000
ACGF1-GVS5   ...................................................................................................................................................... 3000
ACGF1-TM1    ...................................................................................................................................................... 2988

ACGF1-GVS4   GACCTCATGCTTCAAGACATAGTGGTCAGGCTTCCTGATGGGTTGAGAACTGAAGAAGGCCTCAAATCTGCTCTCCTCAGGAGGCTAGATCTGCAGTAAGGTTTTATTATGGTTATCCAGAGTTTGTATTTTAAAATGCTCTATTTCCTC 3150
ACGF1-GVS5   ...................................................................................................................................................... 3150
ACGF1-TM1    ...................................................................................................................................................... 3138
                                                                                                       Stop codon

ACGF1-GVS4   CTACTTCTTTCAGTTTATTTTGTTTTAACTTTTGCTTCATCTTCAGGTTTGCTTTAGCCGTTTCTTTGCTTTTCTGCTTGTTACTATGTAACTCTTTGGGAGAGGGTTCTGCACTGCTTTCTTTTATCAAATGTTCTATATTTATTCTAG 3300
ACGF1-GVS5   ...................................................................................................................................................... 3300
ACGF1-TM1    ...................................................................................................................................................... 3288

ACGF1-GVS4   TGTCTGTTCCCCGAGTTAAGGACAATAATATTCCTTAATGGATTATACACATACAAAAAAGAACGGAAACAATATTCGTGCATGTTTATCAACTAGAAACCAAATCCATGCCAAAAACATGTATAATAATGTCTGCCCCTATTTAAGGAC 3450
ACGF1-GVS5   ...................................................................................................................................................... 3450
ACGF1-TM1    ...................................................................................................................................................... 3438

ACGF1-GVS4   TCAAATATACATATTTT 3467
ACGF1-GVS5   ................. 3467
ACGF1-TM1    ................. 3455
```

Figure S5 Alignment of A subgenome *CGF1* gene sequences from GVS4 (glanded) and GVS5 (glandless, recessive mutant) near-isogenic lines. Gene sequence of Texas Marker-1 (TM-1, a widely used genetic standard) is also provided for comparison purpose. The ~3.47 kb sequence of *A CGF1* representing the promoter region (~1.59 kb), 5'-UTR (italics), coding region, 3'-UTR (italics), and the terminator is identical between GVS4 and GVS5. Translation initiation codon and stop codon are indicated.

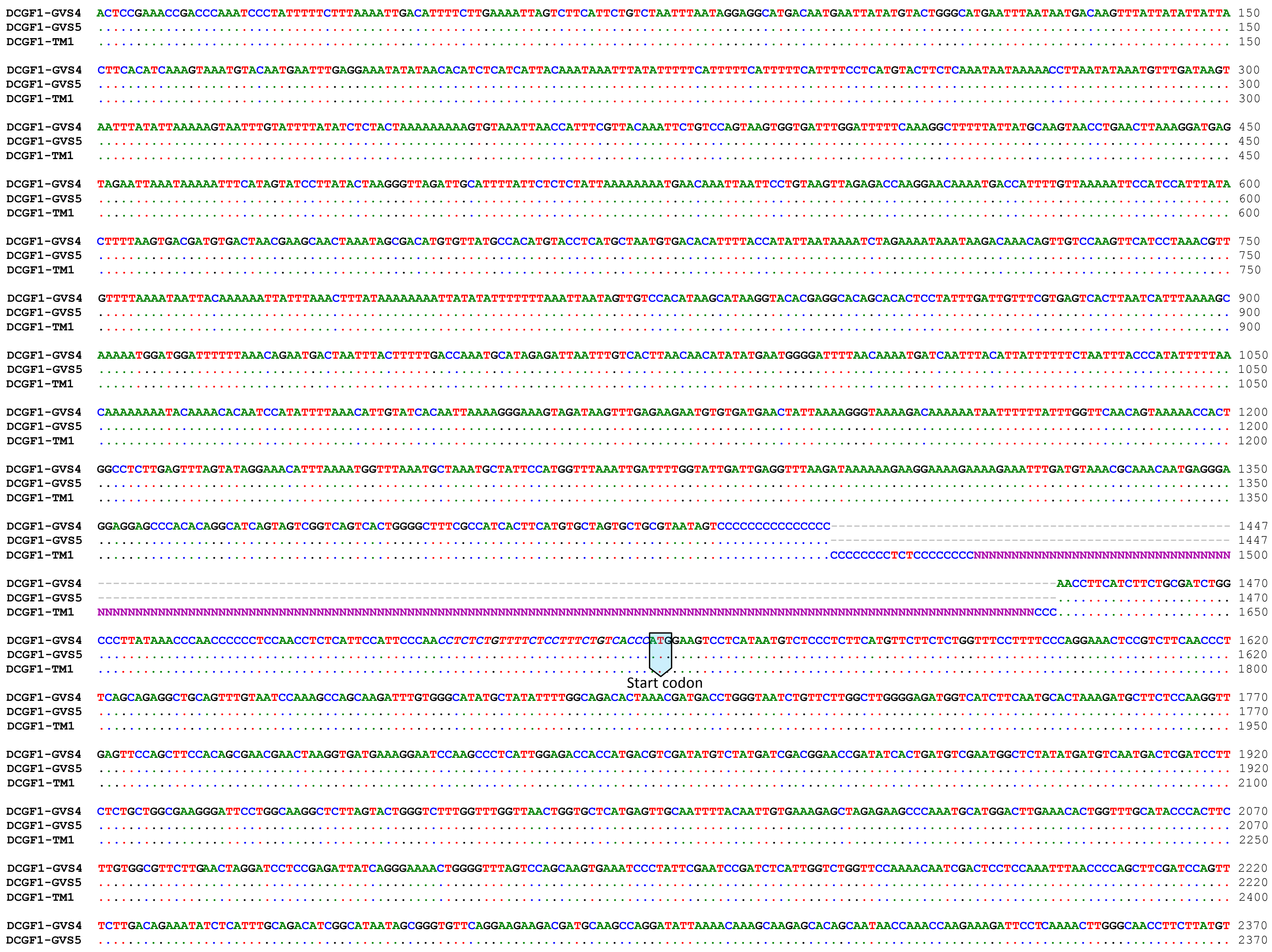
DCGF1-GVS4
DCGF1-GVS5
DCGF1-TM1
Start codon

DCGF1-TM1 2550

DCGF1-GVS4 GGACTCAGAGCATTCAGATTCTGATTTTCCATTACTAGCCATGAATAACGTGGAGAAGCGAACCCCAAAGAAACGAGGAAGGAAACCCGGCCTCGGACGAGAGACACCGTTGAACCATGTGGAAGCGGAGAGGCAACGCCGTGAGAAGCT 2520
DCGF1-GVS5 2520
DCGF1-TM1 2700

DCGF1-GVS4 GAACCACAGATTCTACGCTCTCCGAGCAGTAGTCCCGAACGTGTCGCGCATGGACAAAGCATCGCTCTTATCTGACGCTGTTTCCTACATCAATGATCTAAAGGCGAAAATTGATGAATTAGAGTCGCAGCTTCAGAGAGAGCGTAAGAA 2670
DCGF1-GVS5 2670
DCGF1-TM1 2850

DCGF1-GVS4 AGTGAAGGTAGAAATGGTTGATACAATGGATAACCAAAGCACCACTACTACTACCACATCTGAGGAAGAGCAACAGGCAACCAGGCCCAGCTATTCATCTCCTGGAACTGGGAGTGGCATTGAACTGGAGGTCAAGATTATGGTAAATGA 2820
DCGF1-GVS5 2820
DCGF1-TM1 3000

DCGF1-GVS4 TGCAATGATTAGGGTTCACTCAGAGAATGTGAACTATCCAGCAGCTAGGCTAATGGGTGCCCTTCGTGACTTGGAATTCCAGGTCCATCATGCAAGCATGTCGTCTGTTAACGACCTCATGCTTCAGGACATTGTGGTCAGGCTTCCTGA 2970
DCGF1-GVS5 2970
DCGF1-TM1 3150

DCGF1-GVS4 TGGGTTGAGAACTGAAGAAGGCCTCAAATCTGCTCTTCTCAGGAGGCTAGATCTGCAGTAA*GGTTTAATTATGGTTATCCAGAGTTTGTATTTTAAAATGCTCTATTTCCTCCTACTTATTTTTATCCCTTCCGTTTCTTTCTTCTTTCA* 3120
DCGF1-GVS5 3120
DCGF1-TM1 3300

Stop codon

DCGF1-GVS4 *GTTTATTTTGTTTTAACTTTTGCTTCATCTTCAGGTTTGCTTTAGCCGTTTCTTTTCTTTTCTGCTTCTTACTATGTAACTCTTGGGGGAGGGGGTTCTGCGCTGCTTTCTTTTATCAAATGTTCTACATTTATTCTAGCGTCTGTTCCC* 3270
DCGF1-GVS5 3270
DCGF1-TM1 3450

DCGF1-GVS4 *CGAGTTCAGGAAAATCATATTCCTTAATGGATAT*ACACATACAAAAAAGAACGAAAACAATATTCGTGCATGTTTATCAGCTAGAAACAAAGTCCATGCCAAAAACATATATAATCATGTCTGCCCGTTATTAAGGACTCAAATATAGAT 3420
DCGF1-GVS5 3420
DCGF1-TM1 3600

DCGF1-GVS4 ATTTTTGTTCTTACATATCAAATCAGATTACTATTGATGAACCTCAAAATCTAGGACTTGAAGCCAACGGAGGATGCTATGGAGACGGGACAATCGTTGCGGAATTGTGCAAAACAGAGGACCGTATACCACTATTTACCACGTATAAAG 3570
DCGF1-GVS5 3570
DCGF1-TM1 3750

DCGF1-GVS4 AGAAGCTTATATATATGGTAATTGCTCAGGGATCAGTCCACGAAGAAAACCTATATCTTTTCTTGAAATGAAAAAGCAAAATAAAAAATTTGTGAGAAATATTTGGAAAAAGAAAATTTATAAGTTTTGGACGTTCCTAAATTTGGCTCT 3720
DCGF1-GVS5 3720
DCGF1-TM1 3900

DCGF1-GVS4 TAAAAATTTAATGATGACAAATCTCTACAAATTTTTTATATTACAAAAC 3769
DCGF1-GVS5 3769
DCGF1-TM1 3949

Figure S6 Alignment of D subgenome *CGF1* gene sequences from GVS4 (glanded) and GVS5 (glandless, recessive mutant) near-isogenic lines. Sequence of Texas Marker-1 (TM-1, a widely used genetic standard) is also provided for comparison purpose. The ~3.77 kb sequence of *D CGF1* representing the promoter region (~1.5 kb), 5'-UTR (italics), coding region, 3'-UTR (italics), and the terminator is identical between GVS4 and GVS5. Translation initiation codon and stop codon are indicated.

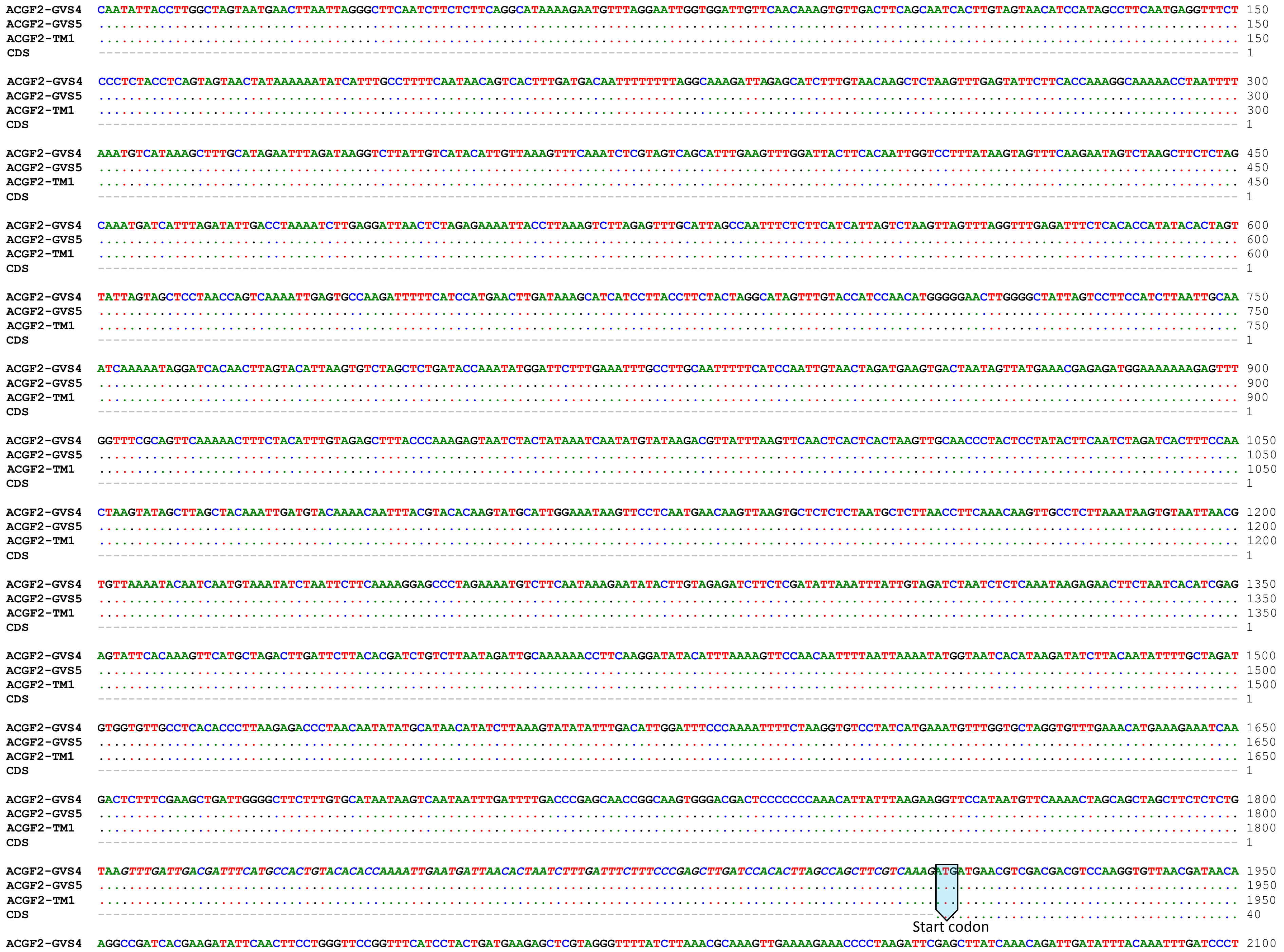
ACGF2-GVS4
ACGF2-GVS5
ACGF2-TM1
CDS
Start codon

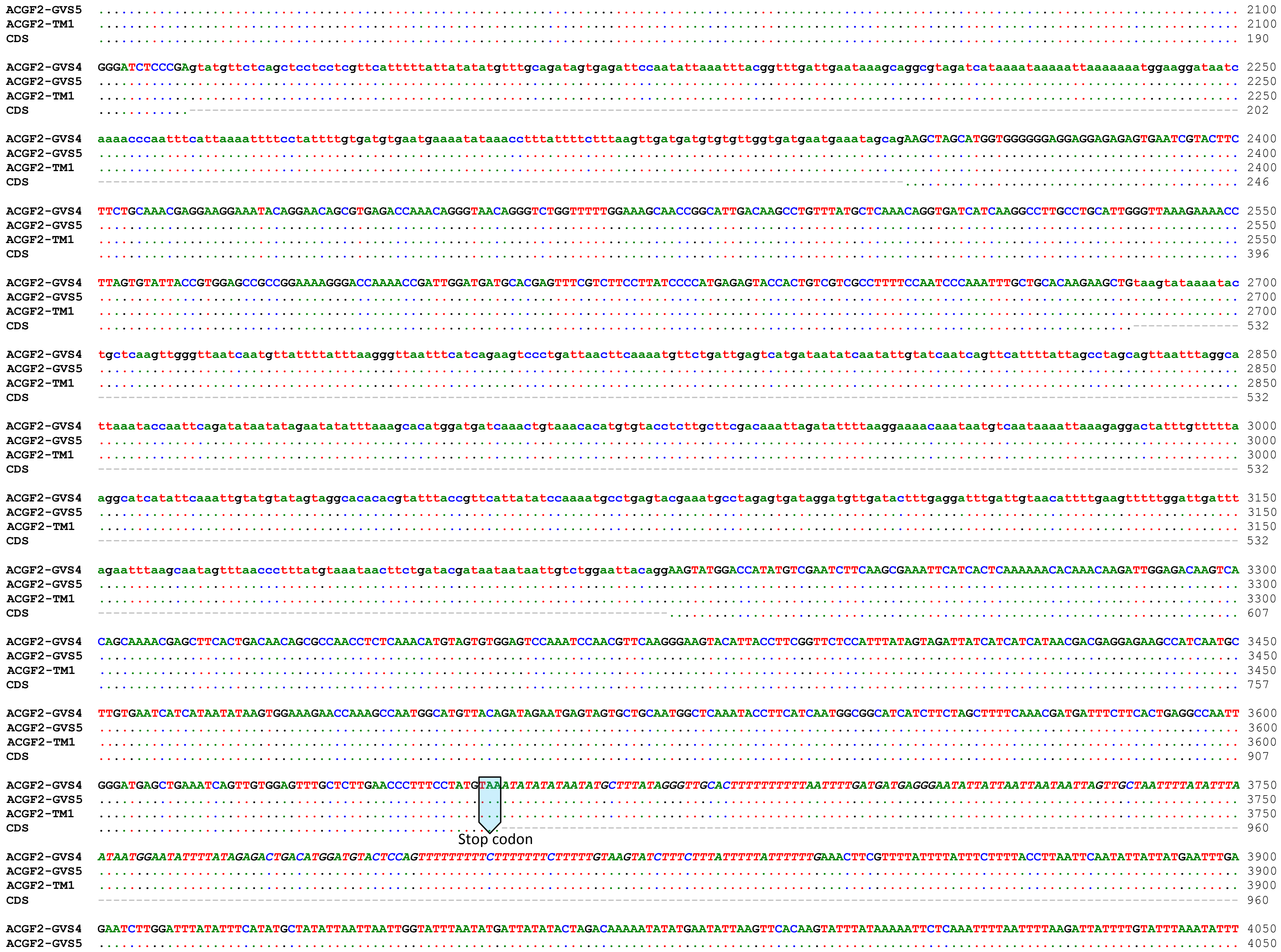
Stop codon

```
ACGF2-TM1    ...................................................................................................................................................... 4050
CDS          ------------------------------------------------------------------------------------------------------------------------------------------------------ 960

ACGF2-GVS4   TTTTAGAAATTTATGATTTTTAATTTTTTTATAGAAATTTATGATTCTTTTTATTGGAATTATAACAAAAATTTATTAATAATTTACTCAAAATGAAGTCATTATATATGGTTTTTTTTCTAATGGTGTATACTAAAATTTATTAATTAT 4200
ACGF2-GVS5   ...................................................................................................................................................... 4200
ACGF2-TM1    ...................................................................................................................................................... 4200
CDS          ------------------------------------------------------------------------------------------------------------------------------------------------------ 960

ACGF2-GVS4   GATTCTATTTTGCATTGATGGATGTAATTTCATCAAAATAATAATTATATATTTTTCTACAAAG 4264
ACGF2-GVS5   ................................................................ 4264
ACGF2-TM1    ................................................................ 4264
CDS          ---------------------------------------------------------------- 960
```

Figure S7 Alignment of A subgenome *CGF2* gene sequences from GVS4 (glanded) and GVS5 (glandless, recessive mutant) near-isogenic lines. Sequence of Texas Marker-1 (TM-1, a widely used genetic standard) is also provided for comparison purpose. The ~4.26 kb sequence of *A CGF2* representing the promoter region (~1.8 kb), 5'-UTR (italics), coding region (including two introns, shown in small case), 3'-UTR (italics), and the terminator is identical between GVS4 and GVS5. Translation initiation codon and stop codon are indicated.

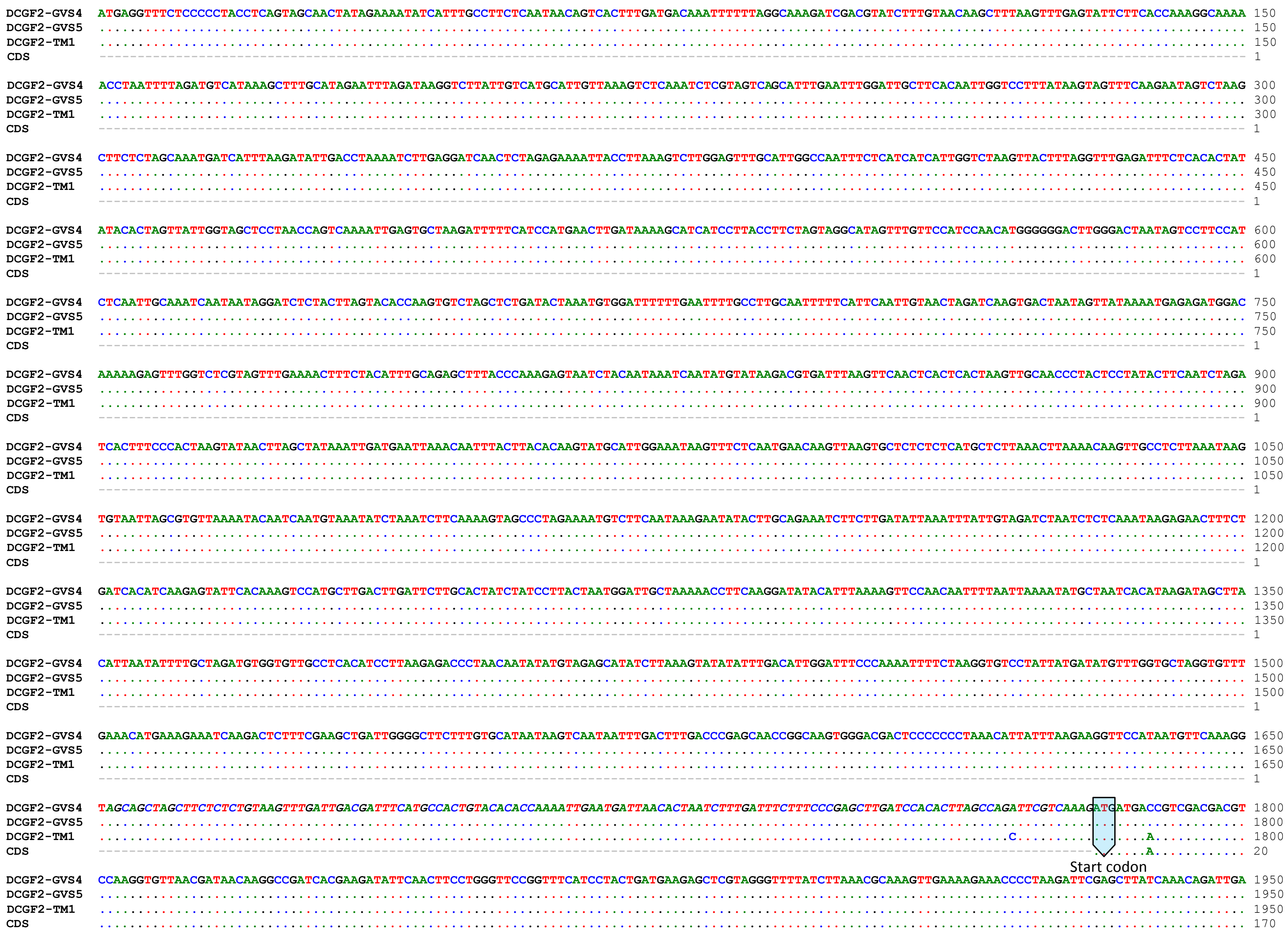
DCGF2-GVS4
DCGF2-GVS5
DCGF2-TM1
CDS
Start codon

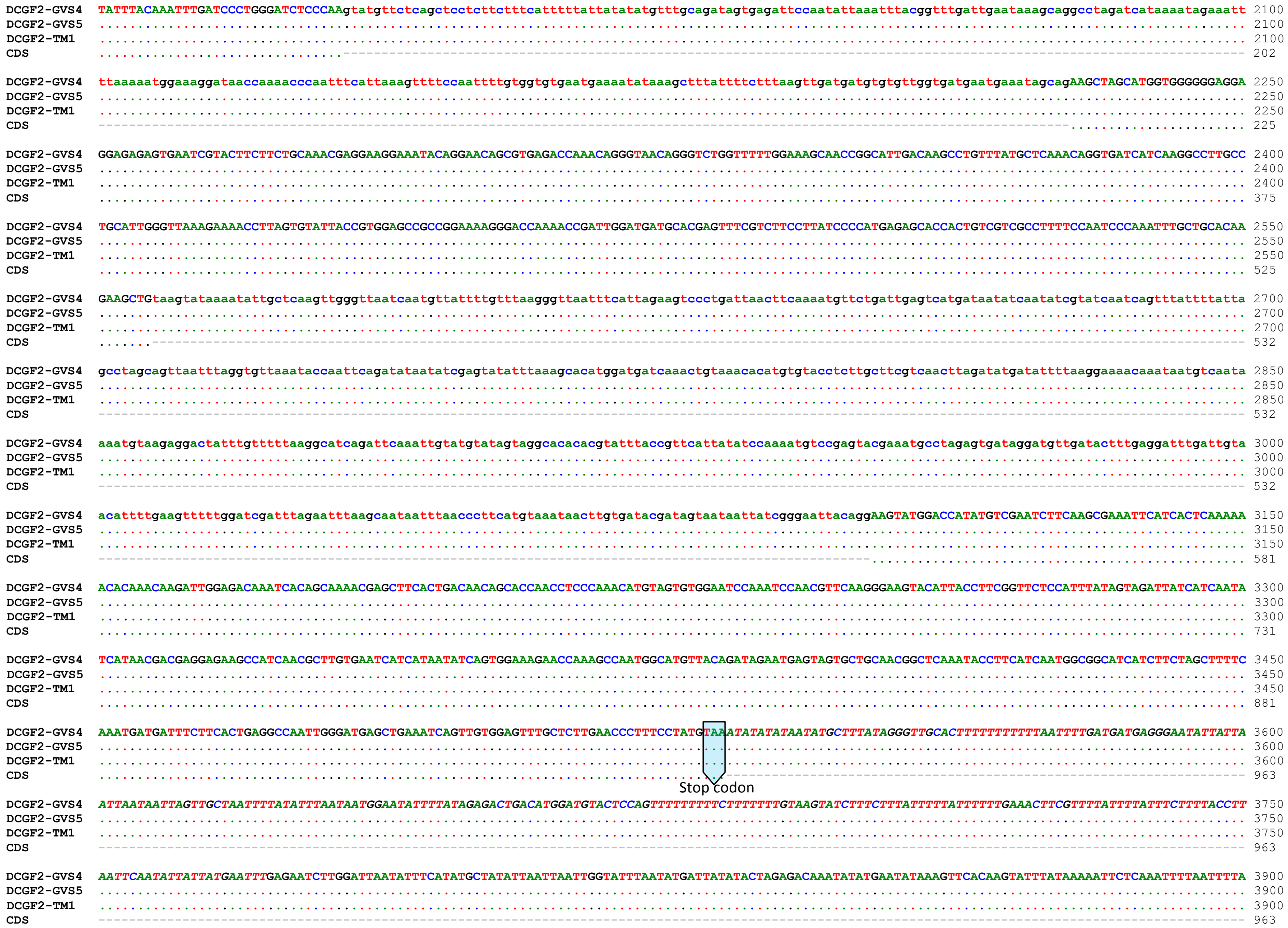

DCGF2-GVS4 TATTTACAAATTTGATCCCTGGGATCTCCCAAgtatgttctcagctcctcttctttcatttttattatatatgtttgcagatagtgagattccaatattaaatttacggtttgattgaataaagcaggcctagatcataaaatagaaatt 2100
DCGF2-GVS5 2100
DCGF2-TM1 2100
CDS 202
DCGF2-GVS4 ttaaaaatggaaaggataaccaaaacccaatttcattaaagttttccaattttgtggtgtgaatgaaaatataaagctttattttctttaagttgatgatgtgtgttggtgatgaatgaaatagcagAAGCTAGCATGGTGGGGGGAGGA 2250
DCGF2-GVS5 2250
DCGF2-TM1 2250
CDS 225
DCGF2-GVS4 GGAGAGAGTGAATCGTACTTCTTCTGCAAACGAGGAAGGAAATACAGGAACAGCGTGAGACCAAACAGGGTAACAGGGTCTGGTTTTTGGAAAGCAACCGGCATTGACAAGCCTGTTTATGCTCAAACAGGTGATCATCAAGGCCTTGCC 2400
DCGF2-GVS5 2400
DCGF2-TM1 2400
CDS 375
DCGF2-GVS4 TGCATTGGGTTAAAGAAAACCTTAGTGTATTACCGTGGAGCCGCCGGAAAAGGGACCAAAACCGATTGGATGATGCACGAGTTTCGTCTTCCTTATCCCCATGAGAGCACCACTGTCGTCGCCTTTTCCAATCCCAAATTTGCTGCACAA 2550
DCGF2-GVS5 2550
DCGF2-TM1 2550
CDS 525
DCGF2-GVS4 GAAGCTGtaagtataaaatattgctcaagttgggttaatcaatgttattttgtttaagggttaatttcattagaagtccctgattaacttcaaaatgttctgattgagtcatgataatatcaatatcgtatcaatcagtttattttatta 2700
DCGF2-GVS5 2700
DCGF2-TM1 2700
CDS 532
DCGF2-GVS4 gcctagcagttaatttaggtgttaaataccaattcagatataatatcgagtatatttaaagcacatggatgatcaaactgtaaacacatgtgtacctcttgcttcgtcaacttagatatgatattttaaggaaaacaaataatgtcaata 2850
DCGF2-GVS5 2850
DCGF2-TM1 2850
CDS 532
DCGF2-GVS4 aaatgtaagaggactatttgtttttaaggcatcagattcaaattgtatgtatagtaggcacacacgtatttaccgttcattatatccaaaatgtccgagtacgaaatgcctagagtgataggatgttgatactttgaggatttgattgta 3000
DCGF2-GVS5 3000
DCGF2-TM1 3000
CDS 532
DCGF2-GVS4 acattttgaagtttttggatcgatttagaatttaagcaataatttaacccttcatgtaaataacttgtgatacgatagtaataattatcgggaattacaggAAGTATGGACCATATGTCGAATCTTCAAGCGAAATTCATCACTCAAAAA 3150
DCGF2-GVS5 3150
DCGF2-TM1 3150
CDS 581
DCGF2-GVS4 ACACAAACAAGATTGGAGACAAATCACAGCAAAACGAGCTTCACTGACAACAGCACCAACCTCCCAAACATGTAGTGTGGAATCCAAATCCAACGTTCAAGGGAAGTACATTACCTTCGGTTCTCCATTTATAGTAGATTATCATCAATA 3300
DCGF2-GVS5 3300
DCGF2-TM1 3300
CDS 731
DCGF2-GVS4 TCATAACGACGAGGAGAAGCCATCAACGCTTGTGAATCATCATAATATCAGTGGAAAGAACCAAAGCCAATGGCATGTTACAGATAGAATGAGTAGTGCTGCAACGGCTCAAATACCTTCATCAATGGCGGCATCATCTTCTAGCTTTTC 3450
DCGF2-GVS5 3450
DCGF2-TM1 3450
CDS 881
DCGF2-GVS4 AAATGATGATTTCTTCACTGAGGCCAATTGGGATGAGCTGAAATCAGTTGTGGAGTTTGCTCTTGAACCCTTTCCTATGTAAATATATATATAATATGCTTTATAGGGTTGCACTTTTTTTTTTTAATTTTGATGATGAGGGAATATTATTA 3600
DCGF2-GVS5 3600
DCGF2-TM1 3600
CDS 963
Stop codon
DCGF2-GVS4 ATTAATAATTAGTTGCTAATTTTATATTTAATAATGGAATATTTTATAGAGACTGACATGGATGTACTCCAGTTTTTTTTTCTTTTTTTGTAAGTATCTTTCTTTATTTTTATTTTTTGAAACTTCGTTTTATTTTATTTCTTTTACCTT 3750
DCGF2-GVS5 3750
DCGF2-TM1 3750
CDS 963
DCGF2-GVS4 AATTCAATATTATTATGAATTTGAGAATCTTGGATTAATATTTCATATGCTATATTAATTAATTGGTATTTAATATGATTATATACTAGAGACAAATATATGAATATAAAGTTCACAAGTATTTATAAAAATTCTCAAATTTTAATTTTA 3900
DCGF2-GVS5 3900
DCGF2-TM1 3900
CDS 963

```
DCGF2-GVS4   AGATTCTTTTGTATTTCAATATTTTTTTTAGAATTTTATGATTTTTAGTTTTTTTTTATAGAAATTTATGATTCTTTTTATTGAAATTATAATGATACGTAACATACGAAAACCATTTTGTTACG 4023
DCGF2-GVS5   ............................................................................................................................. 4023
DCGF2-TM1    ............................................................................................................................. 4023
CDS          ----------------------------------------------------------------------------------------------------------------------------- 963
```

Figure S8 Alignment of D subgenome *CGF2* gene sequences from GVS4 (glanded) and GVS5 (glandless, recessive mutant) near-isogenic lines. Sequence of Texas Marker-1 (TM-1, a widely used genetic standard) is also provided for comparison purpose. The ~4.02 kb sequence of *D CGF2* representing the promoter region (~1.65 kb), 5'-UTR (italics), coding region (including two introns, shown in small case), 3'-UTR (italics), and the terminator is identical between GVS4 and GVS5. Translation initiation codon and stop codon are indicated.

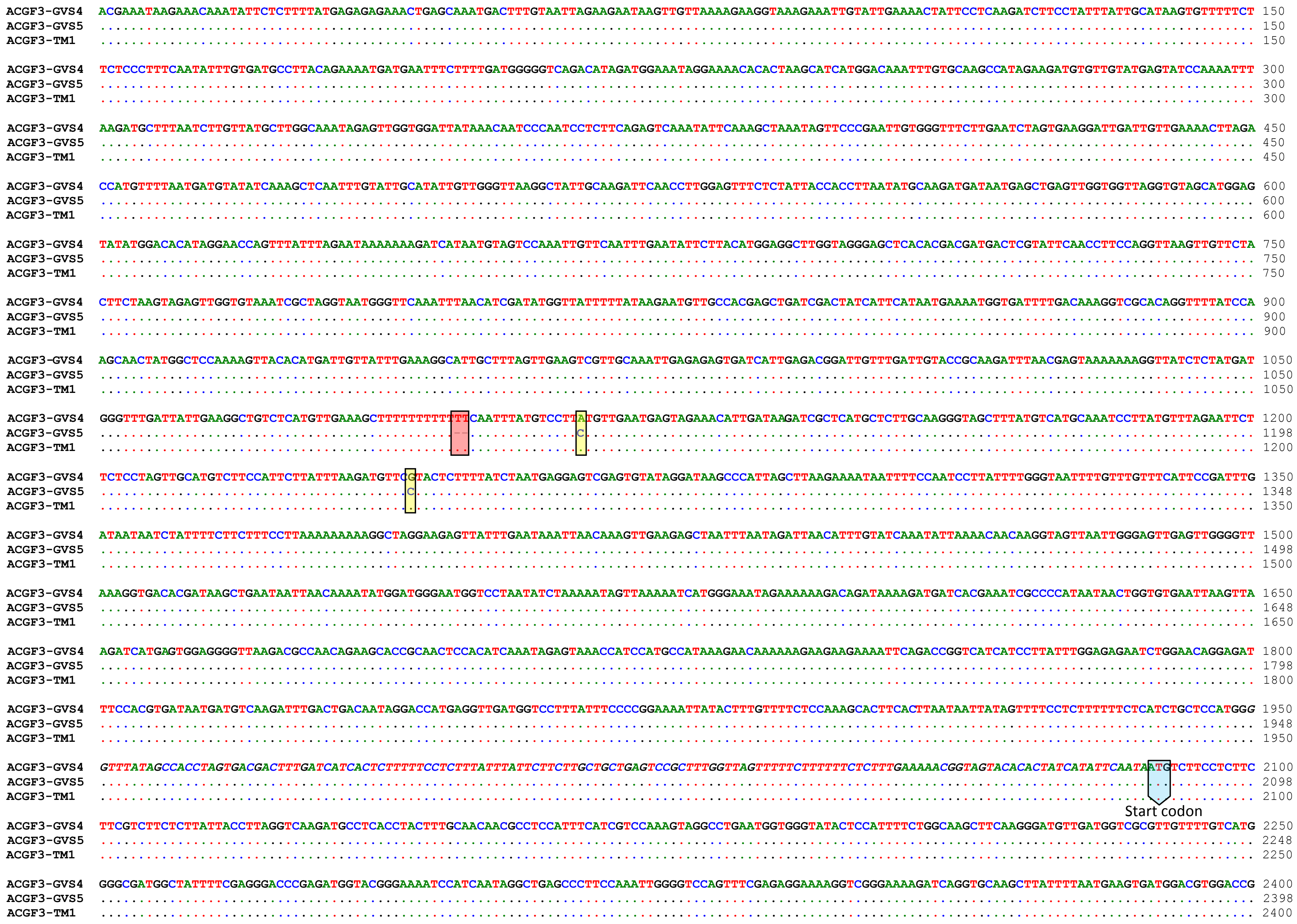
ACGF3-GVS4
ACGF3-GVS5
ACGF3-TM1
Start codon

```
ACGF3-GVS4  TATGGTAGATGGCGATGTGACTGATTATGAGTGGTACTATACCGTATC------------------------------------------------------------------------------------------------------ 2448
ACGF3-GVS5  ................................................TGTTGCGGAAAGGATCGATTCGAGAACTCCGATATGACTACAAGTAAATTGACCGGAACGAAAAATAAAGTGAACACAAGGATTTACGTGGTTCGGCTTTAA 2548
ACGF3-TM1   ................................................------------------------------------------------------------------------------------------------------ 2448

ACGF3-GVS4  ------------------------------------------------------------------------------------------------------------------------------------------------------ 2448
ACGF3-GVS5  TGCCTACGTCCACGGGCAGAGGCGAAAAGAAATTTCACTATAACAAGATGAAGATTACAAGTGTTTTACACTCAAGACACAACCCGAGAACCTCACAACTCTCAACCCCTATAGAAAACCCGAAAGCTAAAATCCTAGCTTTCAAAGAAC 2698
ACGF3-TM1   ------------------------------------------------------------------------------------------------------------------------------------------------------ 2448

ACGF3-GVS4  ------------------------------------------------------------------------------------------------------------------------------------------------------ 2448
ACGF3-GVS5  AAGCTTTGCTCTCTCTTGGTTTGGGATGATGAATATGGCTAATGAACCTCTCTATTTATAAGAGAGTTCAAGGACATAATTGTCCAAAACTTTGGCAAAGATTTGTGGTTGAAAATGGACACCAACAACCATCCACTAATCCACTACCAC 2848
ACGF3-TM1   ------------------------------------------------------------------------------------------------------------------------------------------------------ 2448

ACGF3-GVS4  ------------------------------------------------------------------------------------------------------------------------------------------------------ 2448
ACGF3-GVS5  CAACAACCCACTACCAACAACAACAATCCACTACCAATTTTAAGCCATTTCTTAACAAATCTCCACCTTGGCTTGAAATTTACCAAGCTGAACATCATAGTGAATTCCTCGAACTGGATCACCTCTTCCAAACGCCTCTCTGGCGCTAAT 2998
ACGF3-TM1   ------------------------------------------------------------------------------------------------------------------------------------------------------ 2448

ACGF3-GVS4  ------------------------------------------------------------------------------------------------------------------------------------------------------ 2448
ACGF3-GVS5  CAATCCCGAATACCTATCAAGTCCAAGCAATGCTTGAACTTATATGCAGGGATCACCTTAGTTAACATATCTGCAGGATTCTTCTCGGTAGCAACCTTGCTGATAACAATGTCACCTTGCGTAACATGTTCCCGAACAAAATGATATCTG 3148
ACGF3-TM1   ------------------------------------------------------------------------------------------------------------------------------------------------------ 2448

ACGF3-GVS4  ------------------------------------------------------------------------------------------------------------------------------------------------------ 2448
ACGF3-GVS5  ACATCAATGTGTTTGGTCCGTTCATGAAACATCTGATTTTTAGTCAGATGTATGGCACTCTGGCTATCGCAAAATACAACAGTGAAATCCTGTTGTAAACCCAAACTGCTTACTAGACCTTTCATCCACAATGCTTCTTTCACTGCTTCT 3298
ACGF3-TM1   ------------------------------------------------------------------------------------------------------------------------------------------------------ 2448

ACGF3-GVS4  ------------------------------------------------------------------------------------------------------------------------------------------------------ 2448
ACGF3-GVS5  GCTAACGCCATATATTCCGCCTCAGTCGTAGATAAGGCTACGGTAGACTGTAACACAGCTTTCCAACTAATGGCTCCTCCAGAATAAGTGAAAACATAACCCGTCAGAGATCTTCTTTTGTCCAGATCTCCAGCATAATCAGAGTCCACA 3448
ACGF3-TM1   ------------------------------------------------------------------------------------------------------------------------------------------------------ 2448

ACGF3-GVS4  ------------------------------------------------------------------------------------------------------------------------------------------------------ 2448
ACGF3-GVS5  TAGCCCGTGACACTGCTAGTGCAGTCACTCTGATCATATACCAAGCATAAATCTGCAGAACCTCTCAAGTACCTGAGAATCCATTTCACGGCCTGCCAGTGTTCCTTGCCAGGACAACTCATGTATCTGCTGACCACACTAACTGCATGT 3598
ACGF3-TM1   ------------------------------------------------------------------------------------------------------------------------------------------------------ 2448

ACGF3-GVS4  ------------------------------------------------------------------------------------------------------------------------------------------------------ 2448
ACGF3-GVS5  GAAATGTCTGGACGAGTGCAAACCATTGCATACATCACGCTTCCAACTGCACTCGAGTATGGAATGTGAGACATTTGCTGCTTTTCTTCATCAGATTGTGGTGACAACTCTGCAGAGAGTTTGAAATGTGGTGCCAACGGAGTACTCACA 3748
ACGF3-TM1   ------------------------------------------------------------------------------------------------------------------------------------------------------ 2448

ACGF3-GVS4  ------------------------------------------------------------------------------------------------------------------------------------------------------ 2448
ACGF3-GVS5  GTTTTCGCTTTATCCATGCCGAAGCGTTGAAGAACCTTTTCAATGTAGTTCTTCTGCGACACACGAAGCTTGCCCGCCTTGCGATCTCTGTGAATATCCATGCCCAAAATTTTCTTGGCAGCACCCAGATCCTTCATCTCGAACTCACCA 3898
ACGF3-TM1   ------------------------------------------------------------------------------------------------------------------------------------------------------ 2448

ACGF3-GVS4  ------------------------------------------------------------------------------------------------------------------------------------------------------ 2448
ACGF3-GVS5  CTCAACTGCGACTTTAACTTGTTGATTTCCGACATGTTTTTGGAAGCAATTAACATGTCATCAACATACAGCAACAAATAAATGTGCGAACCATCTGAGAGCTTCCGATGATAGACACAAGCATCATAGTCACATCTTGTGTAACCATGC 4048
ACGF3-TM1   ------------------------------------------------------------------------------------------------------------------------------------------------------ 2448

ACGF3-GVS4  ------------------------------------------------------------------------------------------------------------------------------------------------------ 2448
ACGF3-GVS5  TGAATCATGAAGCTATCAAACCGCTTGTACCACTGCCTTGGGGATTGTTTCAATCCATATAAAGACTTCTTTAATAGACAGACATGGTCTTCTTTACCAGGAACTGTAAAACCCTCTGGTTGACGCATGTAGATTGTTTCCTCGAGCTCA 4198
ACGF3-TM1   ------------------------------------------------------------------------------------------------------------------------------------------------------ 2448

ACGF3-GVS4  ------------------------------------------------------------------------------------------------------------------------------------------------------ 2448
ACGF3-GVS5  CCATGCAAGAACGCCGTTTTTACGTCAAGCTGCTCAAGTTCTAGATCAGACTTGGCCACCATGGCAAGTAATACACGAATGGAAGAATGCTTTACGACTGGGGAGAAAACTTCATTGTAGTCAATCCCCTCTTTCTGAGTGAAGCCTTTA 4348
ACGF3-TM1   ------------------------------------------------------------------------------------------------------------------------------------------------------ 2448

ACGF3-GVS4  ------------------------------------------------------------------------------------------------------------------------------------------------------ 2448
ACGF3-GVS5  GCAACCAATCGTGCCTTGAATCTAGTTGCTTCAACCCCTAGGATGCCTTCCTTTTTCTTGAAGACCCATTTGCAACCAACTATCTTCTGGTTACTTGGCGGCTTAACCAACTCCCAAGTATGGTTCTTGTGAAGAGATTCTATCTCCTCA 4498
ACGF3-TM1   ------------------------------------------------------------------------------------------------------------------------------------------------------ 2448

ACGF3-GVS4  ------------------------------------------------------------------------------------------------------------------------------------------------------ 2448
ACGF3-GVS5  CTCATAGCAATTGCCCACTGTGCCGATTCATCACACGTGACAGCCTCATTATAACTGGAAGGTTCTATACCAATGGACTCCGCCACACTGAGCGCGAAAGACACCAGATTAGCGTAACGCGGATTTGGTTTGATTTGTCTCTTCGTTCTT 4648
ACGF3-TM1   ------------------------------------------------------------------------------------------------------------------------------------------------------ 2448

ACGF3-GVS4  ------------------------------------------------------------------------------------------------------------------------------------------------------ 2448
ACGF3-GVS5  CCAGTGGCAATGCTATATGGTTTTTCTTGAGGTGACTCATCTTCATCGGAATCTTGCACCTCAACTTGATCATCCTGGACTGAAGTACCTTCCGTAGGAATTGGAGCGTCCACCTGACACTCCACCTGCTTCTCAACACCGTGATCTCCC 4798
ACGF3-TM1   ------------------------------------------------------------------------------------------------------------------------------------------------------ 2448
```

```
ACGF3-GVS4  ------------------------------------------------------------------------------------------------------------------------------------------------------ 2448
ACGF3-GVS5  ATTCTATCTGACTCCTCCTTTTCCCGGGAATTTGTGGTGGATCGAAGCATGGATGACTCATCAAAAGTCACATCTCTGCTGATGATGAACTTGGACGAAACCGGATCAGGACACCACAACCTGTATCCTTTCACCCCTTGGCCGTATCCA 4948
ACGF3-TM1   ------------------------------------------------------------------------------------------------------------------------------------------------------ 2448

ACGF3-GVS4  ------------------------------------------------------------------------------------------------------------------------------------------------------ 2448
ACGF3-GVS5  AGAAATATGCATTTCTTCGCCCTCGGTTTGAGTTTTCCCTCATTTACATGAGCATACGCAGGGCAGCCAAACACTCTTAAACCAGAGTAATCAGCAGGAGAACCAGACCATACTTCCTCAGGAGTCTTCAGCTCAATAGCTGTTGATGGA 5098
ACGF3-TM1   ------------------------------------------------------------------------------------------------------------------------------------------------------ 2448

ACGF3-GVS4  ------------------------------------------------------------------------------------------------------------------------------------------------------ 2448
ACGF3-GVS5  GATCTGTTAACCAAATAACAAGCAGTATTAACAGCTTCAGCCCAAAATTCTTCACCGAGCCCAGCATTTGATCGCATGCAACGAGCTCGCTCCAAGAGAGTTCTATTCATGCGTTCTGCAACTCCATTTTGTTGTGGTGTTCGACGAACA 5248
ACGF3-TM1   ------------------------------------------------------------------------------------------------------------------------------------------------------ 2448

ACGF3-GVS4  ------------------------------------------------------------------------------------------------------------------------------------------------------ 2448
ACGF3-GVS5  GTGCGGTGTCTCACTATTCCTTCATTTTTGCAGAACTCATTGAATTCACCTGAGCAAAACTCCAAGCCATTATCCGTCCGGAATCGCTTGATCTTCTTTCCTGTTTGATTTTCGATCAAAGCTTTAAATTGCTTGAAGTTGATGAGAACC 5398
ACGF3-TM1   ------------------------------------------------------------------------------------------------------------------------------------------------------ 2448

ACGF3-GVS4  ------------------------------------------------------------------------------------------------------------------------------------------------------ 2448
ACGF3-GVS5  TCATACTTGCTCTTCAGAAAATACACCCAAACCTTTCTCGAGTAATCATCGATAAAGGTAAGCAGATATCTGTAACCACCTTTAGAAATTGTCGGAGAAGGCCCCCAAAGGTCAGAATGGAAGTAATCCACTGTGCCTTTTGTCTTGTGA 5548
ACGF3-TM1   ------------------------------------------------------------------------------------------------------------------------------------------------------ 2448

ACGF3-GVS4  ------------------------------------------------------------------------------------------------------------------------------------------------------ 2448
ACGF3-GVS5  ATCCCAGTGCTGAACTTTACCCGAGTCTGCTTACCGAAGACGCAGTGCTCACAGAAATTCAACTTTCCTGTACACTGCCCAGACAATAATCCTCGTTTGCTTAGCACCGATAAGCCTCTCTCGCTCATATGCCCGAGCCGCATATGCCAT 5698
ACGF3-TM1   ------------------------------------------------------------------------------------------------------------------------------------------------------ 2448

ACGF3-GVS4  ------------------------------------------------------------------------------------------------------------------------------------------------------ 2448
ACGF3-GVS5  AACTTCGTGGTGTCAGAATCTAGATCATCTGATGATGACACTCCCGCAACACCTGTAACCGAGGAACCATCGAGGAAGTAAAGGCCCCGCTCCAAATTACCACGTATCACAGTCAAAGCACCCGAGAATACCTTGAGAACTCCACCTTCA 5848
ACGF3-TM1   ------------------------------------------------------------------------------------------------------------------------------------------------------ 2448

ACGF3-GVS4  ------------------------------------------------------------------------------------------------------------------------------------------------------ 2448
ACGF3-GVS5  GCAGAGTACCGAAAGCCTTTCTTGTCCAACGTACTCAAAGAGATCAAATTTTTCTTCATTTCTGGAATGTGCCGAACATCAGTTAGAGTTCTAACAATACCGTCAAACATCTTTATACGAACTGTGCCAATCCCCATGACTTGACATGCG 5998
ACGF3-TM1   ------------------------------------------------------------------------------------------------------------------------------------------------------ 2448

ACGF3-GVS4  ------------------------------------------------------------------------------------------------------------------------------------------------------ 2448
ACGF3-GVS5  TGGTCATTTCCCATTAGTACTGAACCAGAATGCTTCTCGTATGTTGAGAATGCATCTTTCGAAGTACTTATATGAAATGTAGCTCCCGTATCAAGAATCCATCTCCCACCAGCGTAAGAGTCGGATACTGCAAGAACGATCTCGGCATCA 6148
ACGF3-TM1   ------------------------------------------------------------------------------------------------------------------------------------------------------ 2448

ACGF3-GVS4  ------------------------------------------------------------------------------------------------------------------------------------------------------ 2448
ACGF3-GVS5  CTTGAAGAATCTGCATCAGCTACATTCGCACGATCATTTTGTTGCTCCTGTGATTCTGATTTCTCCTTCCTTTTCGGACAATCTACCTTCATGTGCCCGTACTTCTTACAGTAGTAGCACTGTATTCTCTTCTTGGACTGCGATCTAGGA 6298
ACGF3-TM1   ------------------------------------------------------------------------------------------------------------------------------------------------------ 2448

ACGF3-GVS4  ------------------------------------------------------------------------------------------------------------------------------------------------------ 2448
ACGF3-GVS5  TGGCTTTTACTTGAACTTCCACCCTTTGCCTTGGATCTTCCTCGAGCAACCAAGCCTTCGCCTTCATTGTTTTCGACCACCTTACCAGTGATTTTCTTCCTCAACTCACTGGAACTTAAGGCATTTTTCACCTCCTCTAGAGTCAGGTCA 6448
ACGF3-TM1   ------------------------------------------------------------------------------------------------------------------------------------------------------ 2448

ACGF3-GVS4  ------------------------------------------------------------------------------------------------------------------------------------------------------ 2448
ACGF3-GVS5  TCACGACCGTACATCATTGTATCAACAAAATTCTCATACGAGGGAGGCAAAGAACACAAAACAATTATTGCTTGGTCCTCATCATCGATTTTGTTATCGATATTATTCAAATCCATGATAATGGAATTGAATTTATCCAGGTGCTGGGAA 6598
ACGF3-TM1   ------------------------------------------------------------------------------------------------------------------------------------------------------ 2448

ACGF3-GVS4  ------------------------------------------------------------------------------------------------------------------------------------------------------ 2448
ACGF3-GVS5  ACAGGTGTACCTTCCTCCATCTTCAGGGCATAGAGTCTTTGCTTGAGGTAGAGCCGGTTCGTCAATGACTTCGTCATGTACTTGCTCTCTAACCGGAGCCACAAACCGGACGCGGTTTTCTCATCCGCTACTTCTCGTAGCACTTCATCT 6748
ACGF3-TM1   ------------------------------------------------------------------------------------------------------------------------------------------------------ 2448

ACGF3-GVS4  ------------------------------------------------------------------------------------------------------------------------------------------------------ 2448
ACGF3-GVS5  CCTAGACATAGCAGAATAGCACTATGTGCTCTTTCTAGCATGTCATCCTTTTGCTCTTCCGAAAGCGTGCTTGGTAATTTATCTTTACCAGACAATGCTTTTAGCAATCCTTGTTGAACCAGCACTGCCCGCATCTTGATGCGCCATAAA 6898
ACGF3-TM1   ------------------------------------------------------------------------------------------------------------------------------------------------------ 2448

ACGF3-GVS4  ------------------------------------------------------------------------------------------------------------------------------------------------------ 2448
ACGF3-GVS5  CTGAAACTATTTTTCCCGGTAAATTTCTCGACATCATACTTAGTCGATGAAACACTTGTAGCCATAATTTGGAACCTTTGAATGAATGATAATATTTTCTTCCTGATGTGAAAGATCAGACAAAGCTGCAGTCACAGGGCAATTCAGAAA 7048
ACGF3-TM1   ------------------------------------------------------------------------------------------------------------------------------------------------------ 2448

ACGF3-GVS4  ------------------------------------------------------------------------------------------------------------------------------------------------------ 2448
ACGF3-GVS5  ATATTATCACTCCTTCAACTACGCTCTGATACCAATTTGTTGCGGAAAGGATCGATTCGAGAACTCCGATATGACTACAAGTAAATTGACCGGAACGAAAAATAAAGTGAACACAAGGATTTACGTGGTTCGGCTTTAATGCCTACGTCC 7198
ACGF3-TM1   ------------------------------------------------------------------------------------------------------------------------------------------------------ 2448
```

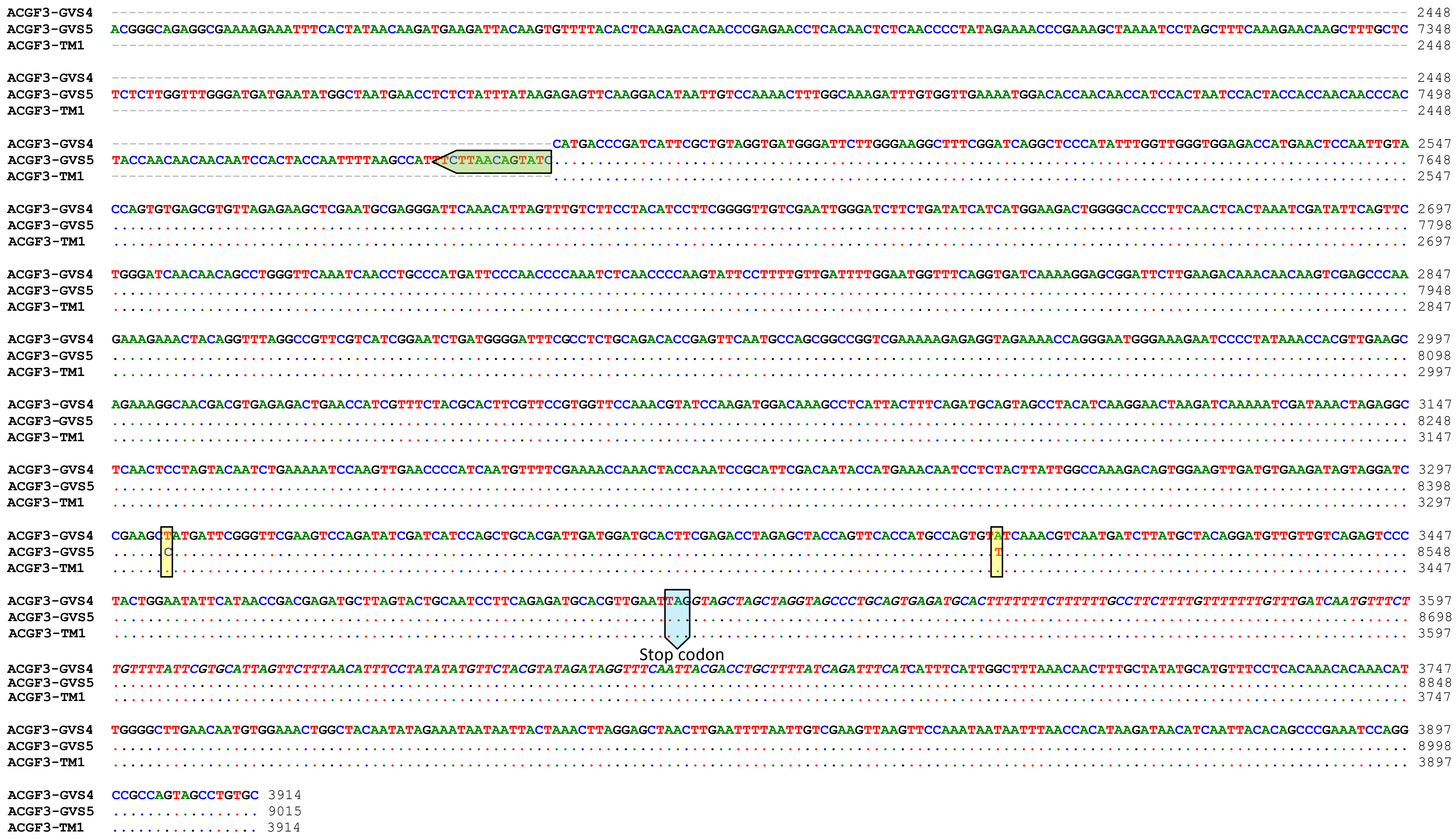

Figure S9 Alignment of A subgenome *GoPGF* (synonym *CGF3*) gene sequences from GVS4 (glanded) and GVS5 (glandless, recessive mutant) near-isogenic lines. Sequence of Texas Marker-1 (TM-1, a widely used genetic standard) is also provided for comparison purpose. The ~1.95 kb sequence upstream of the start codon, representing the promoter region of*A CGF3* in the glandless GVS5, has 2 SNPs (yellow box) and one deletion (2 bp long; red box) compared to the glanded GVS4. In addition, this homeolog in the GVS5 has two SNPs and a ~5.1 kb transposon insertion (indicated with two opposing green arrows) in the coding sequence. 5'-UTR and 3'-UTR are shown in italics. Translation initiation codon and stop codon are indicated.

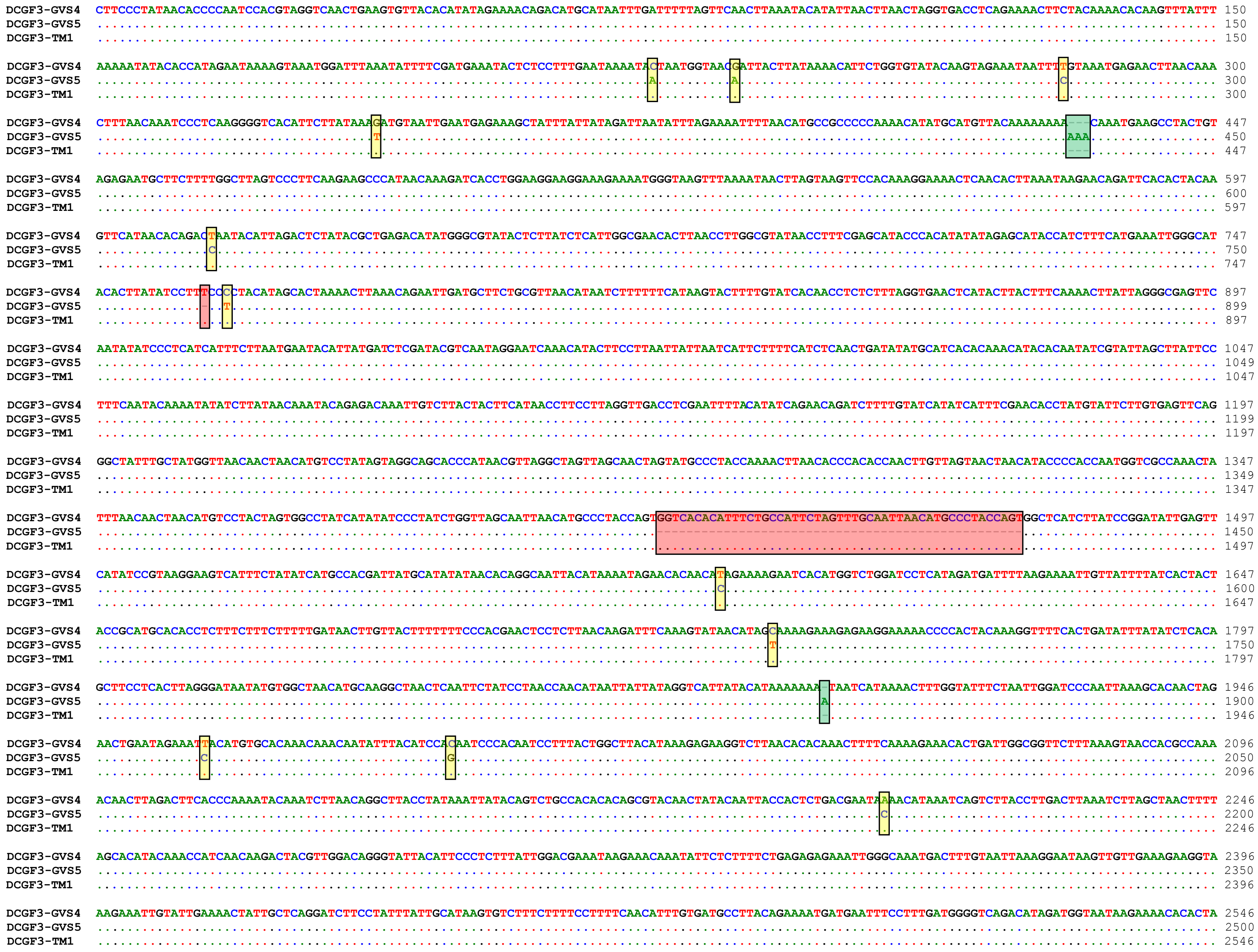
DCGF3-GVS4
DCGF3-GVS5
DCGF3-TM1

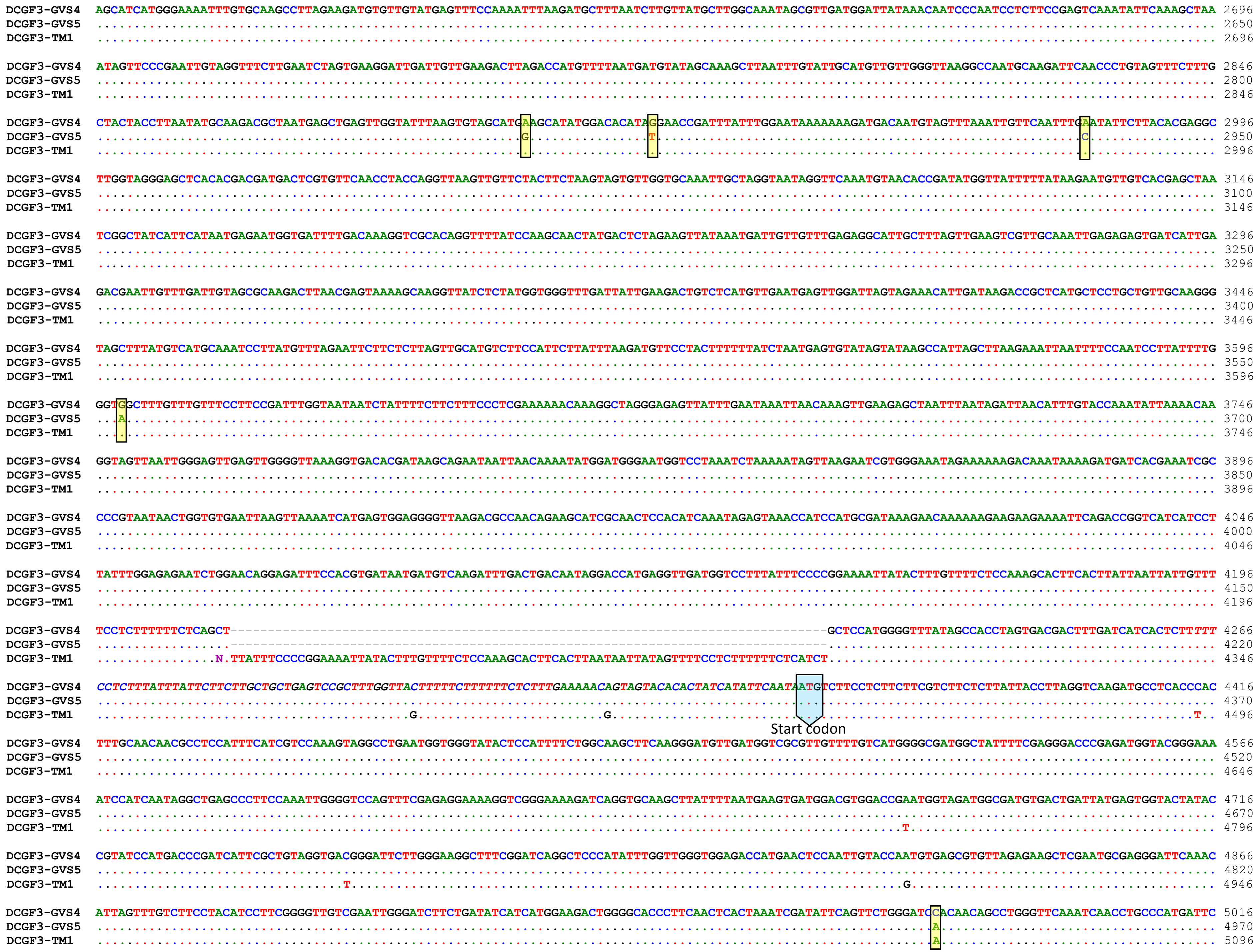
DCGF3-GVS4
DCGF3-GVS5
DCGF3-TM1
Start codon

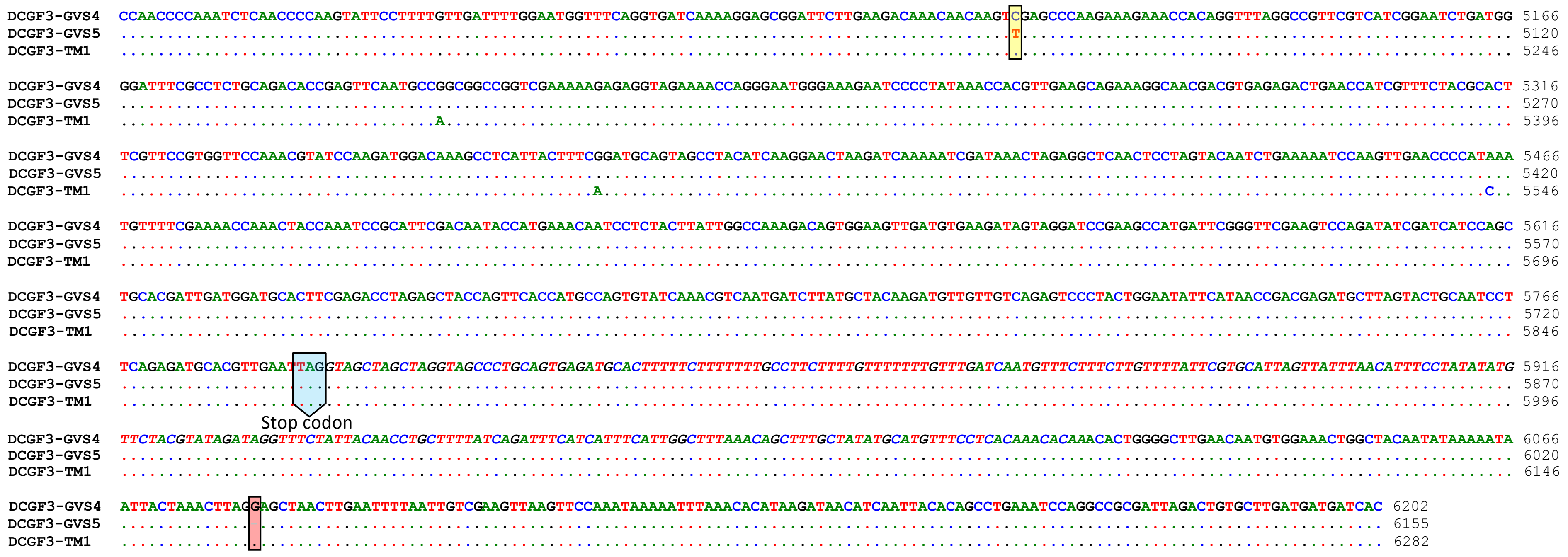

Figure S10 Alignment of D subgenome *GoPGF* (synonym *CGF3*) gene sequences from GVS4 (glanded) and GVS5 (glandless, recessive mutant) near-isogenic lines. Sequence of Texas Marker-1 (TM-1, a widely used genetic standard) is also provided for comparison purpose. The ~4.2 kb sequence upstream of the start codon, representing the promoter region of*D CGF3* in the glandless GVS5, has 15 SNPs (yellow box), two deletions (1 and 49 bp long; red box), and two insertions (1 and 3 bp; green box) compared to the glanded GVS4. In addition, the GVS5 has two SNPs in the coding sequence and one base pair deletion in the terminator. 5'-UTR and 3'-UTR are shown in italics. Translation initiation codon and stop codon are indicated.

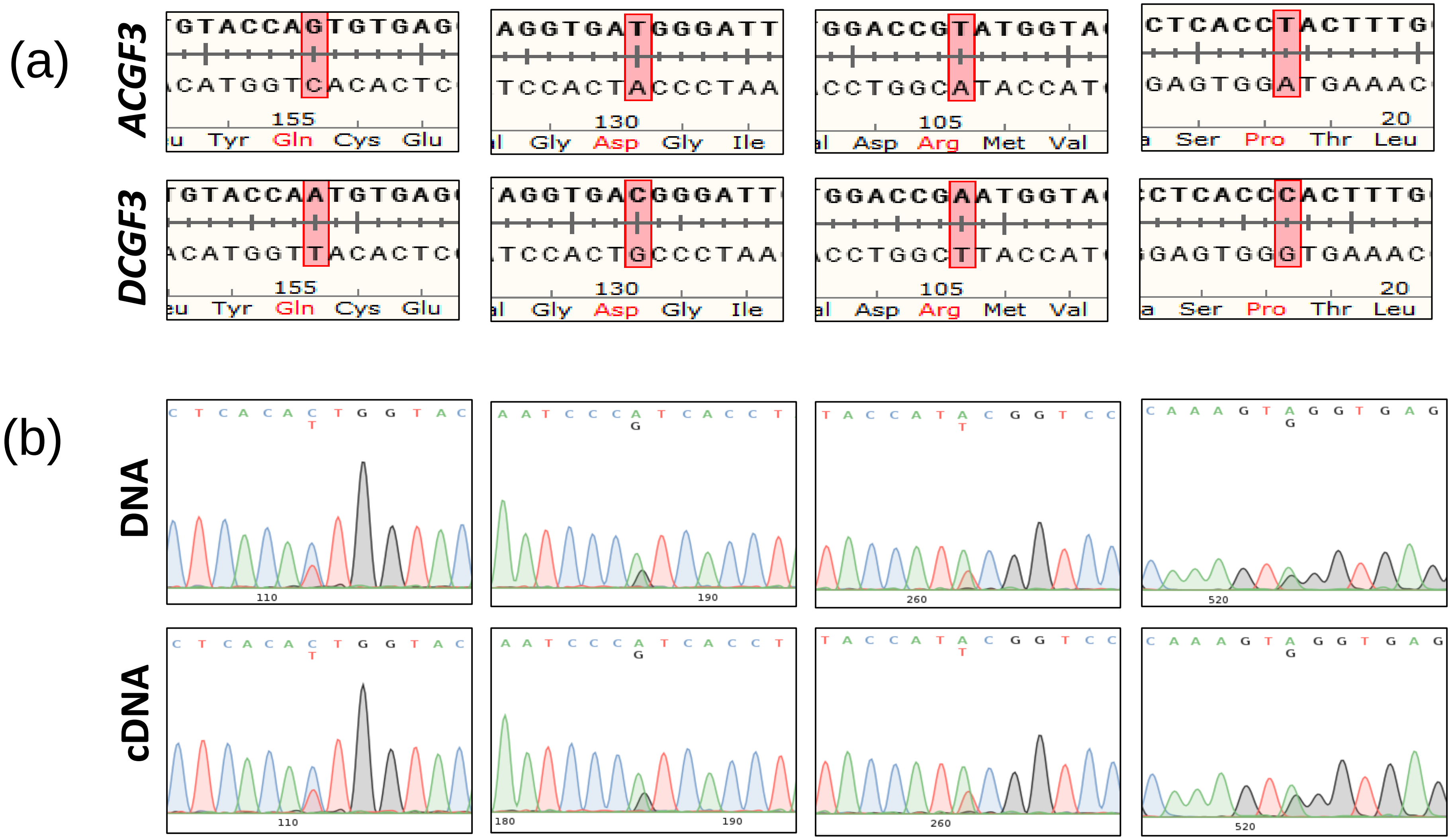

Figure S11 Sequencing results showing four different SNPs that differentiate A and D subgenome *GoPGF* (synonym *CGF3*) genes. (**a**) Segments of *GoPGF* (synonym *CGF3*)
gene sequence showing four SNPs between A and D homeologs; (**b**) chromatograms showing the expected SNPs between the two homeologs of the *GoPGF* (synonym *CGF3*) gene in genomic DNA and cDNA samples.

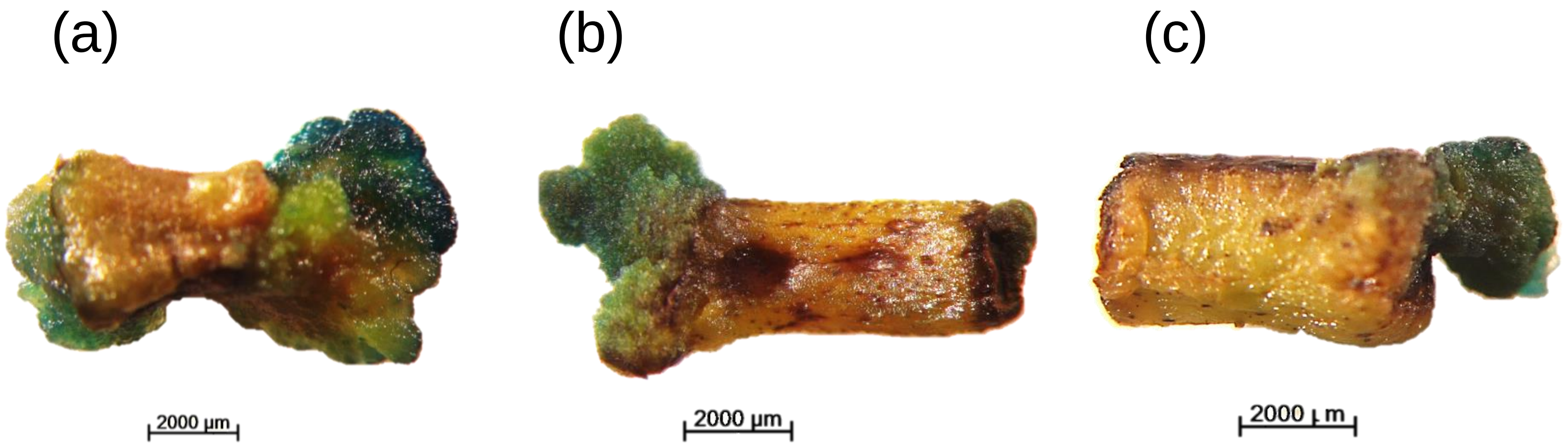

Figure S12 Promoter (2.05 kb) activity evaluation of the D subgenome *GoPGF* (synonym *CGF3*) gene from glanded and glandless cotton using *gusA* as the reporter gene. (**a**) CaMV 35S promoter (used as a positive control); (**b**) 2 kb promoter from glanded cotton; (**c**) 2 kb promoter from glandless cotton.

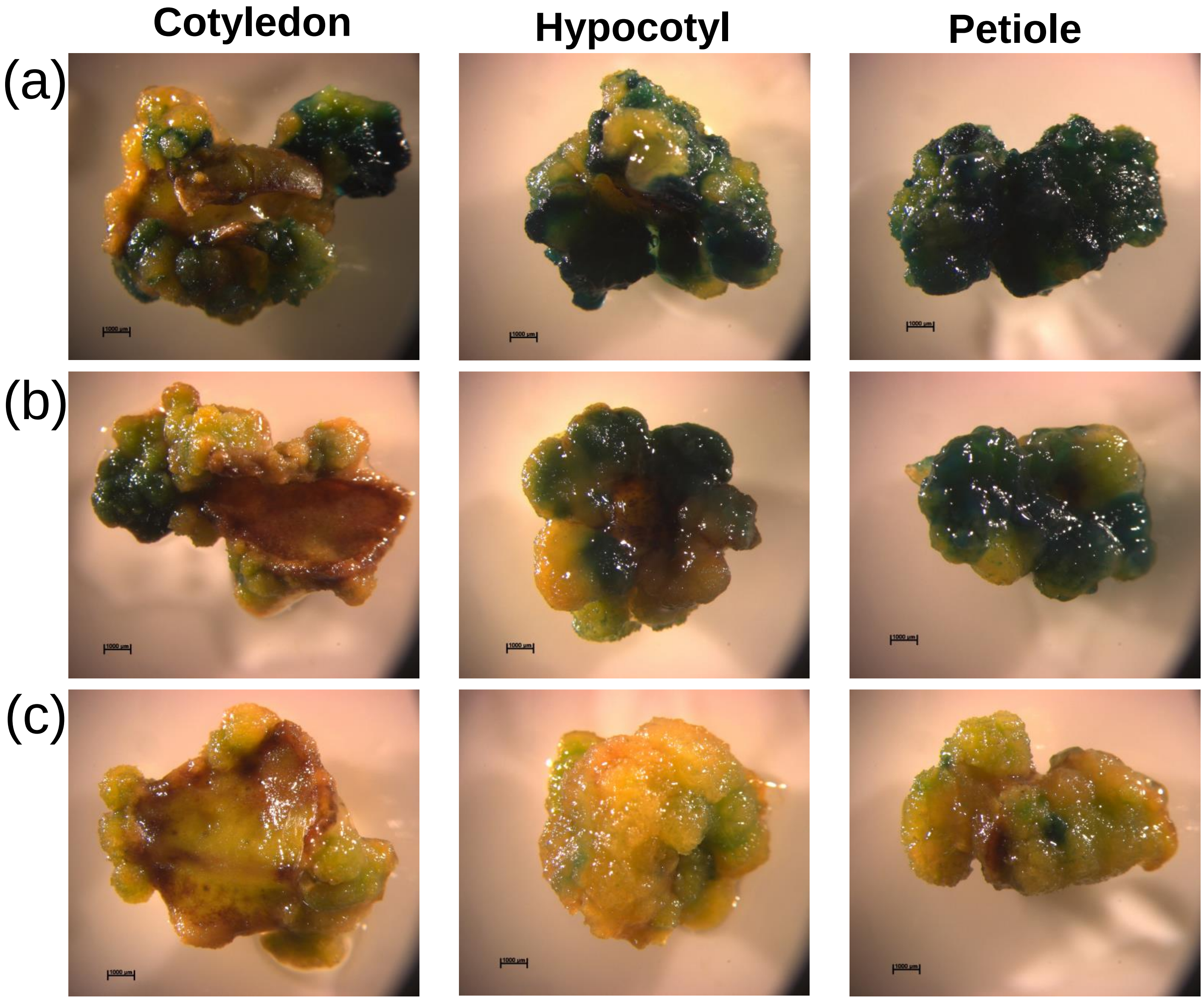

Figure S13 Promoter (~4.2 kb) activity evaluation of the D subgenome *GoPGF* (synonym *CGF3*) gene from glanded and glandless cotton using *gusA* as the reporter gene. (**a**) CaMV 35S promoter (used as a positive control); (**b**) ~4.2 kb promoter from glanded cotton; (**c**) ~4.2 kb promoter from glandless cotton.

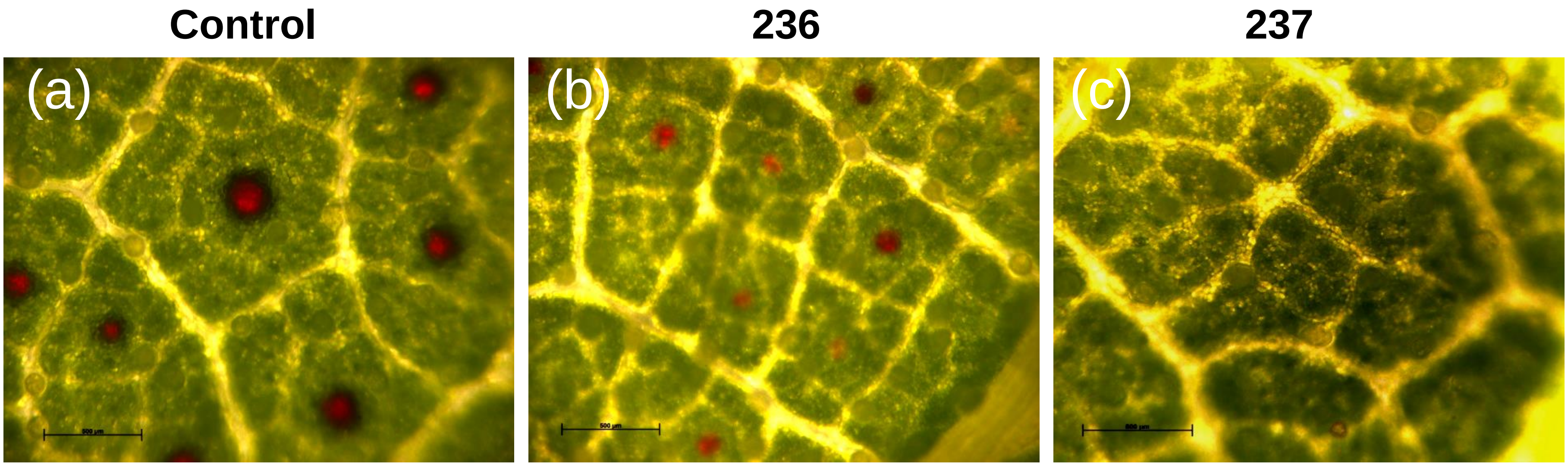

Figure S14 Effect of CRISPR/Cas9-mediated knockout of *CGF2* and *GoPGF* (synonym *CGF3*) genes on gland formation in T0 plants in comparison to glanding pattern seen in a wild-type, control cotton plant. Microscopic images of leaves undergoing expansion: (**a**) control plant showing well developed glands, (**b**) line 236-10 showing smaller and malformed glands, (**c**) line 237-3 showing complete absence of glands.

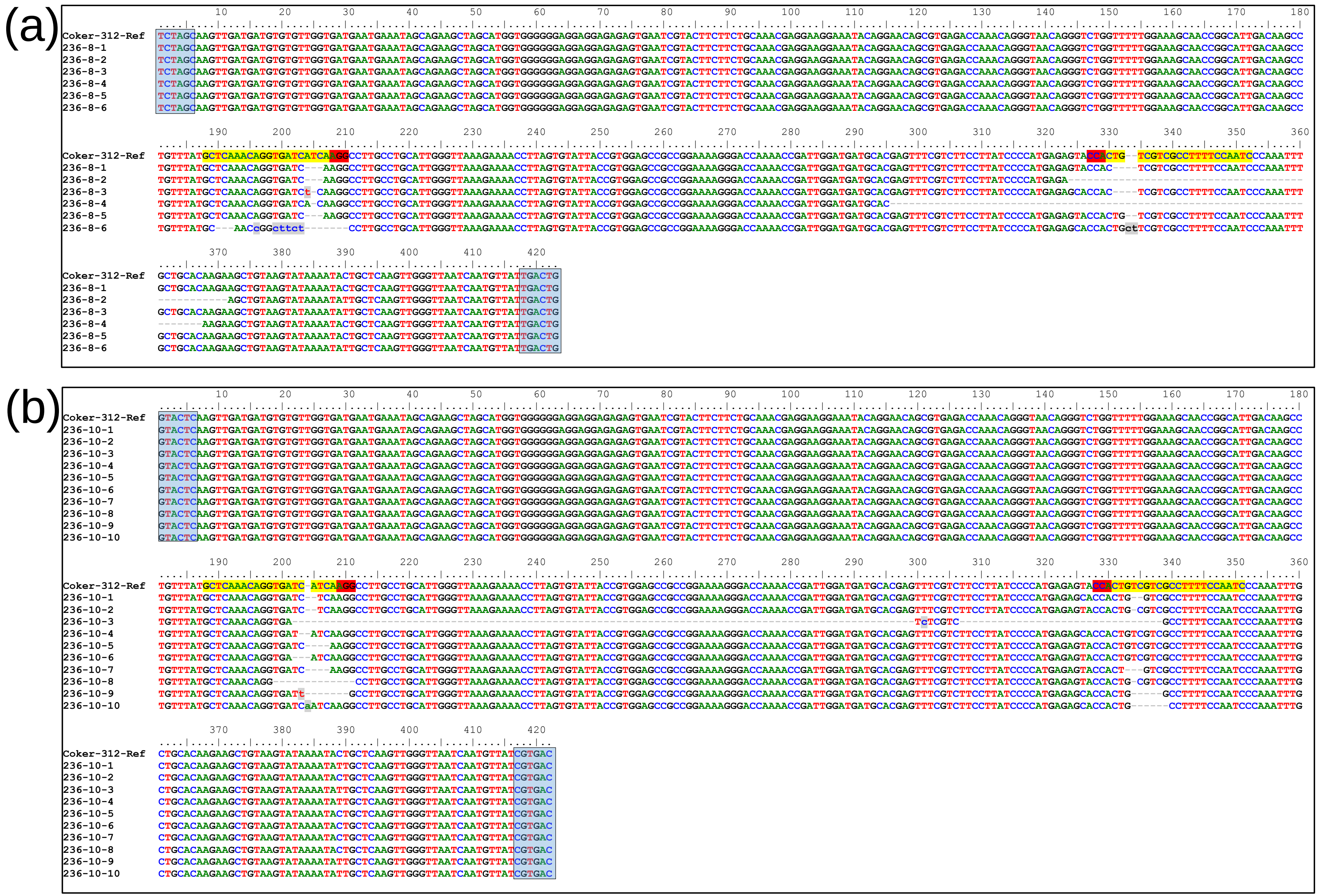

Figure S15 Mutations observed in two *CGF2* knockout lines, (**a**) 236-8 and (**b**) 236-10. The two target sites are highlighted yellow and PAM sequences red. Nucleotide insertions are shown in lower case and highlighted grey.

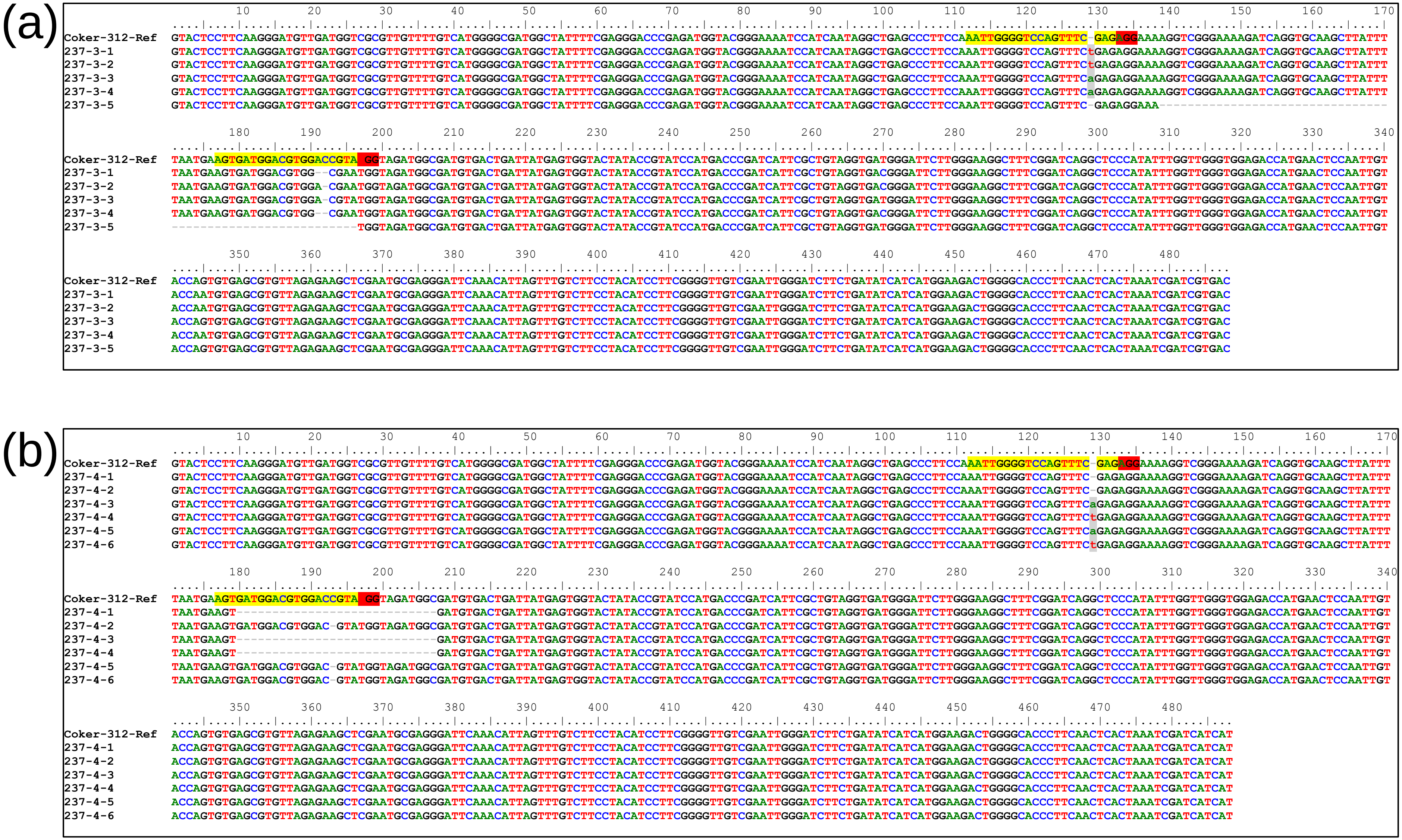

Figure S16 Mutations observed in two *GoPGF* (synonym *CGF3*) knockout lines, (**a**) 237-3 and (**b**) 237-4. The two target sites are highlighted yellow and PAM sequences red. Nucleotide insertions are shown in lower case and highlighted grey.

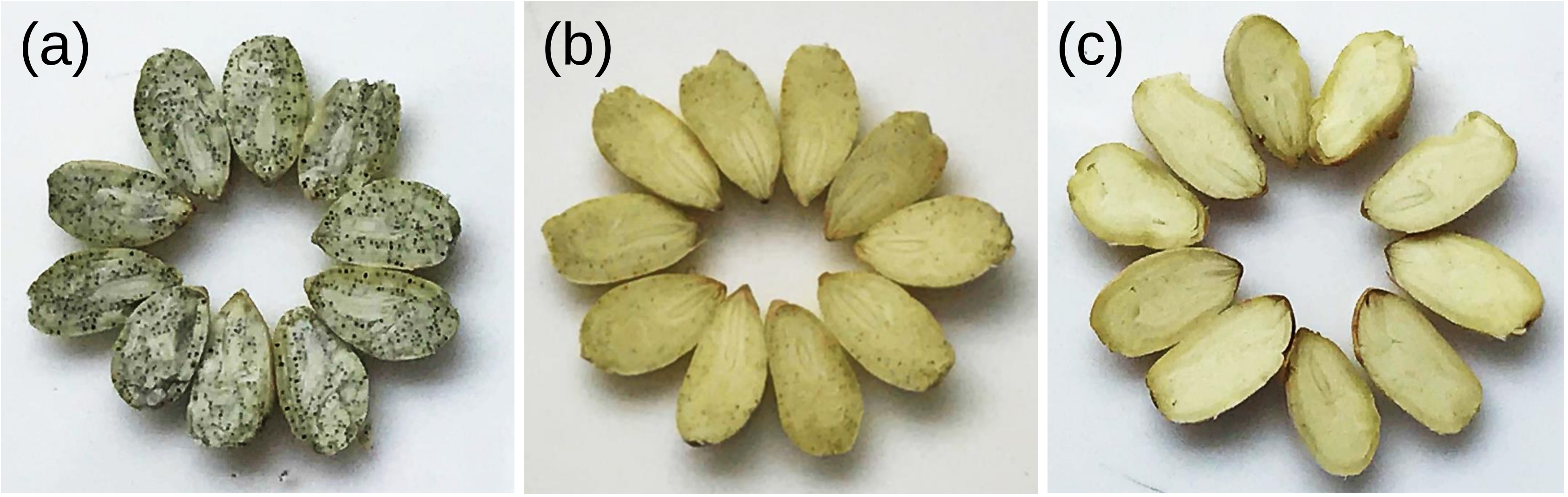

Figure S17 Effect of CRISPR/Cas9-mediated knockout of *CGF2* and *GoPGF* (synonym *CGF3*) genes on gland formation observed in cottonseed kernels. (**a**) Seeds of control plant showing well developed glands;(**b**) T1 seeds of line 236-10 showing smaller and malformed glands; (**c**) T1 seeds of line 237-3 showing complete absence of glands.

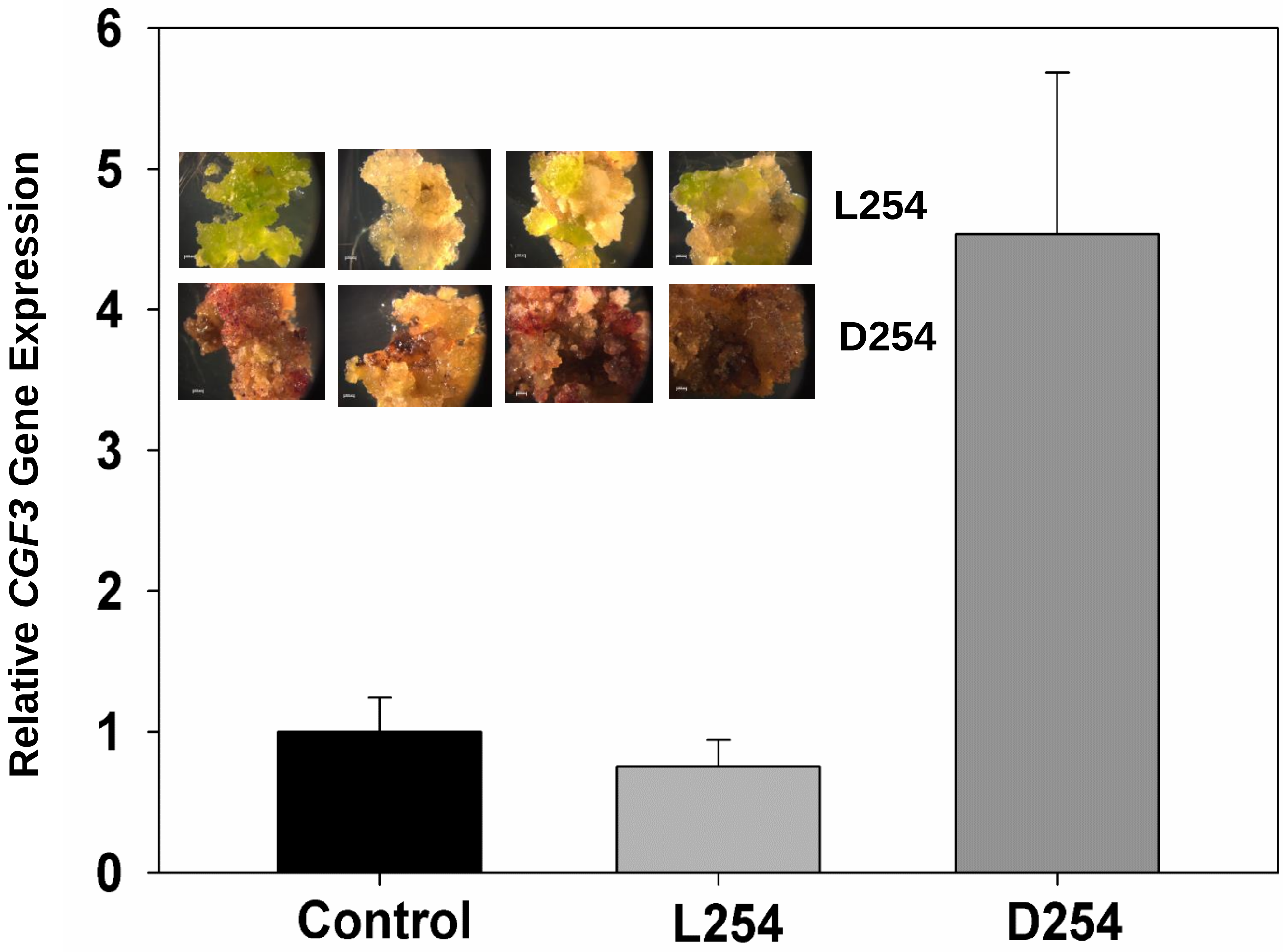

Figure S18 qRT-PCR analysis of *GoPGF* (synonym *CGF3*) transcripts in cotton callus cultures obtained following transformation with *ACGF3* overexpression construct. L254: light-colored callus lines; D254 dark-colored callus lines; Control: non transgenic callus.

Table S1 RNA-seq reads for glanded (GL; GVS4) and glandless (gl; GVS5) embryos at 14-, 16- and 32-days post-anthesis and their mapping to the reference genome.

Tissue	Quality filtered paired reads	Uniquely mapped reads	Reads mapped >1 time	Total mapped reads	Reads not mapped	% of reads not mapped	% of reads mapped one time	% of reads mapped >1 time	% total mapped
14GL	57,526,347	41,539,721	13,219,522	54,759,243	2,767,104	4.81	72.21	22.98	95.19
14gl	55,574,944	39,411,172	13,043,137	52,454,309	3,120,635	5.62	70.92	23.47	94.38
16GL	45,027,488	31,957,244	10,240,975	42,198,219	2,829,269	6.28	70.97	22.74	93.72
16gl	58,872,534	41,914,607	13,567,579	55,482,186	3,390,348	5.76	71.2	23.05	94.24
32GL	78,953,741	57,667,091	18,523,395	76,190,486	2,763,255	3.5	73.04	23.46	96.5
32gl	81,721,688	60,837,058	17,351,295	78,188,353	3,533,335	4.32	74.44	21.23	95.68
	**377,676,742**	**273,326,893**	**85,945,903**	**359,272,796**	**18,403,946**	**5.05**	**72.13**	**22.82**	**94.95**

Table S2 Genes that expressed at higher levels in the glanded embryos (STV GL; GVS4; $Gl_2Gl_2Gl_3Gl_3$) in comparison to those in the glandless (STV gl; GVS5; $gl_2gl_2gl_3gl_3$) embryos at 14-days post-anthesis stage of development based on RNA-seq analysis. Genes encoding putative transcription factors were tested for their role in gland formation using virus-induced gene silencing (VIGS). Note that because of a high degree of homology between the two homeologs, the same VIGS construct will silence both the copies in A and D subgenomes.

Gene	*G. hirsutum* Chrom-osome Number	Shrunken log fold change	FDR	Putative Function	Size bp	VIGS	Laboratory Designation
Gh_A11G0909	11 (A11)	3.459546	0.000000	Basic helix-loop-helix (bHLH) DNA-binding family protein	1488	Yes	*CGF1*
Gh_D11G1055	24 (D11)	6.412898	0.000000	Basic helix-loop-helix (bHLH) DNA-binding family protein	1488		*CGF1*
Gh_A01G0267	01 (A01)	4.992123	0.000001	NAC domain containing protein 42	960	Yes	*CGF2*
Gh_D01G0278	14 (D01)	4.656837	0.000013	NAC (No Apical Meristem) domain transcriptional regulator superfamily protein	963		*CGF2*
Gh_A12G2172	12 (A12)	6.231119	0.000000	Basic helix-loop-helix (bHLH) DNA-binding family protein	1428	Yes	*CGF3*
Gh_D12G2351	25 (D12)	4.284841	0.001531	Basic helix-loop-helix (bHLH) DNA-binding family protein	1428		*CGF3*
Gh_A10G0388		4.903984	0.000002	Jasmonate-zim-domain protein 8	363	Yes	
Gh_D10G0403		3.747897	0.003033	Jasmonate-zim-domain protein 8	363		
Gh_A12G1233		5.43426	0.000000	B-box type zinc finger family protein	393	Yes	
Gh_D12G1358		2.11097	0.000000	B-box type zinc finger family protein	393		
Gh_A01G0135		2.064435	0.000139	Zinc finger C-x8-C-x5-C-x3-H type family protein	1020	No	
Gh_A04G0546		4.370138	0.001173	HEAT repeat ;WD domain, G-beta repeat protein protein	192	No	
Gh_A05G0334		2.677935	0.000088	NAD(P)-binding Rossmann-fold superfamily protein	987	No	
Gh_A05G2973		2.888822	0.000000	Lysine histidine transporter 1	1077	No	
Gh_A06G0017		2.788916	0.000000	Thioredoxin superfamily protein	555	No	
Gh_A06G0018		2.25659	0.008180	Expansin 11	768	No	

Gh_A06G0213		2.775923	0.012662	Uncharacterized protein	957	No	
Gh_A06G1947		4.176235	0.000392	NAC domain containing protein 42	930	Yes	
Gh_A08G2056		2.912436	0.000230	NAC domain containing protein 42	867	Yes	
Gh_A10G0667		5.494345	0.000000	Pectin lyase-like superfamily protein	2202	No	
Gh_A12G1784		1.995549	0.000013	Integrase-type DNA-binding superfamily protein	711	No	
Gh_A12G2056		2.388568	0.000000	Uncharacterized protein	1251	No	
Gh_A13G0385		2.137374	0.023065	S-methyl-5-thioribose kinase	660	No	
Gh_D04G0529		3.720997	0.028702	Phosphoenolpyruvate (pep)/phosphate translocator 2	1251	No	
Gh_D05G0292		2.546437	0.031860	myb-like transcription factor family protein	768	Yes	
Gh_D05G0439		2.126081	0.007311	NAD(P)-binding Rossmann-fold superfamily protein	894	No	
Gh_D06G1859		2.756698	0.000000	P-loop containing nucleoside triphosphate hydrolases superfamily protein	987	No	
Gh_D07G2328		4.295568	0.000100	WRKY family transcription factor family protein	501	Yes	
Gh_D08G2336		3.448213	0.031860	Uncharacterized protein	345	No	
Gh_D11G0631		3.446236	0.007958	Uncharacterized protein	417	No	
Gh_D11G0996		4.052173	0.008242	Cytokinin response factor 6	894	No	
Gh_D12G1160		5.449322	0.000000	Basic helix-loop-helix (bHLH) DNA-binding family protein	1464	Yes	
Gh_Sca007330G01		4.131307	0.000000	Plant invertase/pectin methylesterase inhibitor superfamily protein	630	No	

Table S3 Primers used to amplify segments of the coding sequence of the target gene for cloning into TRV2 binary vector to conduct VIGS experiments.

Primers	Sequences (5' to 3')	size
GhA01G0267 EcoRI-F	CGgaattcCTGGGATCTCCCGAAAGCTAGC	634
GhA01G0267 SacI-R	ACGCgagctcCTCATTCTATCTGTAACATGCCATTGGC	
GhA10G0388 EcoRI-F	CGgaattcATGAGACGAAACTGCAACTTGGAG	357
GhA10G0388 SacI-R	ACGCgagctcGTAAGGAGAGGTAGCTTGGATTCG	
GhA12G2172 EcoRI-F	CGgaattcATGTCTTCCTCTTCTTCGTCTTCTC	600
GhA12G2172 SacI-R	ACGCgagctcCGATTTAGTGAGTTGAAGGGTGC	
GhA12G1233 XbaI-F	GCtctagaATGTGCAAAGGTTTACAACAAGGAAG	366
GhA12G1233 XmaI-R	TCCCcccgggGGTTGTTGAAGACTCGGTTTCCGTG	
GhD07G2328 XbaI-F	GCtctagaTCAAATGTTCTTCCCTATCTCGG	491
GhD07G2328 XmaI-R	TCCCcccgggTCAGAAGGGAGTGTAAATCTGCA	
GhD11G1055 XbaI-F	GCtctagaATGGAAGTCCTCATAATGTCTCCCTC	628
GhD11G1055 XmaI-R	TCCCcccgggCCAGACCAATGAGATCGGATTC	
GhA06G1947 XbaI-F	GCtctagaATGGAAGATGTGGAGATGGAGA	505
GhA06G1947 XmaI-R	TCCCcccgggCTTCAAAGTTGTCTTTGGCATG	
GhD05G0292 XbaI-F	GCtctagaATGGGCAGGAAATGCTCACATTG	614
GhD05G0292 XmaI-R	TCCCcccgggAATCAATGCATCCGTACTGCAAC	
GhD12G1160 XbaI-F	GCtctagaATGGAAGAACTAATCATCTCTCCATC	587
GhD12G1160 XmaI-R	TCCCcccgggGATCCAAGTTCAAGAACACCACG	
GhA08G2056 XbaI-F	GCtctagaATGAGCATGGTCCATGGCACCA	630
GhA08G2056 XmaI-R	TCCCcccgggTATCTTAACGATGGCTGCATGAACC	

Table S4 Primers used to amplify and isolate *CGF* genes from A and D subgenomes of glanded (GVS4) and glandless (GVS5) cotton plants.

Primer	Sequence 5' to 3'	Purpose
A11GhCGF-prom-3F	CTCTCCAAAATCAACCATACTCACAAATGCCTAC	To amplify CGF1 from A genome
A11GhCGF-term-R	CTCCATGGCATCCTCAAGTCACAG	
D11GhCGF-prom-F	ATCTTCTCACTCCGAAACCGACC	To amplify CGF1 from D genome
D11GhCGF-term-R	TGGAAGAAACAAGATCGGATGTGGC	
A01CGF2-P-F	GGCTGTCAGATGTAGTAAAATCAGTATTGGT	To amplify CGF2 from A genome
A01CGF2-T-R	CAAATATATATGGGTCTGATATGCATGTCTCC	
D01CGF2-P-F	CAAAGTGTTGATTTCAGCAATAACTTGTAGC	To amplify CGF2 from D genome
D01CGF2-T-R	CGTAACAAAATGGTTTTCGTATGTTACGTATC	
A12CGF-prom-F	CATCCCATACAAACTATTAACAAGATTACGTCGGATG	To amplify CGF3 from A genome
A12CGF-term-R	GTGATCATCATCAAGCACAGGCTACTG	
D12CGF-Prom-F	CAAACCATCAACAAGACTACGTTGGACA	To amplify CGF3 from D genome
D12CGF-term-R	CTAATTTAAGTGATCATCATCAAGCACAGTCTAATC	

Table S5 Guide sequences used to target *CGF2* and *GoPGF* (synonym *CGF3*) genes.

Target name	Sequence 5' to 3'
CGF2-guide-1	GCTCAAACAGGTGATCATCA
CGF2-guide-2	GATTGGAAAAGGCGACGACAG
CGF3-guide-1	AATTGGGGTCCAGTTTCGAG
CGF3-guide-2	AGTGATGGACGTGGACCGTA
CGF3-guide-3	GCTTCTCTAACACGCTCACAC

Table S6 Primers used for amplicon sequencing of regenerated plants targeted with LCT236, LCT237 and LCT238 constructs. These primers contained barcodes to distinguish amplicons from different plants.

Primers	Sequences (5'-3')
A_CGF2.Ampseq-F	gtactcAAGTTGATGATGTGTGTTGGTGATG
B_CGF2.Ampseq-F	tctagcAAGTTGATGATGTGTGTTGGTGATG
C_CGF2.Ampseq-F	gagtcaAAGTTGATGATGTGTGTTGGTGATG
D_CGF2.Ampseq-F	gctagtAAGTTGATGATGTGTGTTGGTGATG
E_CGF2.Ampseq-F	atgctaAAGTTGATGATGTGTGTTGGTGATG
F_CGF2.Ampseq-F	ctgcgaAAGTTGATGATGTGTGTTGGTGATG
G_CGF2.Ampseq-R	cgactgATAACATTGATTAACCCAACTTGAGC
H_CGF2.Ampseq-R	tgatagATAACATTGATTAACCCAACTTGAGC
I_CGF2.Ampseq-R	gtcacgATAACATTGATTAACCCAACTTGAGC
J_CGF2.Ampseq-R	atgatgATAACATTGATTAACCCAACTTGAGC
K_CGF2.Ampseq-R	cagtcaATAACATTGATTAACCCAACTTGAGC
L_CGF2.Ampseq-R	acgtcaATAACATTGATTAACCCAACTTGAGC
A_CGF3.Ampseq-F	gtactcCTTCAAGGGATGTTGATGGTCG
B_CGF3.Ampseq-F	tctagcCTTCAAGGGATGTTGATGGTCG
C_CGF3.Ampseq-F	gagtcaCTTCAAGGGATGTTGATGGTCG
D_CGF3.Ampseq-F	gctagtCTTCAAGGGATGTTGATGGTCG
E_CGF3.Ampseq-F	atgctaCTTCAAGGGATGTTGATGGTCG
F_CGF3.Ampseq-F	ctgcgaCTTCAAGGGATGTTGATGGTCG
G_CGF3.Ampseq-R	cgactgATCGATTTAGTGAGTTGAAGGGTGC
H_CGF3.Ampseq-R	tgatagATCGATTTAGTGAGTTGAAGGGTGC
I_CGF3.Ampseq-R	gtcacgATCGATTTAGTGAGTTGAAGGGTGC
J_CGF3.Ampseq-R	atgatgATCGATTTAGTGAGTTGAAGGGTGC
K_CGF3.Ampseq-R	cagtcaATCGATTTAGTGAGTTGAAGGGTGC
L_CGF3.Ampseq-R	acgtcaATCGATTTAGTGAGTTGAAGGGTGC
