# Supplementary material for: Genes regulating gland development in the cotton plant
Source: Plant Biotechnol J. 2018 Dec 21;17(6):1142–53. doi: 10.1111/pbi.13044 (PMC6523598; doi:10.1111/pbi.13044)
Supplement: Supplementary file 3 — Data S2 Supplementary methods. [file PBI-17-1142-s001.docx]

**Genes regulating gland development in the cotton plant**

Madhusudhana R. Janga^1^, Devendra Pandeya^1^, LeAnne M. Campbell^1^, Kranti Konganti^2^, Stephany Toinga Villafuerte^1^, Lorraine Puckhaber^3^, Alan Pepper^4^, Robert D. Stipanovic^3^,

Jodi A. Scheffler^5^, Keerti S. Rathore^1,6*^

^1^Institute for Plant Genomics & Biotechnology, Texas A&M University, College Station, TX, 77843-2123, USA; ^2^Texas A&M Institute for Genome Sciences and Society, Texas A&M University, College Station, TX, 77843; ^3^Southern Plains Agricultural Research Center, USDA-ARS, College Station, TX, USA; ^4^Dept. of Biology, Texas A&M University, College Station, TX, 77843; ^5^Crop Genetics Research Unit, USDA-ARS, Stoneville, MS, USA; ^6^Dept. of Soil & Crop Sciences, Texas A&M University, College Station, TX, 77843-2474, USA.

^*^Corresponding author: Keerti S. Rathore

Institute for Plant Genomics & Biotechnology

Dept. of Soil & Crop Sciences

Texas A&M University

College Station, TX 77843-2123, USA

Email: [rathore@tamu.edu](mailto:rathore@tamu.edu)

Phone: (979) 862 4795

Fax: (979) 862 3414

**Data S2**

**Supplementary Methods**

**Virus induced gene silencing**

VIGS vector construction and infiltration were performed as described by Dinesh-Kumar et al. (2003); Gao et al. (2011) with slight modifications. A segment of the coding sequence of the target gene was amplified using the primers containing appropriate restriction enzyme sequence (Table S4), and cloned into TRV2 binary vector. Each vector was then mobilized into *A. tumefaciens* cells (GV3101). The cotyledons of 12-days-old cotton (*G. hirsutum,* cv. Coker 312, a glanded cultivar) seedlings were infiltrated with GV3101 strains containing TRV1 (pYL192) and GV3101 strains containing TRV2 (pYL156 carrying the target gene sequence selected to silence a particular gene/gene homeologs) in a ratio of 1:1 (v/v). After infiltration, the plants were covered with Humidome^TM^ and kept in dark for 24 h. The next day, plants were transferred to a growth chamber maintained at 12 h light/12 h dark cycle and 23˚C. The second true leaf from each plant was harvested three weeks after infiltration for gland counting and terpenoid analyses. Glands were counted on the scanned image of the leaf using ImageJ software. Results from an empty vector control and another control targeting the GhCLA gene to confirm the efficacy of VIGS are shown in Figure S17.

**Terpenoid estimation in the leaves and callus tissue**

In case of VIGS experiment, the second true leaf was harvested three weeks after *Agrobacterium* infiltration and frozen in liquid Nitrogen. The *CGF2* and *GoPGF* (synonym *CGF3*) knockout lines were grown in the greenhouse for about two months. One or two young, expanding leaves were harvested and immediately frozen in liquid nitrogen. At four months following transformation with an *ACGF3* overexpression vector, callus lines representing individual events were segregated based on their light or dark colored appearance and frozen in liquid nitrogen. The leaves and callus tissues were freeze-dried at -20 °C for 60 h and 168 h, respectively. Each leaf sample was ground to a fine powder and 100 mg was used for extraction using acetonitrile:water:phosphoric acid (80:20:0.1). A 50 µL fraction of the extract was analyzed on a LC-1200 (Agilent Technology) High Pressure Liquid Chromatograph equipped with diode array detector for compound spectral identification as described by Stipanovic et al. (1988). Results are reported as µg terpenoid per mg dry weight of tissue.

**cDNA amplification and qRT-PCR**

Total RNA (800 ng) was reverse transcribed in 20 µl volume using an oligo poly-T primer and MultiScribe^TM^ Reverse Transcriptase (Taqman RT kit; Applied Biosystems, Foster City, CA) following manufacturer’s instructions. The cDNA amplification conditions were as follows: 25 °C for 10 min, 48 °C for 30 min, and 95 °C for 5 min. The cDNA was diluted to 100 µl, PCR was performed to check the cDNA amplification using *Histone3* gene specific primers and then good quality cDNA was used for qRT-PCR. cDNA was mixed with 2x SYBR Green PCR Master Mix (Applied Biosystem) with gene- and homeolog-specific primers (shown in the following table). *Histone3* was used as internal control. qRT-PCR reactions were carried out using Bio-Rad C1000 Touch^TM^ Thermal Cycler (Bio-Rad Laboratories, Inc.) with the following conditions: 95 °C for 10 min followed by 40 cycles of 95 °C for 15 s, 60 °C for 1 min and plate read. Melting-curve analysis was performed at the end of reaction to ensure a single product. Three biological replicates and three technical replicates were used for each type of sample. Using 2^−△△^C_T_ method described by Livak and Schmittgen (2001) relative expression levels were quantified.

**Primers used to conduct qRT-PCR analyses on *CGF1*, *CGF2* and *GoPGF* (synonym *CGF3*) genes and respective homeologs.**

| Primer | Sequence 5' to 3' |
| --- | --- |
| A11CGF1-QF | CCAGCTTCGGTCCAGTTCCTTA |
| A11CGF1-QR | GAGGAATCTTTCTTGGTTTGGTTATTGTTA |
| D11CGF1-QF | AACCCCAGCTTCGATCCAGTTTCTTG |
| D11CGF1-QR | AGGAATCTTTCTTGGTTTGGTTATTGCTG |
| A01CGF2-QF | CAGATAGAATGAGTAGTGCTGCAAT |
| A01CGF2-QR | GGCCTCAGTGAAGAAATCATCG |
| D01CGF2-QF | CAGATAGAATGAGTAGTGCTGCAAC |
| D01CGF2-QR | CAATTGGCCTCAGTGAAGAAATCATCA |
| A12CGF3-QF | TGTGAAGATAGTAGGATCCGAAGCT |
| A12CGF3-QR | GTAGGGACTCTGACAACAACATCC |
| D12CGF3-QF | TGTGAAGATAGTAGGATCCGAAGCC |
| D12CGF3-QR | GTAGGGACTCTGACAACAACATCT |

**Sequencing of *CGF* genes in both A and D genomes of GVS4 and GVS5**

PCR was performed using Phusion polymerase (NEB), on genomic DNA isolated from GVS4 and GVS5 lines using primers specific to each homeolog of the three *CGF* genes (primers shown in the following table). Amplified products were gel-eluted and sequencing reactions were performed using the BigDye® Terminator v3.1 Cycle Sequencing Kit (Thermo Fisher Scientific, Waltham, MA) and samples were run on ABI 3130xl Genetic Analyzer. In addition to the primers that were used for amplification, several gene specific primers that are common to both A and D homeologs, primers that anneal to the transposon in *ACGF3* gene of the glandless GVS5 line, and the additional promoter sequence of *DCGF3* gene were used to sequence the selected genes. Obtained sequences were aligned and analyzed to identify the mutations using SnapGene software.

**Primers used for sequencing of three *CGF* genes from A and D subgenomes of glanded (GVS4) and glandless (GVS5) cotton plants.**

| Primer | Sequence 5' to 3' | Purpose |
| --- | --- | --- |
| A11GhCGF-P-4F | GTTATTTGATTGCTTCGTCAGTTACG | to sequence *CGF1* gene both homeologs |
| GhCGF-prom1.6kb-R | GTTTCCTATACTAAACTCAAGAGG |  |
| GhCGF-R1 | TTACTGCAGATCTAGCCTCCTGAG |  |
| GhCGF-R2 | GAGCGTAGAATCTGTGGTTCAGC |  |
| GhCGF-R3 | GCAACTCATGAGCACCAGTTAACC |  |
| GhCGF-R4 | TCCTGGGAAAAGGAAACCAGAG |  |
| GhCGF_prom1kb-R | TGTCACATTAGCATGAGGTACATGTGG |  |
| D11GhCGF-prom-2F | TAAGGTACACGAGGCACAGCACAC |  |
| CGF2-C-2F | AGAGAGTGAATCGTACTTCTTCTGC | to sequence *CGF2* gene both homeologs |
| CGF2-C-F | ATGATGAACGTCGACGACGTCC |  |
| CGF2-C-R | TCGCTTGAAGATTCGACATATGGTCC |  |
| CGF2-P-2F | GCAACCCTACTCCTATACTTCAATCTAG |  |
| CGF2-P-3F | GCTAGATGTGGTGTTGCCTCAC |  |
| CGF2-P-F | CAAGAATAGTCTAAGCTTCTCTAGCAAATGATC |  |
| CGF2-T-2R | ACTGGAGTACATCCATGTCAGTCTC |  |
| CGF3-cds-2F | AGTTCTGGGATCAACAACAGCCTG | to sequence *CGF3* gene both homeologs |
| CGF3-cds-3F | GGCCAAAGACAGTGGAAGTTGATG |  |
| CGF3-cds-F | ATGTCTTCCTCTTCTTCGTCTTCTC |  |
| CGF3-prom-2F | GGTTTCTTGAATCTAGTGAAGGATTGATTGTTG |  |
| CGF3-prom-3F | TTGCAAATTGAGAGAGTGATCATTGAGAC |  |
| CGF3-prom-4F | CATGAGTGGAGGGGTTAAGACGCC |  |
| CGF3-Tn-2F | TGACACTGCTAGTGCAGTCACTCTG | to sequence the transposon in the coding region of *ACGF3* gene in the glandless GVS5 |
| CGF3-Tn-3F | GACACAAGCATCATAGTCACATCTTGTG |  |
| CGF3-Tn-4F | TAACTGGAAGGTTCTATACCAATGGACTC |  |
| CGF3-Tn-5F | CAAATAACAAGCAGTATTAACAGCTTCAGC |  |
| CGF3-Tn-6F | ATATGCCATAACTTCGTGGTGTCAG |  |
| CGF3-Tn-7F | TTCTTGGACTGCGATCTAGGATGG |  |
| CGF3-Tn-8F | GCAATCCTTGTTGAACCAGCACT |  |
| CGF3-Tn-F | AAGCCATTTCTTAACAAATCTCCACCTTG |  |
| D12.CGF3.epro-F | AGCTCAATTTGGGGAGTTTACTTGC | to sequence the additional ~2 kb promoter sequence of the *DCGF3* in glanded and glandless cotton |
| D12.CGF3.epro-F2 | GTAAGTTCCACAAAGGAAAACTCAACAC |  |
| D12.CGF3.epro-F3 | CATAACCTTCCTTAGGTTGACCTCG |  |
| D12.CGF3.epro-F4 | GAATCACATGGTCTGGATCCTCATAG |  |
| D12.CGF3.epro-F5 | AGAAACACTGATTGGCGGTTC |  |
| D12.CGF3.epro-R | GGAATGTAATACCCTGTCCAACGTAG |  |
| D12.CGF3.epro-R2 | CGATATTGTGTATGTTTGTGTGATGC |  |

**Sequencing of *GoPGF* (synonym *CGF3*)** **gene from four other glandless lines**

Four other glandless lines, Acala glandless, NM13P1088, NM13P1115 and NM13P1118 were used to determine the cause of glandless phenotype in each. PCR reactions were performed on genomic DNA from each of the four glandless lines to amplify the *GoPGF* (synonym *CGF3*) gene from both A and D subgenomes, using primers specific to each homeolog (primers shown in the table presented above). Amplified fragments were gel eluted and sequenced. Sequences obtained were analyzed with SnapGene software.

**Promoter activity evaluation of D subgenome *GoPGF* (synonym *CGF3*)** **homeolog in the glanded and glandless cotton**

Approximately 2.1 kb and 4.2 kb, upstream sequences from the transcription start codon of the D subgenome *GoPGF* (synonym *CGF3*) gene were PCR amplified from both glanded and glandless cotton plants using the primers listed in the following table. The templates used in these PCR reactions were amplicons that were specific to the D subgenome of either the glanded or glandless *GoPGF* (synonym *CGF3*) gene, previously generated for sequencing of the *GoPGF* (synonym *CGF3*) gene including promoter, coding sequence and the terminator. Each of the ‘promoter’ amplicons was then cloned into pCAMBIA 2301 vector to replace the CaMV 35S promoter that drove the expression of the reporter gene *gus*A. This cloning was done using the NEBuilder® HiFi DNA assembly cloning kit (#E5520S; NEB) as per manufacturer instructions. Each of these binary vectors was introduced into *A. tumefaciens* strain LBA4404. The pCAMBIA 2301 vector, wherein *gus*A is under the control of CAMV 35S promoter was used as a control. Each of the *Agrobacterium* strains were used individually to infect cotton seedling explant to obtain stable transformed callus cultures (Rathore et al., 2015). Histochemical GUS assay was performed on the stably transformed callus tissue and GUS activity was examined at five weeks after transformation of cotyledon, hypocotyl and cotyledonary petiole explants (Jefferson, 1987; Jefferson et al., 1987).

**Primers used to amplify *D*  promoter sequences of ~2.1 and ~4.2 kb sizes from glanded and glandless cotton. The amplicon was used to generate reporter gene constructs using DNA assembly method (NEB), to evaluate promoter activity. Note that the reverse primer is common for both the promoter sizes.**

| Promoter fragment | Primer | Sequence 5' to 3' |
| --- | --- | --- |
| ~ 2.1 kb promoter | 2301-DCGF3_pro_frag1-F | TCGAGCTCGGTACCCGGGGATCCTCTAGAGTCGACCTGCAGCAAACCATCAACAAGACTACGTTGGAC |
|  | DCGF3_promoter_frag1-R | GAAGGAGAAAAACTAGAAATTTACCCTCAGATCTACCATAAGCTTTATTGAATATGATAGTGTGTACTACTGTTTTTCAAAGAGAAAAAAG |
| ~ 4.2 kb promoter | DCGF3_l.pro.F1-F | TCGAGCTCGGTACCCGGGGATCCTCTAGAGTCGACCTGCAGCTTCCCTATAACACCCCAATCCACG |
|  | DCGF3_promoter_frag1-R | GAAGGAGAAAAACTAGAAATTTACCCTCAGATCTACCATAAGCTTTATTGAATATGATAGTGTGTACTACTGTTTTTCAAAGAGAAAAAAG |

**CRISPR/Cas9-mediated knockout of *CGF2* and *GoPGF* (synonym *CGF3*) genes**

*CGF2* and *GoPGF* (synonym *CGF3*) genes were targeted for knockout using the CRISPR/Cas9 system. Base vectors to clone the guide sequences into the binary vectors were kindly provided by Daniel Voytas’ laboratory. Two separate sgRNAs were used to target each of the *CGF* genes to improve our chances of getting a total knockout (given that cotton is a tetraploid, even a single-copy gene will have four targets in its genome). To design the guide sequences, sgRNAScorer (Chari et al., 2015) and WU-CRISPR (Wong et al., 2015) tools were used. Based on the predicted scores from these tools, two guide RNA sequences were selected for *CGF2* gene (Table S5). For *GoPGF* (synonym *CGF3*) gene, WU-CRISPR did not predict any guide sequence in the desired region. However, using the sgRNAScorer, three potential guide sequences were identified (Table S5). Initially, each guide sequence was cloned into either pTC241 or pTC242 plasmid. The promoters regulating the expression of sgRNA in these vectors are AtU6 and At75L, respectively (Voytas et al., 2015). Guide sequences used to target *CGF2* and *GoPGF* (synonym *CGF3*) are listed in Table S5. The two sgRNA cassettes were incorporated into plasmid pCGS754. The final assembled vector contains *nptII* expression cassette for selection, a Cas9 expression cassette and two sgRNA cassettes. The binary vector LCT236 contains CGF2-guide-1, CGF2-guide-2 as guide sequences and targeted *CGF2* genes. While LCT237 contains CGF3-guide-1, CGF3-guide-2 as guide sequences, and LCT238 contains CGF3-guide-2, CGF3-guide-3 as guide sequences and targeted *GoPGF* (synonym *CGF3*) genes. Thus, one of the two sgRNAs used in assembling LCT237 and LCT238 was common between them. Each construct was mobilized into *Agrobacterium* *tumefaciens*, strain LBA4404 that was used to transform and generate cotton plants as per our laboratory protocol (Rathore et al., 2015).

Targeted disruption of the *CGF* genes was expected to have a negative impact on the formation of glands and terpenoids that accumulate within them. Selected plants showing such a phenotype were sequenced to characterize mutations in their respective target genes as described by Wang et al. (2018). A PCR amplicon that encompasses the two target sites in each gene was generated from the genomic DNA isolated from the leaves of selected T0 plants. Each set of PCR primers contained a unique combination of barcodes for identification purposes (Table S6). PCR amplifications were performed using Phusion polymerase (NEB), on genomic DNA isolated from regenerated lines targeted with LCT236 and LCT237 constructs. PCR amplification conditions were as follows: 95 °C for 5 min, then 35 cycles of 95 °C for 30 sec, 58 °C for 30 sec, 72 °C for 45 sec, and finally 10 min at 72 °C. PCR products were loaded on agarose gel, purified using gel extraction kit and amplicons were pooled in equimolar ratio. These pooled amplicons were paired-end sequenced (2 x 250 bp) on Illumina HiSeq2500 platform. After sequencing, reads were trimmed and filtered using Trimmomatic software to filter out the low-quality reads (Bolger et al., 2014), paired sequences were merged using FLASH2 (Magoč and Salzberg, 2011) with default parameters and demultiplexed using internal barcodes, and CRISPResso (Pinello et al., 2016) was used to ascertain the nature of mutations in the amplicons.

**Construction of A genome *GoPGF* (synonym *CGF3*)** **overexpression vector and transformation**

*ACGF3* coding sequence was amplified and placed downstream of CaMV 35S promoter by replacing the *gus*A gene in the binary vector pCAMBIA2301. This *ACGF3* overexpression construct was then used to transform various cotton seedling explants using our laboratory protocol (Rathore et al., 2015). Individual transgenic events, in the form of kanamycin-resistant calli, were examined for terpenoid content as described earlier.

**References**

Chari, R., Mali, P., Moosburner, M. and Church, G.M. (2015) Unraveling CRISPR-Cas9 genome engineering parameters via a library-on-library approach. *Nat Meth* **12**, 823-826.

Dinesh-Kumar, S.P., Anandalakshmi, R., Marathe, R., Schiff, M. and Liu, Y. (2003) Virus-Induced Gene Silencing. In: *Plant Functional Genomics* (Grotewold, E. ed) pp. 287-293. Totowa, NJ: Humana Press.

Gao, X., Britt Jr, R.C., Shan, L. and He, P. (2011) Agrobacterium-Mediated Virus-Induced Gene Silencing Assay In Cotton. *Journal of Visualized Experiments : JoVE*, 2938.

Jefferson, R.A. (1987) Assaying chimeric genes in plants: the GUS gene fusion system. *Plant molecular biology reporter* **5**, 387-405.

Jefferson, R.A., Kavanagh, T.A. and Bevan, M.W. (1987) GUS fusions: beta-glucuronidase as a sensitive and versatile gene fusion marker in higher plants. *The EMBO Journal* **6**, 3901-3907.

Livak, K.J. and Schmittgen, T.D. (2001) Analysis of Relative Gene Expression Data Using Real-Time Quantitative PCR and the 2−ΔΔCT Method. *Methods* **25**, 402-408.

Magoč, T. and Salzberg, S.L. (2011) FLASH: fast length adjustment of short reads to improve genome assemblies. *Bioinformatics* **27**, 2957-2963.

Pinello, L., Canver, M.C., Hoban, M.D., Orkin, S.H., Kohn, D.B., Bauer, D.E. and Yuan, G.-C. (2016) Analyzing CRISPR genome-editing experiments with CRISPResso. *Nature biotechnology* **34**, 695.

Rathore, K.S., Campbell, L.M., Sherwood, S. and Nunes, E. (2015) Cotton (Gossypium hirsutum L.). In: *Agrobacterium Protocols: Volume 2* (Wang, K. ed) pp. 11-23. New York, NY: Springer New York.

Stipanovic, R.D., Altman, D.W., Begin, D.L., Greenblatt, G.A. and Benedict, J.H. (1988) Terpenoid aldehydes in upland cottons: analysis by aniline and HPLC methods. *Journal of Agricultural and Food Chemistry* **36**, 509-515.

Voytas, D.F., Atkins, P. and Baltes, N.J. (2015) Engineering Plant genomes using CRISPR/Cas systems. 2015/0167000 A1.

Wang, P., Zhang, J., Sun, L., Ma, Y., Xu, J., Liang, S., Deng, J., Tan, J., Zhang, Q. and Tu, L. (2018) High efficient multisites genome editing in allotetraploid cotton (*Gossypium hirsutum*) using CRISPR/Cas9 system. *Plant Biotechnology Journal* **16**, 137-150.

Wong, N., Liu, W. and Wang, X. (2015) WU-CRISPR: characteristics of functional guide RNAs for the CRISPR/Cas9 system. *Genome biology* **16**, 1.
